# Effects of Acute Sleep Loss on Physical Performance: A Systematic and Meta-Analytical Review

**DOI:** 10.1007/s40279-022-01706-y

**Published:** 2022-06-16

**Authors:** Jonathan Craven, Danielle McCartney, Ben Desbrow, Surendran Sabapathy, Phillip Bellinger, Llion Roberts, Christopher Irwin

**Affiliations:** 1 School of Health Sciences and Social Work, Griffith University, Southport, QLD 4222 Australia; 2 Queensland Academy of Sport, Nathan, QLD Australia; 3 School of Psychology, Faculty of Science, University of Sydney, Sydney, NSW Australia; 4 Griffith Sports Science, Griffith University, Gold Coast, QLD Australia; 5 School of Human Movement and Nutrition Sciences, University of Queensland, Brisbane, QLD Australia

## Abstract

**Background:**

Sleep loss may influence subsequent physical performance. Quantifying the impact of sleep loss on physical performance is critical for individuals involved in athletic pursuits.

**Design:**

Systematic review and meta-analysis.

**Search and Inclusion:**

Studies were identified via the Web of Science, Scopus, and PsycINFO online databases. Investigations measuring exercise performance under ‘control’ (i.e., normal sleep, > 6 h in any 24 h period) and ‘intervention’ (i.e., sleep loss, ≤ 6 h sleep in any 24 h period) conditions were included. Performance tasks were classified into different exercise categories (anaerobic power, speed/power endurance, high-intensity interval exercise (HIIE), strength, endurance, strength-endurance, and skill). Multi-level random-effects meta-analyses and meta-regression analyses were conducted, including subgroup analyses to explore the influence of sleep-loss protocol (e.g., deprivation, restriction, early [delayed sleep onset] and late restriction [earlier than normal waking]), time of day the exercise task was performed (AM vs. PM) and body limb strength (upper vs. lower body).

**Results:**

Overall, 227 outcome measures (anaerobic power: *n* = 58; speed/power endurance: *n* = 32; HIIE: *n* = 27; strength: *n* = 66; endurance: *n* = 22; strength-endurance: *n* = 9; skill: *n* = 13) derived from 69 publications were included. Results indicated a negative impact of sleep loss on the percentage change (%_Δ_) in exercise performance (*n* = 959 [89%] male; mean %_Δ_ =  − 7.56%, 95% CI − 11.9 to − 3.13, *p* = 0.001, *I*^2^ = 98.1%). Effects were significant for all exercise categories. Subgroup analyses indicated that the pattern of sleep loss (i.e., deprivation, early and late restriction) preceding exercise is an important factor, with consistent negative effects only observed with deprivation and late-restriction protocols. A significant positive relationship was observed between time awake prior to the exercise task and %_Δ_ in performance for both deprivation and late-restriction protocols (~ 0.4% decrease for every hour awake prior to exercise). The negative effects of sleep loss on different exercise tasks performed in the PM were consistent, while tasks performed in the AM were largely unaffected.

**Conclusions:**

Sleep loss appears to have a negative impact on exercise performance. If sleep loss is anticipated and unavoidable, individuals should avoid situations that lead to experiencing deprivation or late restriction, and prioritise morning exercise in an effort to maintain performance.

**Supplementary Information:**

The online version contains supplementary material available at 10.1007/s40279-022-01706-y.

## Key Points

**Table:** 

Acute sleep loss negatively impacts next-day exercise performance
The magnitude and significance of the impact are dependent on the sleep-loss protocol preceding exercise, with sleep deprivation and late-restriction (earlier than normal waking) protocols demonstrating a consistent negative influence
The time awake prior to performing exercise was found to be an influential factor
Exercise tasks performed in the PM were consistently negatively affected by sleep loss, while tasks performed in the AM were largely unaffected

## Introduction

Sleep is essential to maintain physical and mental health. It has been shown to promote memory [1], regulate emotions [2], enhance metabolic functions [3], improve energy balance, and moderate the immune system [4], and may play a pivotal role in the stress–recovery balance, via its influence on the activity of the hypothalamic–pituitary–adrenal axis [5]. Despite this knowledge, ~ 45% of the Western adult population fail to obtain the recommended 7–9 h of sleep each night [6]. Sleep loss is often driven by lifestyle choices that reduce available sleep time, such as evening social activities [7], exposure to artificial light prior to sleep [8], consumption of caffeinated beverages [9], and smoking [10]. Stress and anxiety [11], medical conditions/illness [12], and genetic traits [13, 14] can also contribute to sleep loss. Certain populations, including professional athletes [15–24], shift workers [25] and military personnel [26], appear particularly susceptible to sleep loss. For athletes, sleep loss may be exacerbated by early morning training sessions [27, 28], training or competing at altitude (> 2000 m) [29], travel (late night and early morning departures) [30, 31], and the use of caffeine as an ergogenic aid [32].

Insufficient sleep can result in a significant personal and societal burden, including adverse effects on wellbeing [33], productivity [34] and safety [35]. For those who are physically active or involved in athletic pursuits, sleep loss may also influence acute training adaptations and exercise performance outcomes [17, 36, 37]. The consequences of sleep loss (e.g., altered training adaptations, increased workplace accidents [38, 39]) are likely to have multiple aetiologies. Negative consequences may result from a decrease in muscular strength [40] and/or endurance [41], change in mood (e.g., decreased motivation) [42], an increase in perceived effort [43, 44], changes to cognitive processing ability (e.g., decision making, executive function) and/or a reduction in fine motor skills [45], or a combination of these factors.

**Fig. 1:**
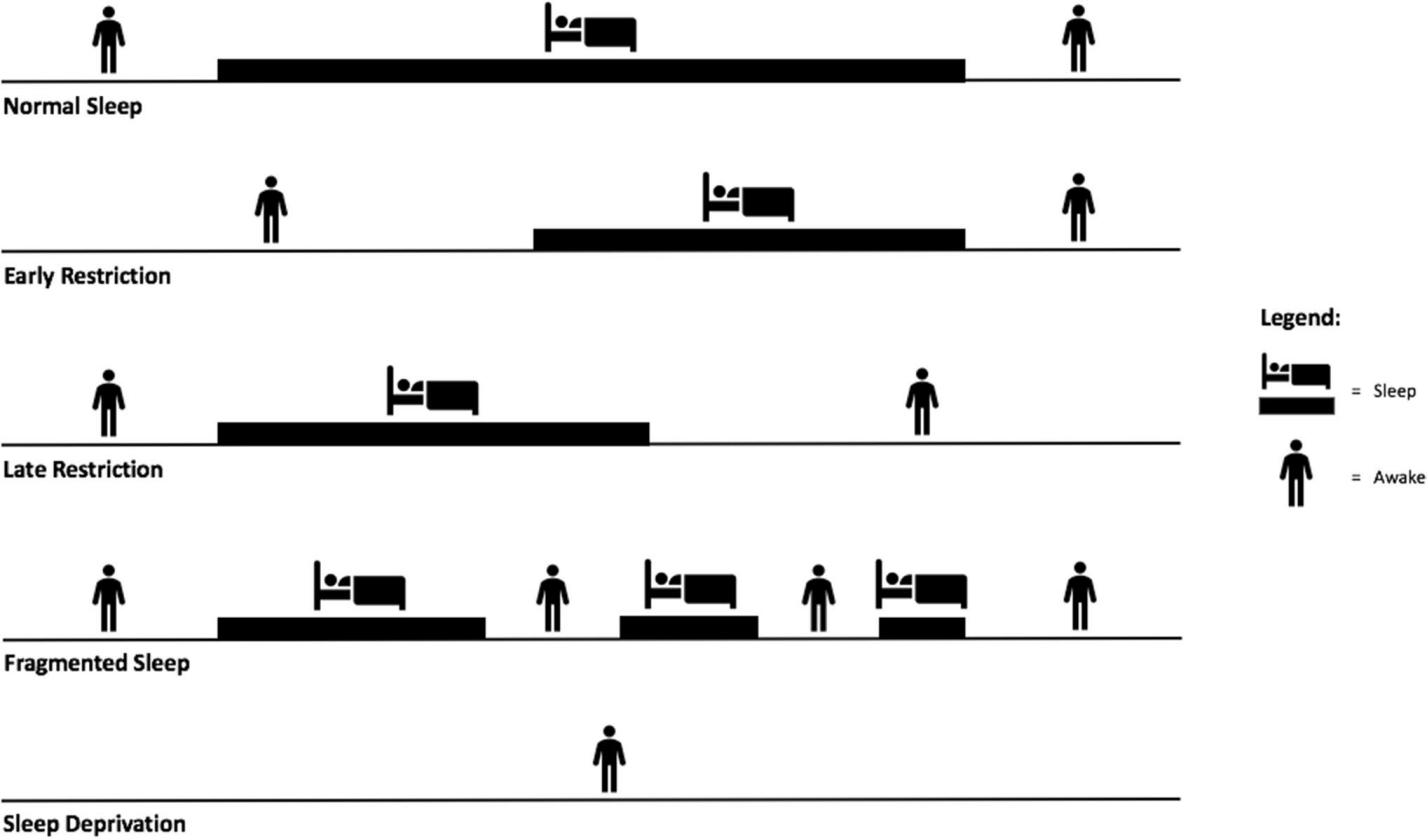
Types of sleep loss encountered

Sleep has two distinct dimensions: quantity and quality. However, sleep loss is more often measured in terms of duration, given the challenges associated with accurately determining sleep quality in most situations [46]. Sleep deprivation is a general term used to describe a period of extended wakefulness, often related to circumstances when an individual is unable to obtain any sleep across a period of ≥ 24 h [47]. Restricted sleep (also referred to as ‘partial sleep deprivation’) occurs when an individual has the opportunity to sleep, but this is limited in duration from their normal sleep habit [47] and is often a result of delayed sleep onset (sometimes termed ‘early restriction’), earlier than normal waking (sometimes termed ‘late restriction’), or fragmented sleep, which is when one or more nocturnal awakenings occur [48] (Fig. 1 depicts the different types of sleep loss). The amount (e.g., deprivation/restriction) and type (e.g., early restriction/late restriction) of sleep loss incurred may have some influence on the magnitude of effect that insufficient sleep has on physical performance [49–52].

The influence of sleep loss on physical performance has received considerable scientific attention. Studies have investigated the effects of sleep loss on performance in different exercise tasks (based on predominant physical attributes), including strength [41, 53–55], anaerobic power/capacity [56–62], endurance [41, 57], and those requiring a high level of precision (e.g., skill activities [45, 63]). The influence of contextual factors has also been explored, including the timing of exercise following sleep loss (e.g., morning vs. evening exercise) [45, 53, 62, 64]; duration of sleep loss [65]; early- vs. late-sleep restriction protocols [66, 67]; and exercise characteristics themselves (acute, chronic, type and timing) [64, 68, 69]. While several reviews have summarised these effects [36, 70, 71], only one employed meta-analytical techniques to synthesise the outcomes [72]. However, this particular review was conducted over two decades ago, and many studies conducted since have improved our understanding of sleep loss and its impact on physical performance.

Therefore, the aim of this systematic review was to summarise the available literature investigating the effects of acute sleep loss (≤ 6 h sleep in any 24 h period) on exercise performance and quantify the magnitude of effects using meta-analytical techniques. The influence of certain contextual factors (e.g., exercise type, time of day, sleep-loss duration) was also explored.

## Methods

The methodology of this review was developed in accordance with the Preferred Reporting Items for Systematic Reviews and Meta-Analysis Protocols 2015 statement [73] and registered at the International Prospective Register of Systematic Reviews (PROSPERO; identification code: CRD42020211824).

### Literature Search

**Fig. 2:**
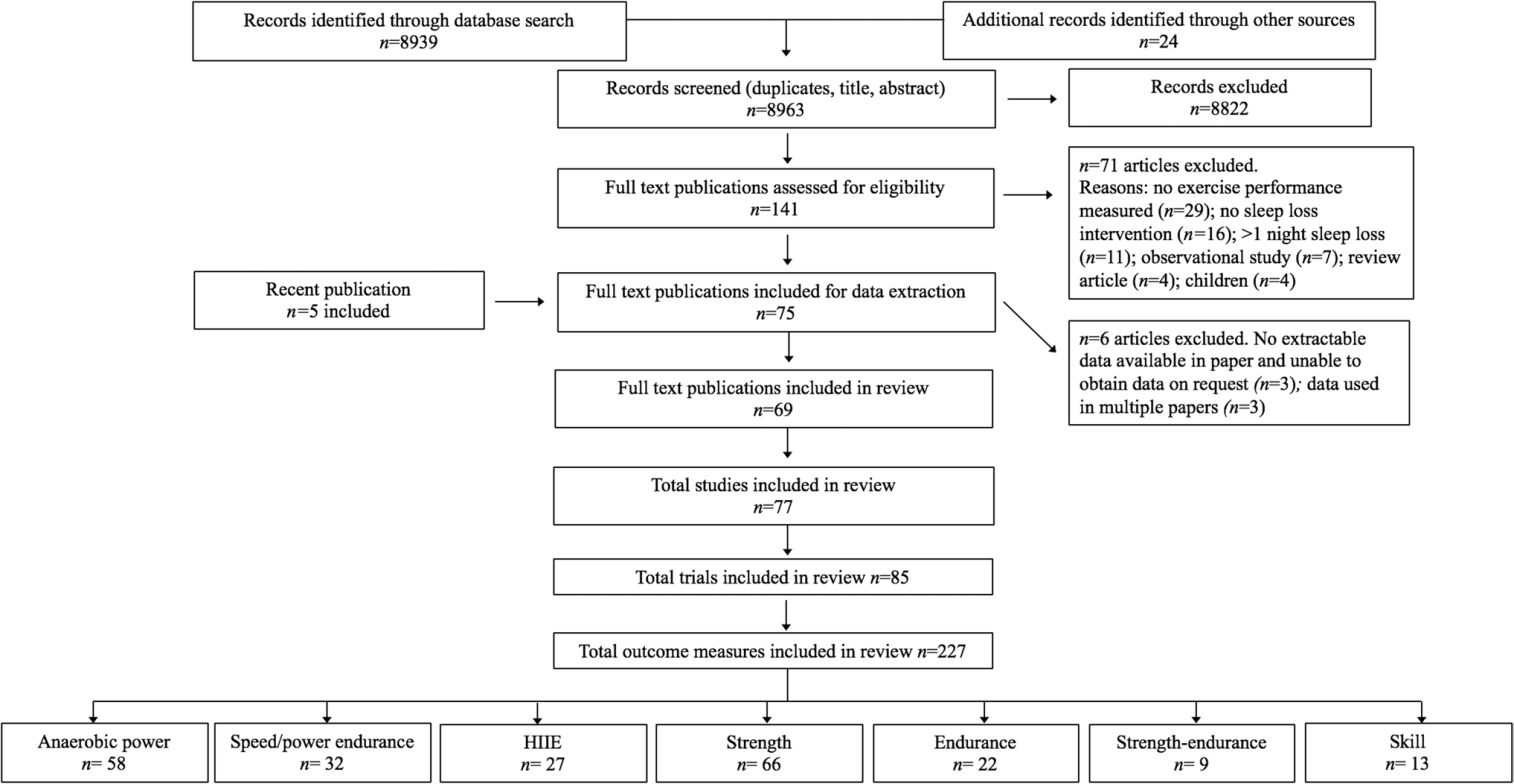
PRISMA flow chart (study selection methodology). Some publications contained multiple participant pools. In these instances, the individual participant pools were termed ‘studies’. Some studies investigated the influence of more than one sleep-loss protocol (i.e., deprivation, early or late restriction). In these instances, the separate study arms were treated as individual investigations, and termed ‘trials’. Each individual task from a given trial was termed ‘outcome measure’. ESM Table S1 provides the original search breakdown; ESM Table S2 provides the origin of included publications; and ESM Table S3 provides the reference and reason for exclusion of full-text publications. *HIIE* high-intensity interval exercise, *ESM* electronic supplementary material

Studies were identified by searching the Web of Science (via Thomas Reuters), Scopus, and PsycINFO online databases from inception until September 2020 using the Boolean expression: (sleep restriction OR sleep deprivation OR sleep loss OR wakefulness) AND (exercis* OR performance AND NOT animal* OR rat* OR mice). The star symbol (*) was used to capture derivatives (by suffixation) of the search terms. Two investigators (JC and CI) independently screened the potential publications to identify relevant texts. Initially, all irrelevant titles were discarded. The remaining publications were then systematically screened for eligibility by abstract and full text. The decision to include or discard potential publications was made between two investigators (JC and CI) and any discrepancies were resolved in consultation with a third investigator (DM). One investigator (JC) also hand-searched the reference lists of included publications and performed a forward citation search of two previous systematic reviews [36, 71] to ensure all relevant publications were captured. An updated search was also conducted on 31 December 2021 to capture the most recent publications. Full details of the screening process are illustrated in Fig. 2.

### Inclusion and Exclusion Criteria

Original research studies that met the following criteria were included in this review: (1) full-text original articles written in English; (2) controlled trials employing repeated measures experimental designs; (3) human studies on adult (≥ 18 years of age) men and women with no known medical conditions and comorbidities; and (4) measured performance on an exercise/physical task (e.g., Wingate test, squat jump) under ‘sleep loss’ (i.e., ≤ 6 h sleep in any 24 h period) and ‘control’ (i.e., normal sleep, considered as > 6 h in any 24 h period) conditions.

Studies were excluded from the review if (1) a between-subject experimental design was employed and no baseline measurements were performed following ‘normal sleep’; (2) sleep-loss protocol was not ‘acute’ (i.e., it was sustained over multiple nights); (3) stimulants or sedatives were administered (e.g., caffeine, L-tryptophan, or modafinil) during the trial; (4) exercise prior to sleep intervention was not matched across conditions;^1^ (5) participants reported abnormal sleep behaviours (e.g., sleep disorder, shift-worker); (6) participants reported recent international travel with experience of jetlag; (7) exercise performance was measured after a period of recovery sleep (sleep latency tests were not considered ‘recovery sleep’); and (8) exercise performance data were not adequately reported (i.e., mean ± standard deviation [SD] was not reported or could not be derived).

In the event that data were not adequately reported, the corresponding author was contacted via email in an attempt to retrieve the missing data. Where data were presented in graphical format only, a web-based tool (‘WebPlotDigitizer’, https://apps.automeris.io/wpd/) was used to extract numeric values.

Several publications identified in the literature search contained more than one intervention and control comparison that was eligible for inclusion. Some publications contained multiple participant pools. In these instances, the individual participant pools were termed ‘studies’. Some studies investigated the influence of more than one sleep-loss protocol (a combination of either deprivation, early or late restriction). In these instances, the separate study arms were treated as individual investigations, and termed ‘trials’. As single trials sometimes measured serial performance (i.e., multiple times across the trial) and/or used several tasks that generated multiple outcomes, each one could contribute multiple effect estimates to the review (note, multilevel models were used to account for dependency of effect estimates in statistical analyses [74]; refer to Sect. 2.7 ‘Statistical Analyses’). In these instances, each individual effect estimate from a given trial was termed an ‘outcome measure’.

### Exercise Task Classifications

Each exercise task was reviewed by two investigators (JC and PB) and allocated into one of the following seven categories: anaerobic power, speed/power endurance, high-intensity interval exercise (HIIE), strength, endurance, strength-endurance, and skill. The allocation criteria are defined in Table 1. All discrepancies were resolved in consultation with a third investigator (SS).

**Table 1:** Exercise task categories

Exercise task category	Description of exercise task	Example task
Anaerobic power	Duration ≤ 6 s performed at maximum effort	Wingate (peak power); CMJ; squat jump; 20 m sprint
Speed/power endurance	Maximal continuous exercise—duration > 6 s but < 90 s	30 s Wingate test (mean power); 5 m multiple shuttle test (peak distance); TTE at predetermined workload; repeated CMJ—mean jump height
HIIE	Requires near-maximal effort (~ 45 s) with brief periods of recovery (≤ 4.5 min)	Yo-Yo intermittent recovery test level 1; 5 m multiple shuttle test (total distance)
Endurance	Continuous exercise ≥ 120 s	TTE for incremental exercise test; peak power output at exhaustion during incremental exercise test; 3 km TT
Strength	Maximum force development during a single effort	1RM; MVC (e.g., hand-grip strength test, knee extension)
Strength-endurance	Resistance task ≥ 2 repetitions or > 5 s sustained contraction	Number of repetitions performed at 85% of 1RM; 30 s MVC; knee-extension fatiguing task
Skill	Task that requires high cognitive component for execution	Tennis serving; rugby passing; free-throw shooting (basketball); shooting

*HIIE* high-intensity interval exercise, *TT* time trial, *RM* repetition maximum, *MVC* maximum voluntary contraction, *TTE* time to exhaustion, *CMJ* counter-movement jump

Some studies included in this meta-analysis assessed the influence of sleep loss on more than one performance task, either belonging to the same category [40, 41, 53, 58, 59, 63, 64, 75–84], or different categories [41, 43, 44, 56–59, 61, 62, 64, 65, 75, 77–81, 85–102]. For example, Souissi et al. [78] measured anaerobic power in two separate tasks (i.e., squat jump and Wingate test). In these cases, effect estimates were derived for all eligible tasks.

Measures of residual muscular fatigue (i.e., those obtained within minutes of completing an initial performance test) [64, 89] were not included in the review. However, serial measures were accepted if the preceding test was deemed unlikely to have influenced performance on the subsequent test. For example, if the preceding test used different muscle groups (e.g., knee extensor maximum voluntary contraction (MVC) and knee flexion MVC) [53, 76, 82, 83] or a contralateral muscle group [82], or if the same test was repeated at separate times of the day (e.g., once at 0600 h, then at 1700 h) [43, 53, 62, 64, 79–81, 90], then each measurement was included and considered as a separate ‘outcome measure’.

### Primary and Secondary Research Outcomes

The primary outcome in this investigation was the percentage change in exercise performance (%_Δ_EP) following sleep loss (i.e., sleep restriction or sleep deprivation), calculated using the following formula:

$$\%_{\Delta} {\text{EP}} = \frac{{\left( {{\text{EP}}\; {\text{sleep}}\;{\text{ loss}} - {\text{EP}}\; {\text{normal}}\;{\text{ sleep}}} \right)}}{{{\text{EP}}\; {\text{normal}}\;{\text{ sleep}}}} \times 100,$$

where ‘EP sleep loss’ was exercise performance measured following sleep loss and ‘EP normal’ was exercise performance measured under control conditions.

### Data Extraction

Data were extracted in accordance with the Cochrane Handbook for Systematic Reviews of Interventions Checklist of Items to Consider in Data Collection or Data Extraction [103] and entered into a Microsoft Excel spreadsheet (Microsoft Corporation, Redmond, WA, USA). Extracted data included (1) participant characteristics (e.g., training status, age, body mass, sex, aerobic power [$\dot{V}$O_2peak_]); (2) pretrial standardisation procedures; (3) the sleep-loss protocol (e.g., deprivation, restriction); (4) sleep location (e.g., laboratory, home); (5) the instrument used to monitor and record hours slept; (6) protocol used to assess exercise performance; (7) timing of tests relative to the sleep/wake cycle; and (8) whether or not participants were fed/fasted prior to performance testing.

### Quality Assessment

The included publications were assessed based on their methodological quality using the Rosendal Scale (see Table 2 in the article by van Rosendal et al. [104]). This scale, which combines the Jadad scoring system [105], PEDro scale [106] and Delphi List [107], assesses a number of factors associated with the minimisation of experimental bias (e.g., blinding, participant selection, randomisation, data reporting). Excellent methodological quality is indicated by a Rosendal score ≥ 60% [105]. Scoring was determined by dividing the number of ‘yes’ responses by the total number of applicable items. Scores were compared between two investigators (JC and CI) conducting the assessments and any discrepancies were resolved (with a third investigator consulted [DM] if agreement could not be reached). As such, the final score is an agreed rating for each publication.

**Table 2:** Meta-analysis results for effect of sleep loss on exercise performance

Exercise category	Outcomes, *n*	Exercise performance percentage change		Heterogeneity	
		Mean (95% CI)	*p *value	*I*^2^ value	*p *value
*All categories*					
Overall	227	− 7.56 (− 11.9 to − 3.13)	0.001	98.1	< 0.001
*Sleep-loss condition*					
Deprivation	97	− 5.25 (− 8.01 to − 2.48)	< 0.001	96.5	< 0.001
Restriction	130	− 8.59 (− 13.6 to − 3.61)	0.001	98.3	< 0.001
Early restriction	62	− 5.85 (− 13.4 to 1.66)	0.125	93.5	< 0.001
Late restriction	60	− 7.39 (− 10.1 to − 4.66)	< 0.001	98.3	< 0.001
*Anaerobic power*					
Overall	58	− 6.26 (− 9.10 to − 3.41)	< 0.001	98.1	< 0.001
*Sleep-loss condition*					
Deprivation	25	− 6.39 (− 11.7 to − 1.09)	0.020	99.2	< 0.001
Restriction	33	− 5.99 (− 9.22 to − 2.77)	0.001	94.1	< 0.001
Early restriction	11	− 0.50 (− 2.00 to 1.00)	0.477	0.04	0.770
Late restriction	22	− 7.47 (− 11.1 to − 3.85)	< 0.001	95.4	< 0.001
*Speed/power endurance*					
Overall	32	− 2.90 (− 4.97 to − 0.82)	0.008	96.3	< 0.001
*Sleep-loss condition*					
Deprivation	12	− 2.93 (− 8.05 to 2.18)	0.233	91.1	< 0.001
Restriction	20	− 3.23 (− 5.94 to − 0.53)	0.022	96.4	< 0.001
Early restriction	7	0.49 (− 2.05 to 3.04)	0.652	24.4	0.366
Late restriction	13	− 4.38 (− 7.15 to − 1.62)	0.005	97.3	< 0.001
*HIIE*					
Overall	27	− 6.15 (− 10.5 to − 1.77)	0.008	98.9	< 0.001
*Sleep-loss condition*					
Deprivation	9	− 2.38 (− 12.1 to 7.32)	0.587	99.0	< 0.001
Restriction	18	− 8.77 (− 13.3 to − 4.27)	0.001	98.3	< 0.001
Early restriction	8	− 3.15 (− 9.68 to 3.37)	0.291	73.2	0.001
Late restriction	10^a^	− 11.5 (− 16.3 to − 6.71)	< 0.001	99.2	< 0.001
*Strength*					
Overall	66	− 2.85 (− 4.47 to − 1.23)	< 0.001	62.2	< 0.001
*Sleep-loss condition*					
Deprivation	29	− 3.00 (− 4.52 to − 1.48)	< 0.001	49.2	< 0.001
Restriction	37	− 2.77 (− 6.75 to 1.21)	0.167	74.9	< 0.001
Early restriction	26	− 1.16 (− 2.57 to 0.25)	0.102	0.02	0.952
Late restriction	11	− 4.45 (− 9.30 to 0.41)	0.068	83.7	< 0.001
*Endurance*					
Overall	22	− 5.55 (− 8.12 to − 2.99)	< 0.001	86.5	< 0.001
*Sleep-loss condition*					
Deprivation	14^a^	− 6.75 (− 10.3 to − 3.25)	< 0.001	91.2	< 0.001
Restriction	8	− 3.27 (− 5.06 to − 1.47)	0.004	< 0.001	0.914
Early restriction	2^a^	− 5.28 (− 9.17 to − 1.39)	0.008	0.00	0.798
Late restriction	3^a^	− 3.72 (− 6.96 to − 0.47)	0.025	< 0.001	0.620
*Strength-endurance*					
Overall	9	− 9.85 (− 19.6 to − 0.13)	0.048	85.4	< 0.001
Deprivation	6	− 6.06 (− 14.9 to 2.80)	0.139	45.6	0.255
Restriction	3	− 18.3 (− 35.6 to − 0.96)	0.045	88.3	0.001
*Skill*					
Overall	13	− 20.9 (− 27.0 to − 14.9)	< 0.001	94.1	< 0.001
*Sleep-loss condition*					
Deprivation	2^a^	− 20.9 (− 23.6 to − 18.2)	< 0.001	0.00	0.342
Restriction	11	− 21.0 (− 29.1 to − 12.9)	< 0.001	95.4	< 0.001
Early restriction	8	− 23.9 (− 33.6 to − 14.2)	< 0.001	94.8	< 0.001
Late restriction	1				

A negative effect estimate indicates a decrease in performance under the intervention condition (‘sleep loss’)

Deprivation: participants did not sleep for an extended period of time (i.e., whole night); restriction: total sleep time ≤ 6 h in any 24 h period—this category is a combination of early restriction, late restriction, fragmented sleep and non-specified sleep restriction protocols; early restriction: participants delayed sleep (i.e., went to sleep at a later time); late restriction; participants awakened earlier than normal

*HIIE* high-intensity interval exercise, *CI* confidence interval

^a^All outcomes were from independent studies and the meta-analysis was run without dependency levels (i.e., simple meta-analysis)

### Statistical Analyses

A series of multilevel meta-analyses and meta-regression analyses were performed using R Studio (version 4.0.1) with the metafor-package [108] and syntax adapted from Assink and Wibbelink [74]. A two-level meta-analysis is equivalent to a traditional random-effects analysis in which there is only one random effect. For the meta-analysis and meta-regression analysis when all seven exercise categories were combined, we added random effects at two additional levels to account for dependency among effect estimates derived from the (1) same studies; and (2) same exercise categories. Therefore, the four sources of variance modelled were: (Level 1) the sampling variance for the observed effect estimates; (Level 2) the variance between effect estimates derived from the same studies; (Level 3) the variance between effect estimates derived from the same exercise categories; and (Level 4) the variance between studies. The subgroup analyses (described in Sect. 2.7.1) accounted for dependency among effect estimates derived from the same studies only. An example of the accompanying R script (for the combined exercise category analysis) is available in electronic supplementary material [ESM] Appendix S1.

#### Weighted Mean Effect

Meta-analyses were performed to determine the influence of sleep loss (vs. control) on overall exercise performance (all exercise categories combined) and each respective exercise category (i.e., anaerobic power, speed/power endurance, HIIE, strength, endurance, strength-endurance, skill). Individual effect estimates were calculated as the %_Δ_ EP (as described in Sect. 2.4), where a negative effect estimate indicates a decrease in exercise performance under the intervention condition (‘sleep loss’). As the current review used the %_Δ_ EP (i.e., rather than the net difference), the SD of exercise performance change could not be determined via standard methods. Instead, *t *statistics (or *p *values) derived from paired *t *tests were used to calculate the SD of the percentage change in exercise performance (SD_Δ_). Where an exact value was quoted [45, 76, 88, 92, 109–111], the calculation was performed using the following formula [112]:

1 $${\text{SD}}_{\Delta} = \frac{{\left| {{{\%_{\Delta} {\text{EP}}}}} \right|}}{{t\;{\text{statistic}}}}{{ \times }}\surd n$$

where SD_Δ_ is the SD of the percentage change in exercise performance and *n* is the number of participants. Where only *p* > *x* or *p* < 0.05 was reported (and raw exercise performance data could not be retrieved), the missing *t*-statistic was imputed using the following formula:

2 $${\text{SD}}_{{\Delta }} = \sqrt {\left( {{\text{SD}}^{2}_{{\text{sleep loss EP}}} { } + {\text{ SD}}^{2}_{{\text{normal sleep EP}}} { }} \right){ }{-}\left( {2{ } \times { }R{ } \times {\text{SD}}_{{\text{ sleep loss EP}}} { } \times {\text{SD }}_{{\text{normal sleep EP}}} } \right),}$$

where SD_Δ_ is the SD of the net exercise performance change and R is the correlation coefficient. R was approximated (0.71) as the mean correlation coefficient calculated using raw exercise performance data from nine outcome measurements derived from seven publications [45, 76, 88, 92, 109–111], as indicated by Higgins and Green [112]. Sensitivity analyses were performed using R = 0.30 and 0.80 to test the robustness of the analysis to the imputed value. In addition, outcome measures were individually excluded (i.e., one-out method) to examine their influence on the weighted mean effect estimate. The imputed SD_Δ_ (net change) used to derive the *t*-statistic was calculated using the following formula:

3 $$t{\text{ - statistic}} = \frac{{{\text{mean EP}}_{{\text{sleep loss }}} - {\text{mean EP}}_{{\text{normal sleep}}} { }}}{{\left( {{\text{SD}}_{{\Delta }} { } \div { }\sqrt n } \right)}}.$$

Effect estimates were weighted by the inverse variance of the performance change and statistical significance was attained if the 95% confidence interval (CI) did not include zero. Heterogeneity was assessed using Cochran’s *Q*, the *I*^2^ index and the within- and between-cluster variance components (i.e., *σ*^2^). Significant heterogeneity was indicated by a *p *value < 0.05 for Cochran’s *Q* [113]. Subgroup analyses were performed to investigate the influence of (1) the sleep-loss protocol implemented (e.g., sleep deprivation, sleep restriction [i.e., the combination of early and late restriction, fragmented sleep and non-specified sleep restriction protocols]), and early and late restriction; (2) the timing of exercise following sleep loss (ante meridiem [AM] vs. post meridiem [PM]); and (3) body limb strength (upper- vs. lower-body strength), on %_Δ_EP. The time that body limb strength tasks were conducted following sleep loss and its impact on %_Δ_EP were also explored for each sleep-loss protocol implemented.

#### Meta-Regression Analysis

Restricted maximum likelihood (REML) multilevel simple meta-regression analyses were performed to determine whether the %_Δ_EP between treatments was influenced by the time awake prior to the exercise task (i.e., the number of hours from their last waking to the start of the exercise task). Regression analyses were examined for influential cases and outliers (i.e., studentised residuals, Cook’s distance and centred leverage values). Statistical significance was accepted as *p* < 0.05.

## Results

### Overview of the Included Studies and Study Quality

Seventy-five publications met the inclusion criteria; however six had to be excluded because data (1) could not be extracted (or retrieved) [114–116]; or (2) were reported in another included publication [117–119]. Therefore, 69 publications remained for analysis. These publications provided 77 individual ‘studies’ (i.e., eight additional participant pools [82, 90, 120–123]). Ten studies investigated the influence of more than one sleep-loss protocol (a combination of either deprivation, early or late restriction) [44, 57, 64–67, 81, 87, 124, 125]. This resulted in 85 trials, in which 14 measured the same exercise task(s) multiple times (twice, e.g., once at 0600 h, then at 1800 h [43, 53, 62, 64, 79–81, 90] or more than two times [45, 54, 55]). Thirty-six trials (derived from 23 studies) reported only one outcome measure [45, 49, 50, 54, 55, 66, 67, 110, 111, 116, 120–132], with the remaining trials yielding multiple outcome measures. This resulted in 227 separate outcome measures being included in the overall analysis. These outcome measures were further classified into their respective exercise categories (anaerobic power: *n* = 58; speed/power endurance: *n* = 32; HIIE: *n* = 27; strength: *n* = 66; endurance: *n* = 22; strength-endurance: *n* = 9; skill: *n* = 13). The location of the sleep protocol (i.e., slept in the laboratory or at home) and the method used to monitor sleep parameters (i.e., duration/quality) for each outcome measure are provided in ESM Table S4. Participant characteristics, mode of exercise, and timing of the exercise task are outlined in ESM Table S5; an overview of each included study is provided in ESM Appendix S2. Methodological quality assessment yielded an average Rosendal score of 67 ± 9%, with all but one publication [83] scoring ≥ 50%. Results of the quality assessment are shown in ESM Table S6.

### Overall Exercise Performance (All Exercise Categories)

Seventy-seven studies (*n* = 959; 89% male), providing 227 outcome measures, were included in this analysis. The overall weighted mean effect estimate (Table 2) indicated a negative influence of sleep loss on exercise performance (mean %_Δ_ =  − 7.56%, 95% CI − 11.9 to − 3.13, *p* = 0.001, *I*^2^ = 98.1%) [ESM Fig. S1]. The magnitude and significance of this effect was stable during one-out (%_Δ_EP range =  − 7.91 to − 7.28% and 95% CIs did not include zero) and sensitivity analyses (ESM Table S7).

Subgroup analyses demonstrated that exercise performance was negatively affected by sleep deprivation, sleep restriction and late restriction, but not early restriction (Table 2). Results indicated that sleep loss had a consistent negative influence on performance when tasks were performed in both the AM and PM; however, the magnitude of the effect was larger for PM (Table 3).

**Table 3:** Meta-analysis results for the effect of sleep loss on exercise tasks performed in the AM or PM

Exercise category	Outcomes, *n*	Exercise performance percentage change		Heterogeneity	
		Mean (95% CI)	*p *value	*I*^2^ value	*p* value
*All categories*					
*AM vs. PM*					
Overall (exercise AM)	115	− 5.42 (− 9.66 to − 1.17)	0.013	93.5	< 0.001
Overall (exercise PM)	106	− 8.31 (− 13.2 to − 3.37)	0.001	98.9	< 0.001
*Sleep-loss condition*					
Deprivation (exercise AM)	59	− 3.48 (− 5.89 to − 1.08)	0.005	94.2	< 0.001
Deprivation (exercise PM)	35	− 6.85 (− 11.3 to − 2.39)	0.004	97.5	< 0.001
Restriction (exercise AM)	56	− 5.96 (− 11.5 to − 0.43)	0.035	90.7	< 0.001
Restriction (exercise PM)	71	− 9.50 (− 14.9 to − 4.12)	0.001	99.0	< 0.001
Early restriction (exercise AM)	27	− 1.55 (− 4.66 to 1.56)	0.315	55.1	0.437
Early restriction (exercise PM)	35	− 6.23 (− 13.9 to 1.44)	0.108	94.7	< 0.001
Late restriction (exercise AM)	23	− 2.48 (− 4.36 to − 0.60)	0.012	46.1	0.048
Late restriction (exercise PM)	34	− 9.67 (− 13.1 to − 6.24)	< 0.001	99.1	< 0.001
*Anaerobic power*					
*AM vs. PM*					
Overall (exercise AM)	27	− 4.58 (− 9.14 to − 0.24)	0.049	97.0	< 0.001
Overall (exercise PM)	30	− 7.37 (− 10.3 to − 4.40)	< 0.001	97.8	< 0.001
*Sleep-loss condition*					
Deprivation (exercise AM)	14	− 6.38 (− 14.5 to 1.73)	0.113	98.5	< 0.001
Deprivation (exercise PM)	11	− 5.49 (− 7.93 to − 3.04)	< 0.001	93.5	< 0.001
Restriction (exercise AM)	13	− 2.77 (− 5.77 to 0.23)	0.067	72.3	0.010
Restriction (exercise PM)	19	− 8.35 (− 13.1 to − 3.56)	0.002	96.0	< 0.001
Early restriction (exercise AM)	5	0.25 (− 2.55 to 3.05)	0.817	< 0.01	0.988
Early restriction (exercise PM)	6	− 1.10 (− 3.42 to 1.23)	0.280	0.05	0.391
Late restriction (exercise AM)	8	− 3.46 (− 7.22 to 0.31)	0.067	78.1	0.002
Late restriction (exercise PM)	13	− 10.1 (− 14.9 to − 5.13)	0.001	96.3	< 0.001
*Speed/power endurance*					
*AM vs. PM*					
Overall (exercise AM)	14	0.11 (− 0.94 to 1.16)	0.823	< 0.001	0.721
Overall (exercise PM)	15	− 6.78 (− 10.8 to − 2.80)	0.003	98.5	< 0.001
*Sleep-loss condition*					
Deprivation (exercise AM)	5^a^	0.90 (− 0.89 to 2.69)	0.323	14.0	0.203
Deprivation (exercise PM)	5^a^	− 7.11 (− 14.4 to 0.20)	0.057	93.9	< 0.001
Restriction (exercise AM)	9	− 0.36 (− 1.84 to 1.12)	0.588	< 0.001	0.964
Restriction (exercise PM)	10	− 5.58 (− 10.4 to − 0.76)	0.028	98.8	< 0.001
Early restriction (exercise AM)	4^a^	0.62 (− 1.55 to 2.79)	0.575	0.00	0.902
Early restriction (exercise PM)	3^a^	0.43 (− 3.51 to 4.37)	0.832	68.3	0.056
Late restriction (exercise AM)	5^a^	− 0.86 (− 2.40 to 0.68)	0.275	0.00	0.954
Late restriction (exercise PM)	7^a^	− 7.17 (− 10.7 to − 3.66)	< 0.001	98.5	< 0.001
*HIIE*					
AM vs. PM					
Overall (exercise AM)	11	− 1.51 (− 10.4 to 7.42)	0.714	97.7	< 0.001
Overall (exercise PM)	16	− 8.34 (− 12.2 to − 4.47)	0.001	98.5	< 0.001
*Sleep-loss condition*					
Deprivation (exercise AM)	5^a^	− 2.06 (− 14.1 to 10.0)	0.737	99.3	< 0.001
Deprivation (exercise PM)	4^a^	− 4.13 (− 6.50 to − 1.76)	< 0.001	81.2	0.013
Restriction (exercise AM)	6	− 3.39 (− 13.3 to 6.55)	0.421	66.5	0.074
Restriction (exercise PM)	12	− 10.2 (− 15.4 to − 4.97)	0.001	99.0	< 0.001
Early restriction (exercise AM)	5	− 1.10 (− 10.7 to 8.53)	0.767	57.2	0.176
Early restriction (exercise PM)	3^a^	− 4.79 (− 13.6 to 3.98)	0.284	83.6	< 0.001
Late restriction (exercise AM)	1				
Late restriction (exercise PM)	9^a^	− 11.5 (− 16.7 to − 6.24)	< 0.001	99.4	< 0.001
*Strength*					
*AM vs. PM*					
Overall (exercise AM)	39	− 1.78 (− 3.22 to − 0.33)	0.017	17.6	0.570
Overall (exercise PM)	26	− 4.58 (− 7.59 to − 1.58)	0.004	79.5	< 0.001
*Sleep-loss condition*					
Deprivation (exercise AM)	21	− 2.43 (− 4.47 to − 0.38)	0.022	31.8	0.115
Deprivation (exercise PM)	8	− 3.79 (− 7.27 to − 0.32)	0.037	71.5	< 0.001
Restriction (exercise AM)	18	− 0.43 (− 2.41 to 1.54)	0.650	< 0.001	0.999
Restriction (exercise PM)	18	− 5.20 (− 11.0 to 0.59)	0.075	82.3	< 0.001
Early restriction (exercise AM)	12	− 0.55 (− 3.21 to 2.11)	0.659	< 0.001	0.985
Early restriction (exercise PM)	14	− 1.51 (− 4.41 to 1.39)	0.281	27.1	0.631
Late restriction (exercise AM)	6	− 0.26 (− 4.06 to 3.54)	0.867	< 0.001	0.989
Late restriction (exercise PM)	4	− 10.5 (− 20.6 to − 0.39)	0.046	84.0	< 0.001
*Endurance*					
*AM vs. PM*					
Overall (exercise AM)	12	− 6.50 (− 11.1 to − 1.86)	0.010	88.6	< 0.001
Overall (exercise PM)	9	− 3.56 (− 4.67 to − 2.45)	< 0.001	< 0.001	0.976
*Sleep-loss condition*					
Deprivation (exercise AM)	9^a^	− 7.83 (− 12.9 to − 2.72)	0.003	88.5	< 0.001
Deprivation (exercise PM)	4^a^	− 3.45 (− 4.48 to − 2.42)	< 0.001	0.00	0.905
Restriction (exercise AM)	3^a^	− 2.67 (− 4.62 to − 0.73)	0.007	0.00	0.794
Restriction (exercise PM)	5	− 4.11 (− 7.40 to − 0.82)	0.026	< 0.001	0.855
Early restriction (exercise AM)	0				
Early restriction (exercise PM)	2^a^	− 5.28 (− 9.17 to − 1.39)	0.008	0.00	0.798
Late restriction (exercise AM)	2^a^	− 2.92 (− 6.83 to 0.98)	0.143	0.00	0.507
Late restriction (exercise PM)	0				
*Strength-endurance*					
*AM vs. PM*					
Overall (exercise AM)	8	− 11.2 (− 23.3 to 0.85)	0.064	87.0	< 0.001
Overall (exercise PM)	1				
*Sleep-loss condition*					
Deprivation (exercise AM)	5	− 7.06 (-22.9 to 8.82)	0.285	60.5	0.172
Deprivation (exercise PM)	1				
Restriction (exercise AM)	3	− 18.3 (− 35.6 to − 0.96)	0.045	88.3	0.001
Restriction (exercise PM)	0				
*Skill*					
*AM vs. PM*					
Overall (exercise AM)	4	− 14.2 (− 26.7 to − 1.68)	0.037	87.0	< 0.001
Overall (exercise PM)	9	− 22.9 (− 29.7 to − 16.0)	< 0.001	93.8	< 0.001
*Sleep-loss condition*					
Deprivation (exercise AM)	0				
Deprivation (exercise PM)	2^a^	− 20.9 (− 23.6 to − 18.2)	< 0.001	0.00	0.342
Restriction (exercise AM)	4	− 14.2 (− 26.7 to − 1.67)	0.037	87.0	< 0.001
Restriction (exercise PM)	7	− 23.7 (− 34.0 to − 13.4)	0.001	95.5	< 0.001
Early restriction (exercise AM)	1				
Early restriction (exercise PM)	7	− 23.7 (− 34.0 to − 13.4)	0.001	95.5	< 0.001
Late restriction (exercise AM)	1				
Late restriction (exercise PM)	0				

A negative effect estimate indicates a decrease in performance under the intervention condition (‘sleep loss’)

Deprivation: participants did not sleep for an extended period of time (i.e., whole night); restriction: total sleep time ≤ 6 h in any 24 h period – this category is a combination of early restriction, late restriction, fragmented sleep and sleep restriction protocols not specified; early restriction: participants delayed sleep (i.e., went to sleep at a later time); late restriction; participants awakened earlier than normal

*HIIE* high-intensity interval exercise, *AM* ante meridiem, *PM* post meridiem, *CI* confidence interval

^a^All outcomes were from independent studies and the meta-analysis was run without dependency levels (i.e., simple meta-analysis)

**Fig. 3:**
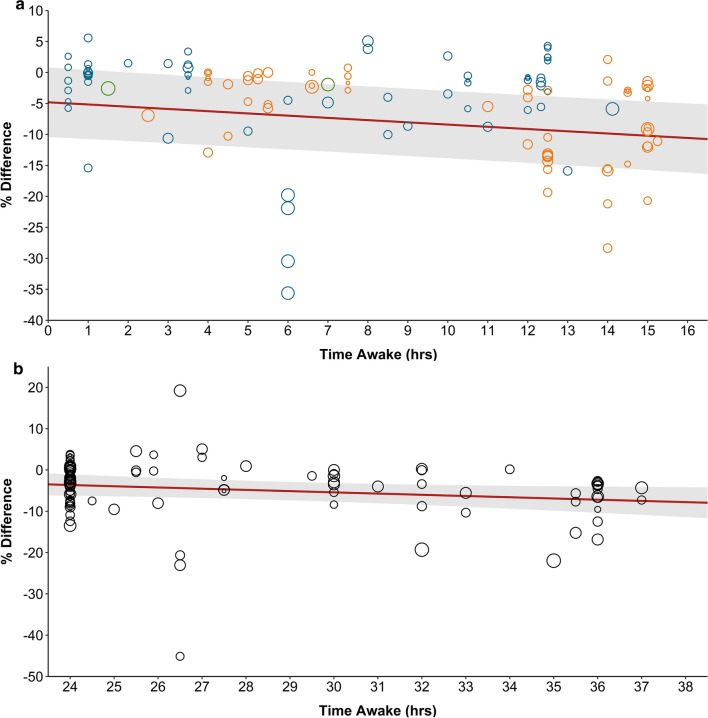
Relationship between time awake and the mean percentage change (95% CIs shown by the grey shaded area) in exercise performance for all tasks (combined exercise categories). Circle diameter corresponds to the weight of the outcome measure from each trial. **a** Sleep restriction (*n* = 121): mean %_Δ_ =  − 0.36, 95% CI − 0.52 to − 0.19; *p* < 0.001. Green circles represent ‘sleep restriction’ (not further defined); blue circles represent ‘early restriction’; and orange circles represent ‘late restriction’. **b** Sleep deprivation (*n* = 97): mean %_Δ_ =  − 0.30, 95% CI − 0.59 to 0.01; *p* = 0.051. Deprivation: participants did not sleep for an extended period of time (i.e., whole night); early restriction: participants delayed sleep (i.e., went to sleep at a later time); late restriction: participants awakened earlier than normal. *CIs* confidence intervals

Meta-regression analyses (Fig. 3 and Table 4) identified significant relationships between time awake prior to completing the exercise task and %_Δ_EP for sleep restriction (mean %_Δ_ =  − 0.36, 95% CI − 0.52 to − 0.19, *p* < 0.001), and late restriction (mean %_Δ_ =  − 0.55, 95% CI − 0.82 to − 0.28, *p* < 0.001), but not sleep deprivation (mean %_Δ_ =  − 0.30, 95% CI − 0.59 to 0.01, *p* = 0.051), early restriction (mean %_Δ_ =  − 0.10, 95% CI − 0.27 to 0.09, *p* = 0.323) or when all sleep protocols were combined (mean %_Δ_ =  − 0.09, 95% CI − 0.19 to 0.01, *p* = 0.095). However, it is important to note that for sleep deprivation, an outcome measure from Arazi et al. [85] was identified as an influential outlier (based on Cook’s distance). When performing one-out analysis, removal of this outcome measure yielded a significant result (mean %_Δ_ =  − 0.27, 95% CI − 0.48 to − 0.05, *p* = 0.015).

**Table 4:** Meta-regression relationship between time awake and percentage change in exercise performance

Exercise category	Sleep-loss condition	Outcomes, *n*	Exercise performance percentage change	
			Coefficient (95% CI)	*P* value
All categories	Overall	218	–0.09 (− 0.19 to 0.01)	0.095
	Deprivation	97	− 0.30 (− 0.59 to 0.01)	0.051
	Restriction	121	− 0.36 (− 0.52 to − 0.19)	< 0.001
	*Early restriction*	62	− 0.10 (− 0.27 to 0.09)	0.323
	*Late restriction*	57	− 0.55 (− 0.82 to − 0.28)	< 0.001
Anaerobic power	Overall	57	− 0.20 (− 0.37 to − 0.04)	0.016
	Deprivation	25	− 0.19 (− 0.49 to 0.12)	0.213
	Restriction	32	− 0.47 (− 0.79 to − 0.14)	0.007
	*Early restriction*	11	0.16 (− 0.47 to 0.15)	0.266
	*Late restriction*	21	− 0.56 (− 1.09 to − 0.02)	0.043
Speed/power endurance	Overall	31	− 0.19 (− 0.39 to 0.01)	0.063
	Deprivation	12	− 1.00 (− 1.80 to − 0.21)	0.018
	Restriction	19	− 0.46 (− 0.71 to − 0.22)	< 0.001
	*Early restriction*	7	− 0.18 (− 0.62 to 0.26)	0.344
	*Late restriction*	12	− 0.57 (− 0.98 to − 0.15)	0.012
HIIE	Overall	27	− 0.05 (− 0.21 to 0.11)	0.532
	Deprivation	9	− 0.05 (− 0.26 to 0.16)	0.605
	Restriction	18	− 0.79 (− 1.59 to 0.02)	0.056
	*Early restriction*	8	− 0.41 (− 2.14 to 1.32)	0.586
	*Late restriction*	10	0.59 (− 1.56 to 2.74)	0.546
Strength	Overall	65	− 0.08 (− 0.23 to 0.07)	0.315
	Deprivation	29	− 0.11 (− 0.46 to 0.25)	0.538
	Restriction	36	− 0.23 (− 0.52 to 0.05)	0.108
	*Early restriction*	26	− 0.04 (− 0.33 to 0.25)	0.792
	*Late restriction*	10	− 1.07 (− 2.05 to − 0.10)	0.035
Endurance	Overall	21	− 0.12 (− 0.39 to 0.14)	0.345
	Deprivation	14	0.77 (− 0.55 to 2.09)	0.253
	Restriction	7	− 0.24 (− 0.77 to 0.29)	0.290
	*Early restriction*	2		
	*Late restriction*	3	− 0.46 (− 1.97 to 1.05)	0.548
Skill	Overall	11	− 0.03 (− 0.57 to 0.51)	0.896
	Sleep deprivation	2		
	Restriction	9	− 0.15 (− 1.39 to 1.09)	0.782
	*Early restriction*	8	− 0.02 (− 1.28 to 1.25)	0.975
	*Late restriction*	1		

Deprivation: participants did not sleep for an extended period of time (i.e., whole night); restriction: total sleep time ≤ 6 h in any 24 h period—this category is a combination of early restriction, late restriction, fragmented sleep and sleep restriction protocols not specified; early restriction: participants delayed sleep (i.e., went to sleep at a later time); late restriction; participants awakened earlier than normal

*HIIE* high-intensity interval exercise, *CI* confidence interval

### Anaerobic Power

Thirty-two studies (*n* = 368; 92% male), providing 58 outcome measures, were included in this analysis. The overall weighted mean effect estimate (Table 2) indicated a negative influence of sleep loss on anaerobic power (mean %_Δ_ =  − 6.26%, 95% CI − 9.10 to − 3.41, *p* < 0.001, *I*^2^  = 98.1%) [ESM Fig. S2]. The magnitude and statistical significance of the effect were stable during one-out (mean %_Δ_ range =  − 6.59 to − 5.24% and 95% CIs did not include zero) and sensitivity analyses (ESM Table S8).

Subgroup analyses showed that anaerobic power was negatively affected by sleep deprivation, sleep restriction and late restriction, but not early restriction (Table 2). Results were consistent for anaerobic power tasks performed in the PM, while performance in the AM tended to be unaffected, with the exception of analysis for all sleep-loss protocols combined (Table 3).

Meta-regression analyses identified significant relationships between time awake prior to completing the exercise task and %_Δ_ in anaerobic power when all sleep-loss protocols were included (mean %_Δ_ =  − 0.20, 95% CI − 0.37 to − 0.04, *p* = 0.016), when both sleep restriction protocols (i.e., early and late restriction) were combined (mean %_Δ_ =  − 0.47, 95% CI − 0.79 to − 0.14, *p* = 0.007) and late restriction (mean %_Δ_ =  − 0.56, 95% CI − 1.09 to − 0.02, *p* = 0.043). No significant relationships were detected for the other sleep-loss protocols (Table 4).

### Speed/Power Endurance

Twenty studies (*n* = 261; 97% male), providing 32 outcome measures, were included in this analysis. The overall weighted mean effect estimate (Table 2) indicated a negative influence of sleep loss on speed/power endurance (mean %_Δ_ =  − 2.90%, 95% CI − 4.97 to − 0.82, *p* = 0.008, *I*^2^ = 96.3%) [ESM Fig. S3). The magnitude and significance of the effect were stable during one-out (mean %_Δ_ range =  − 3.72 to − 2.43% and 95% CIs did not include zero) and sensitivity analyses (ESM Table S9).

Subgroup analyses showed that speed/power endurance was negatively affected by sleep restriction and late-restriction protocols, but not sleep deprivation or early restriction (Table 2). However, when the trial from Abedelmalek et al. [62] was removed (during one-out analyses), the effect on sleep restriction was no longer significant (ESM Table S9). Results indicated that sleep loss had a consistent negative influence on speed/power endurance when analysis was isolated to tasks performed in the PM, while tasks performed in the AM were unaffected.

Meta-regression analyses (Table 4) detected significant relationships between time awake prior to completing the exercise task and the %_Δ_ in speed/power endurance following sleep deprivation (mean %_Δ_ =  − 1.00, 95% CI − 1.80 to − 0.21, *p* = 0.018), sleep restriction (mean %_Δ_ =  − 0.46, 95% CI − 0.71 to − 0.22, *p* < 0.001) and late restriction (mean %_Δ_ =  − 0.57, 95% CI − 0.98 to − 0.15, *p* = 0.012). No significant relationships were detected for the other sleep-loss protocols (Table 4).

### High-Intensity Interval Exercise

Eighteen studies (*n* = 207; 88% male), providing 27 outcome measures, were included in this analysis. The overall weighted mean effect estimate (Table 2) indicated a negative influence of sleep loss on HIIE (mean %_Δ_ =  − 6.15%, 95% CI − 10.5 to − 1.77, *p* = 0.008, *I*^2^ = 98.9%) [ESM Fig. S4]. The magnitude and statistical significance of the effect were stable during one-out (mean %_Δ_ range =  − 7.54 to − 5.57% and 95% CIs did not include zero) and sensitivity analyses (ESM Table S10).

Subgroup analyses indicated that HIIE performance was negatively affected following sleep restriction and late restriction, but not sleep deprivation or early restriction (Table 2). However, when the study by Arazi et al. [85] was removed (during one-out analyses) the effect for sleep deprivation was significant (mean %_Δ_ =  − 4.21%, 95% CI − 6.45 to − 1.97, *p* = 0.003). Results indicated that sleep loss had a consistent negative influence on HIIE when analysis was conducted on tasks performed in the PM (except for early restriction), while tasks performed in the AM were unaffected.

No significant relationships between time awake prior to completing the task and %_Δ_ in HIIE were identified for meta-regression analysis with any of the sleep-loss protocols (Table 4).

### Strength

Twenty-five studies (*n* = 289; 74% male), providing 66 outcome measures, were included in this analysis. The overall weighted mean effect estimate (Table 2) indicated a negative influence of sleep loss on strength (mean %_Δ_ =  − 2.85%, 95% CI − 4.47 to − 1.23, *p* < 0.001, *I*^2^ = 62.2%) [ESM Fig. S5]. The magnitude and statistical significance of the effect were stable during one-out (mean %_Δ_ range =  − 3.20 to − 2.27% and 95% CIs did not include zero) and sensitivity analyses (ESM Table S11).

In subgroup analyses, a significant negative influence was only observed for sleep deprivation (mean %_Δ_ =  − 3.00%, 95% CI − 4.52 to − 1.48, *p* < 0.001, I^2^ = 49.2%) (Table 2). Results indicated that sleep loss had a consistent negative influence on strength when analysis was isolated to tasks performed in the PM, while tasks performed in the AM were generally unaffected (Table 3).

The effects of sleep loss were also conditional on body–limb categorisation, with tasks involving lower-body strength demonstrating a negative influence on performance, while tasks requiring upper-body strength were unaffected (Table 5; limb strength AM vs. PM comparison in ESM Table S12).

**Table 5:** Influence of sleep loss on body–limb strength

Exercise category	Outcomes, *n*	Exercise performance percentage change		Heterogeneity	
		Mean (95% CI)	*p* value	*I*^2^ value	*p *value
*Upper- vs. lower-body strength*					
Overall upper body	18	− 1.63 (− 3.30 to 0.04)	0.056	32.7	0.069
Overall lower body	46	− 3.42 (− 5.54 to − 1.31)	0.002	65.6	< 0.001
*Sleep-loss condition*					
Upper body					
Deprivation	6	− 3.18 (− 9.13 to 2.77)	0.228	58.9	0.104
Restriction	12	− 0.73 (− 2.67 to 1.22)	0.428	11.5	0.186
Early restriction	6	− 1.21 (− 3.71 to 1.29)	0.268	< 0.001	0.752
Late restriction	6	− 1.13 (− 6.07 to 3.81)	0.583	61.6	0.035
*Lower-body*					
Deprivation	21	− 3.25 (− 5.09 to − 1.41)	0.002	54.7	< 0.001
Restriction	25	− 4.50 (− 10.2 to 1.17)	0.114	72.8	< 0.001
Early restriction	20	− 1.36 (− 3.81 to 1.09)	0.259	6.97	0.892
Late restriction	5	− 8.26 (− 20.4 to 3.90)	0.132	81.7	< 0.001

Deprivation: participants did not sleep for an extended period of time (i.e., whole night); restriction: total sleep time ≤ 6 h in any 24 h period—this category is a combination of early restriction, late restriction, fragmented sleep and sleep restriction protocols not specified; early restriction: participants delayed sleep (i.e., went to sleep at a later time); late restriction; participants awakened earlier than normal

*CI* confidence interval

Meta-regression analyses (Table 4) detected a significant relationship between time awake prior to completing the exercise task and %_Δ_ in strength, but only following late restriction (mean %_Δ_ =  − 1.07, 95% CI − 2.05 to − 0.10, *p* = 0.035). No significant relationships were detected for the other sleep-loss protocols (Table 4).

### Endurance

Twenty studies (*n* = 237; 91% male), providing 22 outcome measures, were included in this analysis. The overall weighted mean effect estimate (Table 2) indicated a negative influence of sleep loss on endurance (mean %_Δ_ =  − 5.55%, 95% CI − 8.12 to − 2.99, *p* < 0.001, *I*^2^ = 86.5%) [ESM Fig. S6]. The magnitude and statistical significance of the effect were stable during one-out (mean %_Δ_ range =  − 5.94 to − 3.72% and 95% CIs did not include zero) and sensitivity analyses (ESM Table S13).

Subgroup analyses showed that all sleep protocols were negatively affected by sleep loss (Table 2); however, there were only two outcome measures available for early restriction. Endurance performance tended to be affected (Table 3) by sleep loss, irrespective of the time of day exercise tasks were performed (AM or PM).

No significant relationships between time awake prior to completing the exercise task and the %_Δ_ in endurance performance were identified in meta-regression analyses with any of the sleep-loss protocols (Table 4).

### Strength-Endurance

Five studies (*n* = 62; 100% male), providing nine outcome measures, were included in this analysis. The overall weighted mean effect estimate (Table 2) indicated a negative influence of sleep loss on strength-endurance (mean %_Δ_ =  − 9.85%, 95% CI − 19.6 to − 0.13, *p* = 0.048, *I*^2^ = 85.4%) [ESM Fig. S7]. However, the magnitude and statistical significance of the effect was unstable during one-out analyses (mean %_Δ_ range =  − 11.2 to − 8.71% and 95% CIs did not include zero except when outcome measures from six trials were sequentially removed [60, 75, 84, 86]). Findings were comparable with alternative correlation coefficients (ESM Table S14).

Subgroup analyses showed that strength-endurance was negatively affected by sleep restriction, but not sleep deprivation (Table 2). Note, however, that the three outcome measures analysed for sleep restriction were derived from one study [84]. There were no outcome measures to conduct analysis for either early- or late-restriction sleep protocols.

There were insufficient outcome measures to conduct meta-regression analyses on this exercise category.

### Skill

Nine studies (*n* = 146; 80% male), providing 13 outcome measures, were included in this analysis. The overall weighted mean effect estimate (Table 2) indicated a negative influence of sleep loss on skill (mean %_Δ_ =  − 20.9%, 95% CI − 27.0 to − 14.9, *p* < 0.001, *I*^2^ = 94.1%) [ESM Fig. S8). The magnitude and statistical significance of the effect were stable during one-out (mean %_Δ_ range =  − 22.6 to − 19.2% and 95% CIs did not include zero) and sensitivity analyses (ESM Table S15).

Subgroup analyses showed that skill performance was negatively affected irrespective of the sleep-loss protocol (Table 2) or whether tasks were performed in the AM or PM. Note, there were insufficient outcome measures to conduct meta-analysis for late restriction.

No significant relationships between time awake prior to completing the task and %_Δ_ in skill performance were identified in meta-regression analyses for any of the sleep-loss protocols (Table 4).

## Discussion

The present systematic review and meta-analysis aimed to characterise the effects of acute sleep loss on exercise performance. We explored the influence of various contextual factors, including the type of exercise task(s) performed, pattern of sleep loss incurred before exercise, time of day (AM or PM) the exercise task was performed, and length of time awake prior to undertaking the exercise task. Overall, our results indicate that acute sleep loss negatively impacts next-day exercise performance; however, the magnitude of the impact depends on the type of exercise performed, as well as which sleep-loss pattern precedes exercise. Total sleep loss (deprivation) and late restriction (early awakening) appear to have a larger effect on exercise performance than early restriction (delayed sleep). Results also suggest that exercise performed in the PM is more likely to be affected by sleep loss than exercise performed in the AM, and that the length of time awake prior to exercise is an influential factor.

### Influence of Acute Sleep Loss on Exercise Performance

When all sleep-loss protocols (i.e., deprivation, restriction, early restriction, late restriction) were consolidated, our meta-analyses showed that acute sleep loss has a negative impact on all exercise categories (Table 2).

Tasks requiring a skill component appear to be particularly sensitive to the effects of sleep loss (mean %_Δ_ =  − 20.9, 95% CI − 27.0 to − 14.9) (Table 2). This may be attributed to the higher cognitive demand required to undertake skill performance tasks [133]. Sleep loss has been shown to alter discrete cognitive functions, including reaction time [99, 134], alertness [58], attention [134], memory [135], decision making [136, 137] and learning [138]. Thus, physical tasks that are also cognitively demanding are likely to be most affected by acute sleep loss.

A number of investigations have attempted to identify mechanisms explaining the relationship between sleep loss and impaired exercise performance. Studies have explored changes to cardiorespiratory variables (e.g., $\dot{V}$O_2peak_ [49, 50, 120, 126, 132], ventilation [41, 49, 93, 110, 120, 126, 132], heart rate [41, 49, 50, 52, 91, 110, 120, 124, 126, 128, 132, 139], blood pressure [50]); perceived effort (measured via rating of perceived exertion) [41, 43, 44, 51, 52, 56, 57, 75, 86, 89–92, 95, 100, 110, 124, 132, 139]; muscle glycogen [91]; lactate [49, 67, 77, 91, 93, 95, 98, 124, 128, 139]; catecholamines [67, 121, 126]; hormones (cortisol [43, 55, 63, 67, 75, 84, 127], testosterone [63, 75, 84, 127], growth hormone [67], prolactin [67], melatonin [55], hepcidin [54], insulin [61]); body temperature (oral temperature [43, 45, 78, 79, 81, 90, 94, 95, 116] and core temperature [53, 80, 91, 110]); immune function [44, 50, 54, 62, 127]; and neural drive [60, 76, 86, 92]. However, it was not the intention of the present study to explore these mechanisms; rather our aim was to quantify the magnitude of effects that acute sleep loss has on exercise performance. As such, the reader is referred to the comprehensive review by Fullagar et al. [36] on sleep and athletic performance for further details on the physiological responses associated with sleep loss.

### Pattern of Sleep Loss

Another important finding in our study was the difference in the magnitude of change in performance when different types of sleep loss were analysed (i.e., deprivation, restriction, early and late restriction). We observed no change in exercise performance when early restriction sleep-loss protocols were isolated, except for skill and endurance tasks. For these two categories, the timing of the task should be considered (the influence of time of day is discussed in more detail in Sect. 4.3). For skill tasks, seven of eight outcome measures were performed in the PM. There were also only two outcome measures for endurance tasks and both were performed in the PM. Thus, individuals performing all other tasks (anaerobic power, speed/power endurance, HIIE, strength, and strength-endurance) appear able to maintain their performance under conditions of early sleep restriction.

In contrast to results identified with early-restriction protocols, the detrimental effects observed with sleep deprivation and late-restriction protocols appear to be more consistent and similar in magnitude. This may be a result of greater changes to one or more of the aforementioned mechanisms (highlighted in Sect. 4.1) underpinning exercise performance. Indeed, it may provide more opportunity to accrue particular aspects (e.g., fatigue [140]) when participants are kept awake or awoken early from sleep (i.e., in late-restriction protocols) until when the performance task is completed [36]. Given these results, when sleep loss is unavoidable and individuals have some level of control over timing, early restriction would appear preferable to late restriction. From a practical perspective, should an athlete need to travel, it would be reasonable for a health professional to recommend that it is better to do so the night before and sleep locally (even if that results in delayed sleep onset), rather than wake early for travel.

Our meta-regression analyses identified a significant negative relationship between the time awake prior to completing the exercise task and the %_Δ_ EP for both sleep deprivation and late-restriction protocols (note, our interpretation of results for sleep deprivation is based on the removal of the influential outlier [85]). Specifically, we found that on average, exercise performance declined by ~ 0.4% per hour following sleep loss (note, this result is not inflated by the skill category because 8/13 of these tasks were performed following an early-restriction protocol). For example, if an individual rises early (e.g., ~ 0300 h) and performs a task 12 h later (~ 1500 h), then a ~ 5% decrease in performance may be anticipated. Overall, these results suggest that if exercise is to be performed after a period of sleep loss, it should be done as soon as practically possible.

### Influence of Time of Day Exercise is Performed

Results of the current study suggest that exercise performed in the PM is likely to be more adversely affected by sleep loss than exercise performed in the AM (Table 3). The influence time of day has on exercise performance (without sleep loss) is well documented [70]. Evidence suggests that exercise performance may improve throughout the day for a number of tasks (skill [141–143], strength [144, 145], anaerobic power [81, 146–148], swimming [149, 150], and endurance tasks [151–153]), and this may be a consequence of physiological changes that occur with shifts in the circadian cycle (e.g., core temperature) [70, 154]. On this basis, one might anticipate that the negative impact of sleep loss may be offset when tasks are performed in the afternoon or evening. However, our results suggest that performing exercise in the PM (hence inducing a greater period between the start of sleep loss and the commencement of the task) appears to be a more significant moderator of exercise performance than changes associated with normal circadian rhythms. Therefore, in the setting of acute sleep loss, exercise should be scheduled to be performed soon after waking, before performance is potentially compromised by training in the PM.

### Limitations and Future Direction

At present, we are unable to explore the relationship between sleep quality and next-day exercise performance. The majority (~ 98%) of included outcomes were obtained from studies that only assessed sleep ‘quantity’ (i.e., time spent asleep—more often reflected by ‘time in bed’). Polysomnography (PSG) is considered the ‘gold standard’ sleep assessment technique, and can provide important information on sleep architecture (e.g., time spent in non-rapid eye movement and rapid eye movement sleep stages [155]). As such, future studies should employ PSG for monitoring sleep, which will permit further exploration of the relationship between sleep quality and next-day exercise performance.

In the present review, we were unable to determine the influence of fragmented sleep (i.e., one or more nocturnal awakenings [48]) on next-day performance. To our knowledge only one investigation has been conducted on fragmented sleep [49], despite reports suggesting this is something athletes often experience [20, 24]; thus, further research targeting this specific sleep pattern is warranted. We also dichotomised time of day for task completion as AM or PM, which prevented exploration of effects at more specific times (e.g., early- vs. mid-morning and afternoon vs. evening). Furthermore, only 8/227 outcomes were measured later than 1800 h [45, 50, 86, 97, 121, 128, 132]. Given that many sporting events are carried out during the evening, future research should investigate the influence of sleep loss on tasks performed after 1800 h.

The influence of sleep loss on performance in the present study was based on discrete task categories. However, in reality, many sports require using concurrent physical/cognitive attributes (e.g., soccer, football), where certain skill activities (e.g., shot at goal/target) are frequently performed following short maximal sprint efforts or brief spurts of maximal effort interspersed with short recovery periods (e.g., HIIE). As such, future studies should explore the influence of sleep loss on performance tasks that involve a combination of physical/cognitive attributes to enhance translation and ecological validity with respect to team sports.

Finally, we were unable to explore the influence of certain factors in our analyses, often because insufficient data were available. For example, only a small number of female participants were included in studies (~ 11%), precluding exploration of sex as a variable. Furthermore, we could not investigate the impact of consecutive days/nights of sleep loss on exercise performance, nor explore the influence of participant training status. These present as opportunities for future research to further our understanding of potential factors that may influence the effects of sleep loss on exercise performance.

## Conclusion

Acute sleep loss appears to have a negative impact on next-day exercise performance. The magnitude of the effect may be greater when individuals experience either sleep deprivation or late restriction, and when performance tasks are conducted in the PM. Individuals can anticipate a ~ 0.4% decline in performance for every hour spent awake following acute sleep loss. Thus, incorporating lifestyle behaviours/strategies that limit the likelihood of experiencing sleep loss must be emphasised. However, if acute sleep loss is anticipated and unavoidable, individuals should, where possible, endeavour to mimic early-restriction sleep patterns rather than deprivation or late restriction, and prioritise exercise to the morning in an effort to maintain performance.

## Supplementary Information

Below is the link to the electronic supplementary material.

Supplementary file1 (PDF 140 KB)

Supplementary file2 (XLSX 44686 KB)

Supplementary file3 (DOCX 3708 KB)

## Footnotes

1 Goh et al. [55] undertook two additional hand-grip strength measurements during the sleep deprivation arm of the study that were not matched in the control arm; this was deemed likely to not have an influence, and was thus included in the analysis.
