# Supplementary material for: Effects of Acute Sleep Loss on Physical Performance: A Systematic and Meta-Analytical Review
Source: Sports Med. 2022 Jun 16;52(11):2669–90. doi: 10.1007/s40279-022-01706-y (PMC9584849; doi:10.1007/s40279-022-01706-y)
Supplement: Supplementary file 1 — Supplementary file1 (PDF 140 KB) [file 40279_2022_1706_MOESM1_ESM.pdf]

```r
## Example R code for Multi-Level Meta-Analysis and Meta-Regression (including
plots) for time awake prior to exercise task (number of hours) - (Craven et al)

############################################################################

# clear workspace
rm(list=ls())

# install.packages ('metafor' and 'tidyverse')
library(metafor)
library(tidyverse)

####Meta-Analysis of Sleep Loss on Exercise Performance - All Task Categories

# read in data

data <- read.csv("All tasks_master.csv")
View(data)

options(max.print=1000000)

#All task categories meta-analysis
m1 <- rma.mv(yi, vi, random = list(~ 1 | effectID,
                                   ~ 1 | modeID,
                                   ~ 1 | studyID), tdist=TRUE, data=data,
                                   slab=data$Study)
m1

#Calculate I^2
W <- diag(1/data$vi)
X <- model.matrix(m1)
P <- W - W %*% X %*% solve(t(X) %*% W %*% X) %*% t(X) %*% W
I2 <- 100 * sum(m1$sigma2) / (sum(m1$sigma2) + (m1$k-m1$p)/sum(diag(P)))
I2

#Examine Cook's distance
x <- cooks.distance.rma.mv(m1)
rev(sort(x))
plot(x, type="o", pch=19, xlab="Observed Outcome", ylab="Cook's Distance")

##One-out analysis
#Create new data frame using with matrix for estimates when trial is removed
df <- data.frame(matrix(NA, ncol = 7, nrow = length(data$Study)))
df[, 1] <- data$Study
names(df) <- c("Study", "estimate", "se", "tval", "pval", "ci.lb", "ci.ub")
df

#Create 'for loop' to run multilevel meta-analysis leaving one trial out and
#enter coefficients into the data-frame called "df"
for(i in 1:length(data$Study)){
  df.loo <- data[-i,]
  df.sens.loo <- rma.mv(yi, vi, random = list(~ 1 | effectID,
                                              ~ 1 |
                                              modeID,

                                              ~ 1 | studyID), tdist=TRUE, data=df.loo)
  re_table <- coef(summary(df.sens.loo))
  df[i,-1] <- re_table
}

df[with(df, order(estimate)),]
## View results (effect estimates, se, pval etc. with each trial removed)
df

####################
#Sensitivity analysis with R=0.3
m1a <- rma.mv(yi1, vi1, random = list(~ 1 | effectID,
                                      ~ 1 | modeID,
                                      ~ 1 | studyID), tdist=TRUE, data=data,
                                      slab=data$Study)
m1a
```

```r
#Sensitivity analysis with R=0.8
m1b <- rma.mv(yi2, vi2, random = list(~ 1 | effectID,
                                      ~ 1 | modeID,
                                      ~ 1 | studyID), tdist=TRUE, data=data,
                                      slab=data$Study)
m1b

####################
#All task categories meta-analysis (Sleep Deprivation)
data2 <- subset.data.frame(data, Sleepprotocol=="Sleep deprivation")

m1d <- rma.mv(yi, vi, random = list(~ 1 | effectID,
                                    ~ 1 | modeID,
                                    ~ 1 | studyID), tdist=TRUE, data=data2)
m1d

#Calculate I^2
W <- diag(1/data2$vi)
X <- model.matrix(m1d)
P <- W - W %*% X %*% solve(t(X) %*% W %*% X) %*% t(X) %*% W
I2 <- 100 * sum(m1d$sigma2) / (sum(m1d$sigma2) + (m1d$k-m1d$p)/sum(diag(P)))
I2

##One-out analysis
#Create new data frame using with matrix for estimates when trial is removed
df2 <- data.frame(matrix(NA, ncol = 7, nrow = length(data2$Study)))
df2[, 1] <- data2$Study
names(df2) <- c("Study", "estimate", "se", "tval", "pval", "ci.lb", "ci.ub")
df2

#Create 'for loop' to run multilevel meta-analysis leaving one trial out and
#enter coefficients into the data-frame called "df"
for(i in 1:length(data2$Study)){
  df.loo2 <- data2[-i,]
  df.sens.loo2 <- rma.mv(yi, vi, random = list(~ 1 | effectID,
                                               ~ 1 | modeID,
                                               ~ 1 | studyID), tdist=TRUE,
                                               data=df.loo2)
  re_table2 <- coef(summary(df.sens.loo2))
  df2[i,-1] <- re_table2
}

df2[with(df2, order(estimate)),]
## View results (effect estimates, se, pval etc. with each trial removed)
df2

####################
#Sensitivity analysis with R=0.3
m1e <- rma.mv(yi1, vi1, random = list(~ 1 | effectID,
                                      ~ 1 | modeID,
                                      ~ 1 | studyID), tdist=TRUE, data=data2)
m1e

#Sensitivity analysis with R=0.8
m1f <- rma.mv(yi2, vi2, random = list(~ 1 | effectID,
                                      ~ 1 | modeID,
                                      ~ 1 | studyID), tdist=TRUE, data=data2)
m1f

####################
#All task categories meta-analysis (Sleep Restriction)
data3 <- subset.data.frame(data, Sleepprotocol!="Sleep deprivation")

m1g <- rma.mv(yi, vi, random = list(~ 1 | effectID,
                                    ~ 1 | modeID,
                                    ~ 1 | studyID), tdist=TRUE, data=data3)
m1g

#Calculate I^2
W <- diag(1/data3$vi)
```

```
X <- model.matrix(m1g)
P <- W - W %*% X %*% solve(t(X) %*% W %*% X) %*% t(X) %*% W
I2 <- 100 * sum(m1g$sigma2) / (sum(m1g$sigma2) + (m1g$k-m1g$p)/sum(diag(P)))
I2

##One-out analysis
#Create new data frame using with matrix for estimates when trial is removed
df3 <- data.frame(matrix(NA, ncol = 7, nrow = length(data3$Study)))
df3[, 1] <- data3$Study
names(df3) <- c("Study", "estimate", "se", "tval", "pval", "ci.lb", "ci.ub")
df3

#Create 'for loop' to run multilevel meta-analysis leaving one trial out and
#enter coefficients into the data-frame called "df"
for(i in 1:length(data3$Study)){
  df.loo3 <- data3[-i,]
  df.sens.loo3 <- rma.mv(yi, vi, random = list(~ 1 | effectID,
                                                ~ 1 | modeID,
                                                ~ 1 | studyID), tdist=TRUE,
                         control=list(optimizer="optim", optmethod="BFGS"),
                         data=df.loo3)
  re_table3 <- coef(summary(df.sens.loo3))
  df3[i,-1] <- re_table3
}

df3[with(df3, order(estimate)),]
## View results (effect estimates, se, pval etc. with each trial removed)
df3

####################
#Sensitivity analysis with R=0.3
m1h <- rma.mv(yi1, vi1, random = list(~ 1 | effectID,
                                       ~ 1 | modeID,
                                       ~ 1 | studyID), tdist=TRUE, data=data3)
m1h

#Sensitivity analysis with R=0.8
m1i <- rma.mv(yi2, vi2, random = list(~ 1 | effectID,
                                       ~ 1 | modeID,
                                       ~ 1 | studyID), tdist=TRUE, data=data3)
m1i

####################
#All task categories meta-analysis (Early Restriction)
data4 <- subset.data.frame(data, Sleepprotocol=="Early restriction")

m1j <- rma.mv(yi, vi, random = list(~ 1 | effectID,
                                     ~ 1 | modeID,
                                     ~ 1 | studyID), tdist=TRUE, data=data4)
m1j

#Calculate I^2
W <- diag(1/data4$vi)
X <- model.matrix(m1j)
P <- W - W %*% X %*% solve(t(X) %*% W %*% X) %*% t(X) %*% W
I2 <- 100 * sum(m1j$sigma2) / (sum(m1j$sigma2) + (m1j$k-m1j$p)/sum(diag(P)))
I2

##One-out analysis
#Create new data frame using with matrix for estimates when trial is removed
df4 <- data.frame(matrix(NA, ncol = 7, nrow = length(data4$Study)))
df4[, 1] <- data4$Study
names(df4) <- c("Study", "estimate", "se", "tval", "pval", "ci.lb", "ci.ub")
df4

#Create 'for loop' to run multilevel meta-analysis leaving one trial out and
#enter coefficients into the data-frame called "df"
for(i in 1:length(data4$Study)){
  df.loo4 <- data4[-i,]
  df.sens.loo4 <- rma.mv(yi, vi, random = list(~ 1 | effectID,
                                                ~ 1 | modeID,
```

```
                                      ~ 1 | studyID), tdist=TRUE,
                                      data=df.loo4)
  re_table4 <- coef(summary(df.sens.loo4))
  df4[i,-1] <- re_table4
}

df4[with(df4, order(estimate)),]
## View results (effect estimates, se, pval etc. with each trial removed)
df4

####################
#Sensitivity analysis with R=0.3
m1k <- rma.mv(yi1, vi1, random = list(~ 1 | effectID,
                                      ~ 1 | modeID,
                                      ~ 1 | studyID), tdist=TRUE, data=data4)
m1k

#Sensitivity analysis with R=0.8
m1l <- rma.mv(yi2, vi2, random = list(~ 1 | effectID,
                                      ~ 1 | modeID,
                                      ~ 1 | studyID), tdist=TRUE, data=data4)
m1l

####################
#All task categories meta-analysis (Late Restriction)
data5 <- subset.data.frame(data, Sleepprotocol=="Late restriction")

m1m <- rma.mv(yi, vi, random = list(~ 1 | effectID,
                                    ~ 1 | modeID,
                                    ~ 1 | studyID), tdist=TRUE, data=data5)
m1m

#Calculate I^2
W <- diag(1/data5$vi)
X <- model.matrix(m1m)
P <- W - W %*% X %*% solve(t(X) %*% W %*% X) %*% t(X) %*% W
I2 <- 100 * sum(m1m$sigma2) / (sum(m1m$sigma2) + (m1m$k-m1m$p)/sum(diag(P)))
I2

##One-out analysis
#Create new data frame using with matrix for estimates when trial is removed
df5 <- data.frame(matrix(NA, ncol = 7, nrow = length(data5$Study)))
df5[, 1] <- data5$Study
names(df5) <- c("Study", "estimate", "se", "tval", "pval", "ci.lb", "ci.ub")
df5

#Create 'for loop' to run multilevel meta-analysis leaving one trial out and
#enter coefficients into the data-frame called "df"
for(i in 1:length(data5$Study)){
  df.loo5 <- data5[-i,]
  df.sens.loo5 <- rma.mv(yi, vi, random = list(~ 1 | effectID,
                                              ~ 1 | modeID,
                                              ~ 1 | studyID), tdist=TRUE,
                                      data=df.loo5)
  re_table5 <- coef(summary(df.sens.loo5))
  df5[i,-1] <- re_table5
}

df5[with(df5, order(estimate)),]
## View results (effect estimates, se, pval etc. with each trial removed)
df5

####################
#Sensitivity analysis with R=0.3
m1n <- rma.mv(yi1, vi1, random = list(~ 1 | effectID,
                                      ~ 1 | modeID,
                                      ~ 1 | studyID), tdist=TRUE, data=data5)
m1n

#Sensitivity analysis with R=0.8
m1o <- rma.mv(yi2, vi2, random = list(~ 1 | effectID,
```

```
                                           ~ 1 | modeID,
                                           ~ 1 | studyID), tdist=TRUE, data=data5)
m1o

####################
#All task categories meta-analysis (Exercise in AM)
data6 <- subset.data.frame(data, TOTamvpm=="AM")

m1p <- rma.mv(yi, vi, random = list(~ 1 | effectID,
                                    ~ 1 | modeID,
                                    ~ 1 | studyID), tdist=TRUE, data=data6)
m1p

#Calculate I^2
W <- diag(1/data6$vi)
X <- model.matrix(m1p)
P <- W - W %*% X %*% solve(t(X) %*% W %*% X) %*% t(X) %*% W
I2 <- 100 * sum(m1p$sigma2) / (sum(m1p$sigma2) + (m1p$k-m1p$p)/sum(diag(P)))
I2

##One-out analysis
#Create new data frame using with matrix for estimates when trial is removed
df6 <- data.frame(matrix(NA, ncol = 7, nrow = length(data6$Study)))
df6[, 1] <- data6$Study
names(df6) <- c("Study", "estimate", "se", "tval", "pval", "ci.lb", "ci.ub")
df6

#Create 'for loop' to run multilevel meta-analysis leaving one trial out and
#enter coefficients into the data-frame called "df"
for(i in 1:length(data6$Study)){
  df.loo6 <- data6[-i,]
  df.sens.loo6 <- rma.mv(yi, vi, random = list(~ 1 | effectID,
                                               ~ 1 | modeID,
                                               ~ 1 | studyID), tdist=TRUE,
                                               data=df.loo6)
  re_table6 <- coef(summary(df.sens.loo6))
  df6[i,-1] <- re_table6
}

df6[with(df6, order(estimate)),]
## View results (effect estimates, se, pval etc. with each trial removed)
df6

####################
#Sensitivity analysis with R=0.3
m1q <- rma.mv(yi1, vi1, random = list(~ 1 | effectID,
                                      ~ 1 | modeID,
                                      ~ 1 | studyID), tdist=TRUE, data=data6)
m1q

#Sensitivity analysis with R=0.8
m1r <- rma.mv(yi2, vi2, random = list(~ 1 | effectID,
                                      ~ 1 | modeID,
                                      ~ 1 | studyID), tdist=TRUE, data=data6)
m1r

####################
#All task categories meta-analysis (Exercise in PM)
data7 <- subset.data.frame(data, TOTamvpm=="PM")

m1s <- rma.mv(yi, vi, random = list(~ 1 | effectID,
                                    ~ 1 | modeID,
                                    ~ 1 | studyID), tdist=TRUE, data=data7)
m1s

#Calculate I^2
W <- diag(1/data7$vi)
X <- model.matrix(m1s)
P <- W - W %*% X %*% solve(t(X) %*% W %*% X) %*% t(X) %*% W
I2 <- 100 * sum(m1s$sigma2) / (sum(m1s$sigma2) + (m1s$k-m1s$p)/sum(diag(P)))
I2
```

```
##One-out analysis
#Create new data frame using with matrix for estimates when trial is removed
df7 <- data.frame(matrix(NA, ncol = 7, nrow = length(data7$Study)))
df7[, 1] <- data7$Study
names(df7) <- c("Study", "estimate", "se", "tval", "pval", "ci.lb", "ci.ub")
df7

#Create 'for loop' to run multilevel meta-analysis leaving one trial out and
#enter coefficients into the data-frame called "df"
for(i in 1:length(data7$Study)){
  df.loo7 <- data7[-i,]
  df.sens.loo7 <- rma.mv(yi, vi, random = list(~ 1 | effectID,
                                                ~ 1 | modeID,
                                                ~ 1 | studyID), tdist=TRUE,
                                                data=df.loo7)
  re_table7 <- coef(summary(df.sens.loo7))
  df7[i,-1] <- re_table7
}

df7[with(df7, order(estimate)),]
## View results (effect estimates, se, pval etc. with each trial removed)
df7

####################
#Sensitivity analysis with R=0.3
m1t <- rma.mv(yi1, vi1, random = list(~ 1 | effectID,
                                      ~ 1 | modeID,
                                      ~ 1 | studyID), tdist=TRUE, data=data7)
m1t

#Sensitivity analysis with R=0.8
m1u <- rma.mv(yi2, vi2, random = list(~ 1 | effectID,
                                      ~ 1 | modeID,
                                      ~ 1 | studyID), tdist=TRUE, data=data7)
m1u

####################
#All task categories meta-analysis (Sleep Deprivation and Exercise in AM)
data8 <- subset.data.frame(data2, TOTamvpm=="AM")

m1v <- rma.mv(yi, vi, random = list(~ 1 | effectID,
                                    ~ 1 | modeID,
                                    ~ 1 | studyID), tdist=TRUE, data=data8)
m1v

#Calculate I^2
W <- diag(1/data8$vi)
X <- model.matrix(m1v)
P <- W - W %*% X %*% solve(t(X) %*% W %*% X) %*% t(X) %*% W
I2 <- 100 * sum(m1v$sigma2) / (sum(m1v$sigma2) + (m1v$k-m1v$p)/sum(diag(P)))
I2

##One-out analysis
#Create new data frame using with matrix for estimates when trial is removed
df8 <- data.frame(matrix(NA, ncol = 7, nrow = length(data8$Study)))
df8[, 1] <- data8$Study
names(df8) <- c("Study", "estimate", "se", "tval", "pval", "ci.lb", "ci.ub")
df8

#Create 'for loop' to run multilevel meta-analysis leaving one trial out and
#enter coefficients into the data-frame called "df"
for(i in 1:length(data8$Study)){
  df.loo8 <- data8[-i,]
  df.sens.loo8 <- rma.mv(yi, vi, random = list(~ 1 | effectID,
                                                ~ 1 | modeID,
                                                ~ 1 | studyID), tdist=TRUE,
                                                data=df.loo8)
  re_table8 <- coef(summary(df.sens.loo8))
  df8[i,-1] <- re_table8
}
```

```
df8[with(df8, order(estimate)),]
## View results (effect estimates, se, pval etc. with each trial removed)
df8

####################
#Sensitivity analysis with R=0.3
m1w <- rma.mv(yi1, vi1, random = list(~ 1 | effectID,
                                      ~ 1 | modeID,
                                      ~ 1 | studyID), tdist=TRUE, data=data8)
m1w

#Sensitivity analysis with R=0.8
m1x <- rma.mv(yi2, vi2, random = list(~ 1 | effectID,
                                      ~ 1 | modeID,
                                      ~ 1 | studyID), tdist=TRUE, data=data8)
m1x

####################
#All task categories meta-analysis (Sleep Deprivation and Exercise in PM)
data9 <- subset.data.frame(data2, TOTamvpm=="PM")
m1y <- rma.mv(yi, vi, random = list(~ 1 | effectID,
                                    ~ 1 | modeID,
                                    ~ 1 | studyID), tdist=TRUE, data=data9)
m1y

#Calculate I^2
W <- diag(1/data9$vi)
X <- model.matrix(m1y)
P <- W - W %*% X %*% solve(t(X) %*% W %*% X) %*% t(X) %*% W
I2 <- 100 * sum(m1y$sigma2) / (sum(m1y$sigma2) + (m1y$k-m1y$p)/sum(diag(P)))
I2

##One-out analysis
#Create new data frame using with matrix for estimates when trial is removed
df9 <- data.frame(matrix(NA, ncol = 7, nrow = length(data9$Study)))
df9[, 1] <- data9$Study
names(df9) <- c("Study", "estimate", "se", "tval", "pval", "ci.lb", "ci.ub")
df9

#Create 'for loop' to run multilevel meta-analysis leaving one trial out and
#enter coefficients into the data-frame called "df"
for(i in 1:length(data9$Study)){
  df.loo9 <- data9[-i,]
  df.sens.loo9 <- rma.mv(yi, vi, random = list(~ 1 | effectID,
                                               ~ 1 | modeID,
                                               ~ 1 | studyID), tdist=TRUE,
                                               data=df.loo9)
  re_table9 <- coef(summary(df.sens.loo9))
  df9[i,-1] <- re_table9
}

df9[with(df9, order(estimate)),]
## View results (effect estimates, se, pval etc. with each trial removed)
df9

####################
#Sensitivity analysis with R=0.3
m1z <- rma.mv(yi1, vi1, random = list(~ 1 | effectID,
                                      ~ 1 | modeID,
                                      ~ 1 | studyID), tdist=TRUE, data=data9)
m1z

#Sensitivity analysis with R=0.8
m1aa <- rma.mv(yi2, vi2, random = list(~ 1 | effectID,
                                       ~ 1 | modeID,
                                       ~ 1 | studyID), tdist=TRUE, data=data9)
m1aa

####################
#All task categories meta-analysis (Sleep Restriction and Exercise in AM)
```

```
data10 <- subset.data.frame(data3, TOTamvpm=="AM")

m1ab <- rma.mv(yi, vi, random = list(~ 1 | effectID,
                                      ~ 1 | modeID,
                                      ~ 1 | studyID), tdist=TRUE, data=data10)
m1ab

#Calculate I^2
W <- diag(1/data10$vi)
X <- model.matrix(m1ab)
P <- W - W %*% X %*% solve(t(X) %*% W %*% X) %*% t(X) %*% W
I2 <- 100 * sum(m1ab$sigma2) / (sum(m1ab$sigma2) + (m1ab$k-m1ab$p)/sum(diag(P)))
I2

##One-out analysis
#Create new data frame using with matrix for estimates when trial is removed
df10 <- data.frame(matrix(NA, ncol = 7, nrow = length(data10$Study)))
df10[, 1] <- data10$Study
names(df10) <- c("Study", "estimate", "se", "tval", "pval", "ci.lb", "ci.ub")
df10

#Create 'for loop' to run multilevel meta-analysis leaving one trial out and
#enter coefficients into the data-frame called "df"
for(i in 1:length(data10$Study)){
  df.loo10 <- data10[-i,]
  df.sens.loo10 <- rma.mv(yi, vi, random = list(~ 1 | effectID,
                                                 ~ 1 | modeID,
                                                 ~ 1 | studyID), tdist=TRUE,
                                                 data=df.loo10)
  re_table10 <- coef(summary(df.sens.loo10))
  df10[i,-1] <- re_table10
}

df10[with(df10, order(estimate)),]
## View results (effect estimates, se, pval etc. with each trial removed)
df10

####################
#Sensitivity analysis with R=0.3
m1ac <- rma.mv(yi1, vi1, random = list(~ 1 | effectID,
                                        ~ 1 | modeID,
                                        ~ 1 | studyID), tdist=TRUE, data=data10)
m1ac

#Sensitivity analysis with R=0.8
m1ad <- rma.mv(yi2, vi2, random = list(~ 1 | effectID,
                                        ~ 1 | modeID,
                                        ~ 1 | studyID), tdist=TRUE, data=data10)
m1ad

####################
#All task categories meta-analysis (Sleep Restriction and Exercise in PM)
data11 <- subset.data.frame(data3, TOTamvpm=="PM")

m1ae <- rma.mv(yi, vi, random = list(~ 1 | effectID,
                                      ~ 1 | modeID,
                                      ~ 1 | studyID), tdist=TRUE, data=data11)
m1ae

#Calculate I^2
W <- diag(1/data11$vi)
X <- model.matrix(m1ae)
P <- W - W %*% X %*% solve(t(X) %*% W %*% X) %*% t(X) %*% W
I2 <- 100 * sum(m1ae$sigma2) / (sum(m1ae$sigma2) + (m1ae$k-m1ae$p)/sum(diag(P)))
I2

##One-out analysis
#Create new data frame using with matrix for estimates when trial is removed
df11 <- data.frame(matrix(NA, ncol = 7, nrow = length(data11$Study)))
df11[, 1] <- data11$Study
names(df11) <- c("Study", "estimate", "se", "tval", "pval", "ci.lb", "ci.ub")
```

```
df11

#Create 'for loop' to run multilevel meta-analysis leaving one trial out and
#enter coefficients into the data-frame called "df"
for(i in 1:length(data11$Study)){
  df.loo11 <- data11[-i,]
  df.sens.loo11 <- rma.mv(yi, vi, random = list(~ 1 | effectID,
                                                ~ 1 | modeID,
                                                ~ 1 | studyID), tdist=TRUE,
                          control=list(optimizer="optim", optmethod="BFGS"),
                          data=df.loo11)
  re_table11 <- coef(summary(df.sens.loo11))
  df11[i,-1] <- re_table11
}

df11[with(df11, order(estimate)),]
## View results (effect estimates, se, pval etc. with each trial removed)
df11

####################
#Sensitivity analysis with R=0.3
m1af <- rma.mv(yi1, vi1, random = list(~ 1 | effectID,
                                       ~ 1 | modeID,
                                       ~ 1 | studyID), tdist=TRUE, data=data11)
m1af

#Sensitivity analysis with R=0.8
m1ag <- rma.mv(yi2, vi2, random = list(~ 1 | effectID,
                                       ~ 1 | modeID,
                                       ~ 1 | studyID), tdist=TRUE, data=data11)
m1ag

####################
#All task categories meta-analysis (Early Sleep Restriction and Exercise in AM)
data12 <- subset.data.frame(data4, TOTamvpm=="AM")

m1ah <- rma.mv(yi, vi, random = list(~ 1 | effectID,
                                     ~ 1 | modeID,
                                     ~ 1 | studyID), tdist=TRUE, data=data12)
m1ah

#Calculate I^2
W <- diag(1/data12$vi)
X <- model.matrix(m1ah)
P <- W - W %*% X %*% solve(t(X) %*% W %*% X) %*% t(X) %*% W
I2 <- 100 * sum(m1ah$sigma2) / (sum(m1ah$sigma2) + (m1ah$k-m1ah$p)/sum(diag(P)))
I2

##One-out analysis
#Create new data frame using with matrix for estimates when trial is removed
df12 <- data.frame(matrix(NA, ncol = 7, nrow = length(data12$Study)))
df12[, 1] <- data12$Study
names(df12) <- c("Study", "estimate", "se", "tval", "pval", "ci.lb", "ci.ub")
df12

#Create 'for loop' to run multilevel meta-analysis leaving one trial out and
#enter coefficients into the data-frame called "df"
for(i in 1:length(data12$Study)){
  df.loo12 <- data12[-i,]
  df.sens.loo12 <- rma.mv(yi, vi, random = list(~ 1 | effectID,
                                                ~ 1 | modeID,
                                                ~ 1 | studyID), tdist=TRUE,
                          control=list(optimizer="optim", optmethod="BFGS"),
                          data=df.loo12)
  re_table12 <- coef(summary(df.sens.loo12))
  df12[i,-1] <- re_table12
}

df12[with(df12, order(estimate)),]
## View results (effect estimates, se, pval etc. with each trial removed)
df12
```

```
####################
#Sensitivity analysis with R=0.3
m1ai <- rma.mv(yi1, vi1, random = list(~ 1 | effectID,
                                        ~ 1 | modeID,
                                        ~ 1 | studyID), tdist=TRUE,
               control=list(optimizer="optim", optmethod="BFGS"), data=data12)
m1ai

#Sensitivity analysis with R=0.8
m1aj <- rma.mv(yi2, vi2, random = list(~ 1 | effectID,
                                        ~ 1 | modeID,
                                        ~ 1 | studyID), tdist=TRUE, data=data12)
m1aj

####################
#All task categories meta-analysis (Early Sleep Restriction and Exercise in PM)
data13 <- subset.data.frame(data4, TOTamvpm=="PM")

m1ak <- rma.mv(yi, vi, random = list(~ 1 | effectID,
                                      ~ 1 | modeID,
                                      ~ 1 | studyID), tdist=TRUE, data=data13)
m1ak

#Calculate I^2
W <- diag(1/data13$vi)
X <- model.matrix(m1ak)
P <- W - W %*% X %*% solve(t(X) %*% W %*% X) %*% t(X) %*% W
I2 <- 100 * sum(m1ak$sigma2) / (sum(m1ak$sigma2) + (m1ak$k-m1ak$p)/sum(diag(P)))
I2

##One-out analysis
#Create new data frame using with matrix for estimates when trial is removed
df13 <- data.frame(matrix(NA, ncol = 7, nrow = length(data13$Study)))
df13[, 1] <- data13$Study
names(df13) <- c("Study", "estimate", "se", "tval", "pval", "ci.lb", "ci.ub")
df13

#Create 'for loop' to run multilevel meta-analysis leaving one trial out and
#enter coefficients into the data-frame called "df"
for(i in 1:length(data13$Study)){
  df.loo13 <- data13[-i,]
  df.sens.loo13 <- rma.mv(yi, vi, random = list(~ 1 | effectID,
                                                 ~ 1 | modeID,
                                                 ~ 1 | studyID), tdist=TRUE,
                                                 data=df.loo13)
  re_table13 <- coef(summary(df.sens.loo13))
  df13[i,-1] <- re_table13
}

df13[with(df13, order(estimate)),]
## View results (effect estimates, se, pval etc. with each trial removed)
df13

####################
#Sensitivity analysis with R=0.3
m1al <- rma.mv(yi1, vi1, random = list(~ 1 | effectID,
                                        ~ 1 | modeID,
                                        ~ 1 | studyID), tdist=TRUE,  data=data13)
m1al

#Sensitivity analysis with R=0.8
m1am <- rma.mv(yi2, vi2, random = list(~ 1 | effectID,
                                        ~ 1 | modeID,
                                        ~ 1 | studyID), tdist=TRUE, data=data13)
m1am

####################
#All task categories meta-analysis (Late Sleep Restriction and Exercise in AM)
data14 <- subset.data.frame(data5, TOTamvpm=="AM")
```

```
m1an <- rma.mv(yi, vi, random = list(~ 1 | effectID,
                                     ~ 1 | modeID,
                                     ~ 1 | studyID), tdist=TRUE, data=data14)
m1an

#Calculate I^2
W <- diag(1/data14$vi)
X <- model.matrix(m1an)
P <- W - W %*% X %*% solve(t(X) %*% W %*% X) %*% t(X) %*% W
I2 <- 100 * sum(m1an$sigma2) / (sum(m1an$sigma2) + (m1an$k-m1an$p)/sum(diag(P)))
I2

##One-out analysis
#Create new data frame using with matrix for estimates when trial is removed
df14 <- data.frame(matrix(NA, ncol = 7, nrow = length(data14$Study)))
df14[, 1] <- data14$Study
names(df14) <- c("Study", "estimate", "se", "tval", "pval", "ci.lb", "ci.ub")
df14

#Create 'for loop' to run multilevel meta-analysis leaving one trial out and
#enter coefficients into the data-frame called "df"
for(i in 1:length(data14$Study)){
  df.loo14 <- data14[-i,]
  df.sens.loo14 <- rma.mv(yi, vi, random = list(~ 1 | effectID,
                                                ~ 1 | modeID,
                                                ~ 1 | studyID), tdist=TRUE,
                          control=list(optimizer="optim", optmethod="BFGS"),
                          data=df.loo14)
  re_table14 <- coef(summary(df.sens.loo14))
  df14[i,-1] <- re_table14
}

df14[with(df14, order(estimate)),]
## View results (effect estimates, se, pval etc. with each trial removed)
df14

####################
#Sensitivity analysis with R=0.3
m1ao <- rma.mv(yi1, vi1, random = list(~ 1 | effectID,
                                       ~ 1 | modeID,
                                       ~ 1 | studyID), tdist=TRUE,  data=data14)
m1ao

#Sensitivity analysis with R=0.8
m1ap <- rma.mv(yi2, vi2, random = list(~ 1 | effectID,
                                       ~ 1 | modeID,
                                       ~ 1 | studyID), tdist=TRUE, data=data14)
m1ap

####################
#All task categories meta-analysis (Late Sleep Restriction and Exercise in PM)
data15 <- subset.data.frame(data5, TOTamvpm=="PM")

View(data15)

m1aq <- rma.mv(yi, vi, random = list(~ 1 | effectID,
                                     ~ 1 | modeID,
                                     ~ 1 | studyID), tdist=TRUE, data=data15)
m1aq

#Calculate I^2
W <- diag(1/data15$vi)
X <- model.matrix(m1aq)
P <- W - W %*% X %*% solve(t(X) %*% W %*% X) %*% t(X) %*% W
I2 <- 100 * sum(m1aq$sigma2) / (sum(m1aq$sigma2) + (m1aq$k-m1aq$p)/sum(diag(P)))
I2

##One-out analysis
#Create new data frame using with matrix for estimates when trial is removed
df15 <- data.frame(matrix(NA, ncol = 7, nrow = length(data15$Study)))
df15[, 1] <- data15$Study
```

```
names(df15) <- c("Study", "estimate", "se", "tval", "pval", "ci.lb", "ci.ub")
df15

#Create 'for loop' to run multilevel meta-analysis leaving one trial out and
#enter coefficients into the data-frame called "df"
for(i in 1:length(data15$Study)){
  df.loo15 <- data15[-i,]
  df.sens.loo15 <- rma.mv(yi, vi, random = list(~ 1 | effectID,
                                                ~ 1 | modeID,
                                                ~ 1 | studyID), tdist=TRUE,
                                                data=df.loo15)
  re_table15 <- coef(summary(df.sens.loo15))
  df15[i,-1] <- re_table15
}

df15[with(df15, order(estimate)),]
## View results (effect estimates, se, pval etc. with each trial removed)
df15

####################
#Sensitivity analysis with R=0.3
m1ar <- rma.mv(yi1, vi1, random = list(~ 1 | effectID,
                                       ~ 1 | modeID,
                                       ~ 1 | studyID), tdist=TRUE,  data=data15)
m1ar

#Sensitivity analysis with R=0.8
m1as <- rma.mv(yi2, vi2, random = list(~ 1 | effectID,
                                       ~ 1 | modeID,
                                       ~ 1 | studyID), tdist=TRUE, data=data15)
m1as

#############################################################
#All task categories meta-regression analysis

#Overall meta-regression (multi-level) for sleep loss
sleep_loss_mr<- rma.mv(yi, vi, random = list(~ 1 | effectID,
                                             ~ 1 | modeID,
                                             ~ 1 | studyID), tdist=TRUE, mods =  ~
                                             Timeawake, data=data)
sleep_loss_mr

#Meta-regression (multi-level) for sleep deprivation
sleep_dep_mr<- rma.mv(yi, vi, random = list(~ 1 | effectID,
                                            ~ 1 | modeID,
                                            ~ 1 | studyID), tdist=TRUE, mods =  ~
                                            Timeawake, data=data2)
sleep_dep_mr

#Remove "NA" rows for Sleep Restriction meta-regression
data3a <- data3[ which(data3$Timeawake != "NA"), ]

#Meta-regression (multi-level) for sleep restriction
sleep_res_mr<- rma.mv(yi, vi, random = list(~ 1 | effectID,
                                            ~ 1 | modeID,
                                            ~ 1 | studyID), tdist=TRUE, mods =  ~
                                            Timeawake, data=data3a)
sleep_res_mr

#Remove "NA" rows for Early Restriction meta-regression
data4a <- data4[ which(data4$Timeawake != "NA"), ]

#Meta-regression (multi-level) for sleep restriction
sleep_earlyres_mr<- rma.mv(yi, vi, random = list(~ 1 | effectID,
                                                 ~ 1 | modeID,
                                                 ~ 1 | studyID), tdist=TRUE, mods =
                                                 ~ Timeawake, data=data4a)
sleep_earlyres_mr

#Remove "NA" rows for Late Restriction meta-regression
data5a <- data5[ which(data5$Timeawake != "NA"), ]
```

```
#Meta-regression (multi-level) for sleep restriction
sleep_lateres_mr<- rma.mv(yi, vi, random = list(~ 1 | effectID,
                                                ~ 1 | modeID,
                                                ~ 1 | studyID), tdist=TRUE, mods =
                                                ~ Timeawake, data=data5a)
sleep_lateres_mr

####Meta-regression Plots
##create a new empty data to fill with predictions for Sleep Deprivation (SD) data
SDdata<-c()

pred_scores<-predict(sleep_dep_mr, newmods=cbind(seq(23.5,40,1)),addx=T)
SDdata<-rbind(SDdata,data.frame(pred_scores))

SDdata

###################Allocate size of data points based on study weighting (from
https://www.metafor-project.org/doku.php/tips:weights_in_rma.mv_models)
W_v1 <- weights(sleep_dep_mr, type = "matrix")
sleep_dep_mr$w_ij_metafor <- colSums(W_v1)/sum(W_v1)
size <- sleep_dep_mr$w_ij_metafor

##Average the predicted data from each outcome to get aggregate SD plot data
plotData <- data.frame("pred" = aggregate(x = SDdata$pred, by =
list(SDdata$X.Timeawake), FUN = mean),
                       "ci.lb" = aggregate(x = SDdata$ci.lb, by =
                       list(SDdata$X.Timeawake), FUN = mean),
                       "ci.ub" = aggregate(x = SDdata$ci.ub, by =
                       list(SDdata$X.Timeawake), FUN = mean),
                       "time" = aggregate(x = SDdata$X.Timeawake, by =
                       list(SDdata$X.Timeawake), FUN = mean))

##Develop Sleep deprivation plot (averaged across all exercise modes)
myplotSD <- ggplot(plotData, aes(x=time.x, y=pred.x))+
  scale_x_continuous(expand = c(0, 0), limits = c(23.5,38.5), breaks=seq(24,39,1)) +
  scale_y_continuous(expand = c(0, 0), limits = c(-50, 25), breaks=seq(-50,25,10))+
  geom_ribbon(aes(x=time.x, ymin=ci.lb.x, ymax=ci.ub.x), fill = "grey90")+
  geom_path(aes(x=time.x,y=pred.x),lty=1, lwd=1, colour="firebrick")+
  geom_point(data=data2, aes(x=Timeawake,y=yi, size=size), fill=NA, shape=21, stroke
  = 0.7, alpha=1)+
  theme_linedraw(12)+
  xlab("Time Awake (hrs)")+
  ylab("% Difference")+
  theme_classic()+
  theme(axis.title.x = element_text(face="bold", size=16, vjust=-1),
        axis.title.y = element_text(face="bold", size=16, vjust=2.5))+
  theme(legend.position = "none")+
  theme(axis.text = element_text(size=14, colour = "black"))

###########create a new empty data to fill with predictions for Sleep Restriction
(SR) data
SRdata<-c()

pred_scores2<-predict(sleep_res_mr, newmods=cbind(seq(0,18,0.5)),addx=T)
SRdata<-rbind(SRdata,data.frame(pred_scores2))

SRdata

##################Allocate size of data points based on study weighting
W_v1a <- weights(sleep_res_mr, type = "matrix")
sleep_res_mr$w_ij_metafor <- colSums(W_v1a)/sum(W_v1a)
size2 <- sleep_res_mr$w_ij_metafor

##Add weights to SR dataframe
data3a$wt <- size2

##Average the predicted data from each outcome to get aggregate SR plot data
plotData2 <- data.frame("pred" = aggregate(x = SRdata$pred, by =
list(SRdata$X.Timeawake), FUN = mean),
                        "ci.lb" = aggregate(x = SRdata$ci.lb, by =
```

```
                                   list(SRdata$X.Timeawake), FUN = mean),
                                   "ci.ub" = aggregate(x = SRdata$ci.ub, by =
                                   list(SRdata$X.Timeawake), FUN = mean),
                                   "time" = aggregate(x = SRdata$X.Timeawake, by =
                                   list(SRdata$X.Timeawake), FUN = mean))

##Develop Sleep restriction plot (averaged across all exercise modes)
myplotSR <- ggplot(plotData2, aes(x=time.x, y=pred.x))+
  scale_x_continuous(expand = c(0, 0), limits = c(0,16.5), breaks=seq(0,17,1)) +
  scale_y_continuous(expand = c(0, 0), limits = c(-40, 10), breaks=seq(-40,10,5))+
  geom_ribbon(aes(x=time.x, ymin=ci.lb.x, ymax=ci.ub.x), fill = "grey90")+
  geom_path(aes(x=time.x,y=pred.x),lty=1, lwd=1, colour="firebrick")+
  geom_point(data=data3a[ which(data3a$Sleepprotocol=="Sleep restriction"), ],
             aes(x=Timeawake,y=yi,size=wt), fill=NA, shape=21, stroke = 0.7,
             alpha=1, color="chartreuse4")+
  geom_point(data=data3a[ which(data3a$Sleepprotocol=="Early restriction"), ],
             aes(x=Timeawake,y=yi,size=wt), fill=NA, shape=21, stroke = 0.7,
             alpha=1, color="deepskyblue4")+
  geom_point(data=data3a[ which(data3a$Sleepprotocol=="Late restriction"), ],
             aes(x=Timeawake,y=yi,size=wt), fill=NA, shape=21, stroke = 0.7,
             alpha=1, color="darkorange2")+
  theme_linedraw(12)+
  xlab("Time Awake (hrs)")+
  ylab("% Difference")+
  theme_classic()+
  theme(axis.title.x = element_text(face="bold", size=16, vjust=-1),
        axis.title.y = element_text(face="bold", size=16, vjust=2.5))+
  theme(legend.position = "none")+
  theme(axis.text = element_text(size=14, colour = "black"))

########Facet Plot for SD & SR
png("MR_Plot.png", units='in', width=10,height=10,res=1200)
ggarrange(myplotSR, myplotSD, labels = c("a", "b"), nrow = 2)
dev.off()
```
