# Supplementary material for: Effects of Acute Sleep Loss on Physical Performance: A Systematic and Meta-Analytical Review
Source: Sports Med. 2022 Jun 16;52(11):2669–90. doi: 10.1007/s40279-022-01706-y (PMC9584849; doi:10.1007/s40279-022-01706-y)
Supplement: Supplementary file 3 — Supplementary file3 (DOCX 3708 KB) [file 40279_2022_1706_MOESM3_ESM.docx]

**Supplementary material**

**Supplementary Table S1:** Original search breakdown

| **Database search** | **Date** | **Search terms** | **Restriction** | **Records Identified** |
| --- | --- | --- | --- | --- |
| Web of Science | 25.08.2020 | TS=((sleep restriction or sleep deprivation or sleep loss or wakefulness) and (exercis* or performance) not (animal* or rat* or mice)) | Restricted to LANGUAGES: (ENGLISH) AND DOCUMENT TYPES: (ARTICLE) | 3787 |
| SCOPUS | 25.08.2020 | TITLE-ABS-KEY ("sleep restriction"  OR  "sleep deprivation"  OR  "sleep loss"  OR  wakefulness  AND  exercis*  OR  performance  AND NOT  animal*  OR  rat*  OR  mice) | (LIMIT-TO (DOCTYPE, "ar")) AND (LIMIT-TO (LANGUAGE, "English")) | 3457 |
| PsycInfo | 25.08.2020 | (((Sleep restriction or sleep deprivation or wakefulness or Sleep loss) and (exercis* or performance)) not (animal* or rat* or mice)) | mp. [mp=title, abstract, heading word, table of contents, key concepts, original title, tests & measures, mesh] | 1695 |
|  |  |  | **Total** | **8939** |

**Supplementary Table** **S2**: Origin of included publications

| **Lead author** | **Year** | **Title** | **Source** |
| --- | --- | --- | --- |
| Abedelmalek | 2014 | Effect of partial sleep deprivation and racial variation on short-term maximal performance | Original search |
| Abedelmalek | 2013 | Effect of time of day and partial sleep deprivation on plasma concentrations of IL-6 during a short-term maximal performance | Original search |
| Bambaeichi | 2005 | The influence of time of day and partial sleep loss on muscle strength in eumenorrheic females | Original search |
| Brodan and Kuhn | 1967 | Physical performance in man during sleep deprivation | Original search |
| Bulbulian | 1996 | The effect of sleep deprivation and exercise load on isokinetic leg strength and endurance | Original search |
| Chase | 2017 | One night of sleep restriction following heavy exercise impairs 3-km cycling time-trial performance in the morning | Original search |
| Cook | 2012 | Acute Caffeine Ingestion’s Increase of Voluntarily Chosen Resistance-Training Load After Limited Sleep | Original search |
| Cook | 2011 | Skill execution and sleep deprivation: effects of acute caffeine or creatine supplementation - a randomized placebo-controlled trial | Original search |
| Cullen | 2019 | The effects of a single night of complete and partial sleep deprivation on physical and cognitive performance: A Bayesian analysis | Original search |
| Daaloul | 2019 | Effects of Napping on Alertness, Cognitive, and Physical Outcomes of Karate Athletes | Original search |
| Daviaux | 2014 | Effects of total sleep deprivation on the perception of action capabilities | Original search |
| Edwards | 2009 | Effects of One Night of Partial Sleep Deprivation upon Diurnal Rhythms of Accuracy and Consistency in Throwing Darts | Original search |
| Goh | 2001 | Effects of one night of sleep deprivation on hormone profiles and performance efficiency | Original search |
| Gonçalves | 2020 | Effects of acute sleep deprivation on H reflex and V wave | Original search |
| Goto | 2020 | Partial sleep deprivation after an acute exercise session does not augment hepcidin levels the following day | Original search |
| Khcharem | 2020 | Effects of caffeine ingestion on 8-km run performance and cognitive function after 26 hours of sleep deprivation | Original search |
| Kujawa | 2020 | The Influence of 24-Hour Sleep Deprivation on the Strength of Lower Limb Muscles in Young and Physically Fit Women and Men | Original search |
| Martin | 1981 | Effect of sleep-deprivation on tolerance of prolonged exercise | Original search |
| Moore | 2018 | Effects of acute sleep deprivation and caffeine supplementation on anaerobic performance | Original search |

**Supplementary Table S2**: continued

| **Lead author** | **Year** | **Title** | **Source** |
| --- | --- | --- | --- |
| Mougin | 2001 | Hormonal responses to exercise after partial sleep deprivation and after a hypnotic drug-induced sleep deprivation and after a hypnotic drug-induced sleep | Original search |
| Mougin | 1996 | Effects of a selective sleep deprivation on subsequent anaerobic performance | Original search |
| Ozturk | 2007 | Effects of sleep deprivation on anaerobic exercise-induced changes in auditory brainstem evoked potentials | Original search |
| Racinais | 2004 | Effect of sleep deprivation on shuttle run score in middle-aged amateur athletes: Influence of initial score | Original search |
| Rae | 2017 | One night of partial sleep deprivation impairs recovery from a single exercise training session | Original search |
| Roberts | 2019 | Extended Sleep Maintains Endurance Performance Better than Normal or Restricted Sleep | Original search |
| Romdhani | 2020 | Improved Physical Performance and Decreased Muscular and Oxidative Damage With Post-lunch Napping After Partial Sleep Deprivation in Athletes | Original search |
| Romdhani | 2019 | Sleep deprivation affects post-lunch dip performances, biomarkers of muscle damage and antioxidant status | Original search |
| Skein | 2013 | The effect of overnight sleep deprivation following competitive rugby league matches 2 on post-match physiological and perceptual recovery | Original search |
| Skurvydas | 2020 | One night of sleep deprivation impairs executive function but does not affect psychomotor or motor performance | Original search |
| Souissi | 2014 | The effects of caffeine ingestion on the reaction time and short-term maximal performance after 36 h of sleep deprivation | Original search |
| Souissi | 2019 | The effects of caffeine on the diurnal variation of the reaction time and short-term maximal performance after one night of sleep deprivation | Original search |
| Souissi | 2003 | Effects of one night’s sleep deprivation on anaerobic performance the following day | Original search |
| Souissi | 2008 | Effect of Time of Day and Partial Sleep Deprivation on Short-Term, High-Power Output | Original search |
| Souissi | 2015 | Morning caffeine ingestion increases cognitive function and short-term maximal performance in footballer players after partial sleep deprivation | Original search |
| Souissi | 2018 | Caffeine reversal of sleep deprivation effects on alertness, mood and repeated sprint performances in physical education students | Original search |

**Supplementary Table** **S2**: continued

| **Lead author** | **Year** | **Title** | **Source** |
| --- | --- | --- | --- |
| Souissi | 2020 | Effects of a 30-min nap opportunity on cognitive and short-duration high-intensity performances and mood states after a partial sleep deprivation night | Original search |
| Souissi | 2013 | Effects of time-of-day and partial sleep deprivation on short-term maximal performances of judo competitors | Original search |
| Sweeney | 2020 | Impaired Insulin ProFiles Following a Single Night of Sleep Restriction: The Impact of Acute Sprint Interval Exercise | Original search |
| Taheri | 2012 | The effect of sleep deprivation on choice Reaction time and anaerobic power of college student athletes | Original search |
| Vardar | 2007 | Sleep deprivation induced anxiety and anaerobic performance | Original search |
| Azboy | 2009 | Effects of sleep deprivation on cardiorespiratory functions of the runners and volleyball players during rest and exercise | Fullagar back citation |
| Froberg | 1974 | Circadian rhythms of catecholamine excretion, shooting range performance and self-ratings of fatigue during sleep deprivation | Fullagar back citation |
| HajSalem | 2013 | Effects of partial sleep deprivation at the end of the night on anaerobic performances in judokas | Fullagar back citation |
| Hill | 1994 | Aerobic and anaerobic contributions to exhaustive high-intensity exercise after sleep deprivation | Fullagar back citation |
| Mejri | 2013 | Effect of two types of partial sleep deprivation on Taekwondo players’ performance during intermittent exercise | Fullagar back citation |
| Oliver | 2009 | One night of sleep deprivation decreases treadmill endurance performance | Fullagar back citation |
| Reilly | 1994 | The effect of partial sleep deprivation on weight-lifting performance | Fullagar back citation |
| Reyner | 2013 | Sleep restriction and serving accuracy in performance tennis players, and effects of caffeine | Fullagar back citation |
| Skein | 2011 | Intermittent-sprint performance and muscle glycogen after 30 h of sleep deprivation | Fullagar back citation |
| Temesi | 2012 | Does central fatigue explain reduced cycling after complete sleep deprivation? | Fullagar back citation |
| Ajjimaporn | 2020 | Effects of a 20-min Nap after Sleep Deprivation on Brain Activity and Soccer Performance | Forward citation Fullagar - Google Scholar |
| Arazi | 2019 | Effects of Overnight Sleep Deprivation on Appetite and Physical Performance in Elite Female Soccer Players | Forward citation Fullagar - Google Scholar |

**Supplementary Table** **S2**: continued

| **Lead author** | **Year** | **Title** | **Source** |
| --- | --- | --- | --- |
| Dáttilo | 2020 | Effects of Sleep Deprivation on Acute Skeletal Muscle Recovery after Exercise | Forward citation Fullagar - Google Scholar |
| Blumert | 2007 | The acute effects of twenty-four hours of sleep loss on the performance of national-caliber male collegiate weightlifters | Knowles back citation |
| Chen | 1991 | Effects of 30-h sleep loss on cardiorespiratory functions at rest and in exercise. | Azboy 2009 |
| Omiya | 2009 | Heart-Rate Response to Sympathetic Nervous Stimulation, Exercise, and Magnesium Concentration in Various Sleep Conditions | Chase et al |
| Mejri | 2016 | Does one night of partial sleep deprivation affect the evening performance during intermittent exercise in Taekwondo players? | Chase et al |
| Mougin | 1991 | Effects of sleep disturbances on subsequent physical performance | Chase et al |
| Roberts | 2019 | Effects of total sleep deprivation on endurance cycling performance and heart rate indices used for monitoring athlete readiness | Goto et al. 2020 |
| Holland | 1967 | Effects of Limited Sleep Deprivation on Performance of Selected Motor Tasks | Mougin et al. 1996 |
| Romdhani | 2021 | Total sleep deprivation and recovery sleep affect the diurnal variation of agility performance: the gender differences | Romdhani et al. 2020 |
| Baati | 2020 | Effect of Angle of View and Partial Sleep Deprivation on Distance Perception | Souissi et al. 2020 |
| Baati | 2015 | Effect of total sleep deprivation on egocentric distance estimation following a fatiguing task | Souissi et al. 2020 |
| Arnal | 2016 | Sleep Extension before Sleep Loss: Effects on Performance and Neuromuscular Function | Skurvydas et al. 2020 |
| Khemila | 2021 | Listening to motivational music during warming- up attenuates the negative effects of partial sleep deprivation on cognitive and short-term maximal performance: Effect of time of day | Updated search |
| Filipas | 2021 | Single and Combined Effect of Acute Sleep Restriction and Mental Fatigue on Basketball Free-Throw Performance | Updated search |
| Romdhani | 2021 | Caffeine Use or Napping to Enhance Repeated Sprint Performance After Partial Sleep Deprivation: Why Not Both? | Updated search |
| Rodrigues | 2021 | Combined and isolated effects of alcohol consumption and sleep deprivation on maximal strength, muscle endurance and aerobic exercise performance in healthy men: a cross‐over randomized controlled trial | Updated search |
| Paryab | 2021 | Melatonin supplementation improves psychomotor and physical performance in collegiate student-athletes following a sleep deprivation night | Updated search |

**Supplementary Table S3:** Reason for full-text publication exclusion

| **Lead author** | **Year** | **Title** | **Reason for exclusion** |
| --- | --- | --- | --- |
| Abedelmalek | 2013 | Effects of Partial Sleep Deprivation on Proinflammatory Cytokines, Growth Hormone, and Steroid Hormone Concentrations During Repeated Brief Sprint Interval Exercise | No exercise performance measured |
| Ablin | 2013 | Effects of sleep restriction and exercise deprivation on somatic symptoms and mood in healthy adults | No exercise performance measured |
| Bunnell | 1984 | Sleep interruption and exercise | No exercise performance measured |
| Goodman | 1990 | Moderate exercise and hemodilution during sleep-deprivation | No exercise performance measured |
| Kojima | 2020 | Acute moderate-intensity exercise improves 24-h sleep deprivation-induced cognitive decline and cerebral oxygenation: A near-infrared spectroscopy study | No exercise performance measured |
| Mejri | 2015 | One night of partial sleep deprivation affects biomarkers of cardiac damage, but not cardiovascular and lipid profiles, in young athletes | No exercise performance measured |
| Strausba | 1970 | Ego strength, skin conductance, sleep deprivation, and performance | No exercise performance measured |
| Vgontzas | 2007 | Daytime napping after a night of sleep loss decreases sleepiness, improves performance, and causes beneficial changes in cortisol and interleukin-6 secretion | No exercise performance measured |
| Webb | 1973 | Effects of performance of high and low energy expenditure during sleep deprivation | No exercise performance measured |
| Buck | 1975 | Sleep loss effects on movement time | No exercise performance measured |
| Daftuar | 1972 | Sleep deprivation and human performance | No exercise performance measured |
| Kondo | 1990 | Effects of exercise and sleep deprivation on serum zinc | No exercise performance measured |
| LeDuc | 2000 | The effects of exercise as a countermeasure for fatigue in sleep-deprived aviators | No exercise performance measured |
| Martin | 1982 | Self-selected exercise intensity is unchanged by sleep loss | No exercise performance measured |
| Martin | 1986 | Stress hormonal response to exercise after sleep loss | No exercise performance measured |
| McMurray | 1984 | The effect of sleep loss on high-intensity exercise and recovery | No exercise performance measured |
| McMurray | 1988 | The endorphin response of women to sleep loss and exercise | No exercise performance measured |

**Supplementary Table** **S3**: continued

| **Lead author** | **Year** | **Title** | **Reason for exclusion** |
| --- | --- | --- | --- |
| Mougin | 1992 | Met-enkephalin, β-endorphin and cortisol responses to sub-maximal exercise after sleep disturbances | No exercise performance measured |
| Plyley | 1987 | Sleep-deprivation and cardiorespiratory function - influence of intermittent submaximal exercise | No exercise performance measured |
| Sawka | 1984 | Effects of sleep-deprivation on thermoregulation during exercise | No exercise performance measured |
| McNeil | 2016 | The effects of sleep restriction and altered sleep timing on energy intake and energy expenditure | No exercise performance measured |
| Tajiri | 2018 | Effect of sleep curtailment on dietary behavior and physical activity: A randomized crossover trial | No exercise performance measured |
| Jarraya | 2013 | The effect of partial sleep deprivation on the reaction time and the attentional capacities of the handball goalkeeper | No exercise performance measured |
| Jarraya | 2014 | Effect of time of day and partial sleep deprivation on the reaction time and the attentional capacities of the handball goalkeeper | No exercise performance measured |
| Ingram | 2015 | Sleep disruption and its effect on lymphocyte redeployment following an acute bout of exercise | No exercise performance measured |
| Martin | 1981 | Exercise after sleep-deprivation | No exercise performance measured |
| Kolka | 1988 | Exercise thermoregulation after prolonged wakefulness | No exercise performance measured |
| Chen | 1989 | Sleep loss impairs inspiratory muscle endurance | No exercise performance measured |
| Wilkinson | 1961 | Performance under sleep-deprivation | No exercise performance measured |
| Akazawa | 2019 | Effect of sleep efficiency on salivary metabolite profile and cognitive function during exercise in volleyball athletes | No sleep intervention |
| Antunes | 2017 | Sleep quality and duration are associated with performance in maximal incremental test | No sleep intervention |
| Auyeung | 2015 | Sleep Duration and Disturbances Were Associated With Testosterone Level, Muscle Mass, and Muscle Strength-A Cross-Sectional Study in 1274 Older Men | No sleep intervention |
| Bonnet | 1995 | The use of caffeine versus prophylactic naps in sustained performance | No sleep intervention |
| Bougard | 2009 | Effects of Waking Time and Breakfast Intake Prior to Evaluation of Physical Performance in the Early Morning | No sleep intervention |
| de Mello | 2020 | Duration and quality of sleep in sprint and recovery performances among elite swimmers | No sleep intervention |

**Supplementary Table** **S3**: continued

| **Lead author** | **Year** | **Title** | **Reason for exclusion** |
| --- | --- | --- | --- |
| Driller | 2019 | The influence of night-time electronic device use on subsequent sleep and propensity to be physically active the following day | No sleep intervention |
| Ito | 2007 | Acute effects of zolpidem on daytime alertness, psychomotor and physical performance | No sleep intervention |
| Jarraya | 2014 | Diurnal variations on cognitive performances in handball goalkeepers | No sleep intervention |
| Kline | 2007 | Circadian variation in swim performance | No sleep intervention |
| Matsumoto | 2002 | Physical activity increases the dissociation between subjective sleepiness and objective performance levels during extended wakefulness in human | No sleep intervention |
| Souissi | 2013 | Effects of three types of chronobiotics on anaerobic performances and their diurnal variations | No sleep intervention |
| Taheri | 2020 | Morning exercise improves cognitive performance decrements induced by partial sleep deprivation in elite athletes | No sleep intervention |
| Hammouda | 2018 | Diurnal napping after partial sleep deprivation affected hematological and biochemical responses during repeated sprint | No sleep intervention |
| Pierce | 1993 | The effects of an acute bout of sleep on running economy and VO2 max | No sleep intervention |
| Petit | 2014 | A 20-min nap in athletes changes subsequent sleep architecture but does not alter physical performances after normal sleep or 5-h phase-advance conditions | No sleep intervention |
| Angus | 1985 | Effects of prolonged sleep deprivation, with and without chronic physical exercise, on mood and performance | >1 day sleep loss |
| Tharion | 2003 | Caffeine effects on marksmanship during high-stress military training with 72 hour sleep deprivation | >1 day sleep loss |
| Bond | 1986 | Effects of sleep-deprivation on performance during submaximal and maximal exercise | >1 day sleep loss |
| Keramidas | 2018 | A brief pre-exercise nap may alleviate physical performance impairments induced by short-term sustained operations with partial sleep deprivation - A field-based study | >1 day sleep loss |
| Myles | 1985 | Sleep-deprivation, physical fatigue, and the perception of exercise intensity | >1 day sleep loss |
| Kolka | 1984 | Exercise in a cold environment after sleep-deprivation | >1 day sleep loss |
| Goodman | 1989 | Maximal aerobic exercise following prolonged sleep-deprivation | >1 day sleep loss |
| Martin | 1984 | Sleep loss and the sympathoadrenal response to exercise | >1 day sleep loss |

**Supplementary Table** **S3**: continued

| **Lead author** | **Year** | **Title** | **Reason for exclusion** |
| --- | --- | --- | --- |
| Symons | 1988 | Physical performance and physiological responses following 60 hours of sleep deprivation | >1 day sleep loss |
| Takeuchi | 1985 | Sleep deprivation, chronic exercise and muscular performance | >1 day sleep loss |
| Chen | 2017 | Relationship between sleep and muscle strength among Chinese university students: a cross-sectional study | Observational |
| Facer-Childs | 2015 | Circadian phenotype composition is a major predictor of diurnal physical performance in teams | Observational |
| Facer-Child | 2018 | The effects of time of day and chronotype on cognitive and physical performance in healthy volunteers | Observational |
| Daniel | 2019 | Effect of the intake of high or low glycemic index high carbohydrate-meals on athletes' sleep quality in pre-game nights | Observational |
| Buman | 2011 | Moderators and mediators of exercise-induced objective sleep improvements in midlife and older adults with sleep complaints | Observational |
| Carazo-Vargas | 2020 | The association between sleep efficiency and physical performance in taekwondo athletes | Observational |
| Brandt | 2017 | Perceived Sleep Quality, Mood States, and Their Relationship With Performance Among Brazilian Elite Athletes During a Competitive Period | Observational |
| Hong | 2018 | Sleep and Performance | Review |
| Martin | 1988 | Sleep loss and subsequent exercise performance | Review |
| Erich | 2011 | Dead tired. There's no question sleep deprivation and fatigue can harm performance. How bad is it in EMS, and what can we do about it? | Review |
| Brodan | 1969 | Changes of mental and physical performance in sleep deprivated healthy volunteers | Review |
| Cheikh | 2017 | Effects of one-night sleep deprivation on selective attention and isometric force in adolescent karate athletes | Children |
| Cheikh | 2020 | Melatonin ingestion after exhaustive late-evening exercise attenuate muscle damage, oxidative stress, and inflammation during intense short term effort in the following day in teenage athletes | Children |
| Suppiah | 2016 | Restricted and unrestricted sleep schedules of Asian adolescent, high-level student athletes: effects on sleep durations, marksmanship and cognitive performance | Children |
| Pallesen | 2017 | The Effects of Sleep Deprivation on Soccer Skills | Children |
| Mah | 2019 | Sleep restriction impairs maximal jump performance and joint coordination in elite athletes | Consecutive nights of sleep restriction |

**Supplementary Table S4:** Location of sleep protocol and method used to monitor sleep duration/quality

| **Exercise category** | **Location of sleep trials** | | | | |
| --- | --- | --- | --- | --- | --- |
|  | **Lab for both Int and Con** | **Lab, only Int, Con - home** | **Lab, only Int, Con not described** | **Home unsupervised** | **Not described** |
| All categories | 113 | 53 | 19 | 28 | 14 |
| Anaerobic power | 41 | 12 | 1 | 1 | 3 |
| Speed/power endurance | 23 | 8 | 0 | 1 | 0 |
| HIIE | 17 | 2 | 0 | 5 | 3 |
| Strength | 22 | 21 | 13 | 4 | 6 |
| Endurance | 7 | 7 | 3 | 3 | 2 |
| Strength-endurance | 3 | 3 | 0 | 3 | 0 |
| Skill | 0 | 0 | 2 | 11 | 0 |
| **Exercise category** | **Method of sleep monitoring** | | | | |
|  | **Polysomnography** | **Actigraphy** | **Diary/questionnaire** | **Not reported** | |
| All tasks | 5 | 72 | 11 | 139 (54% sleep restriction) | |
| Anaerobic power | 0 | 22 | 0 | 36 (58% sleep restriction) | |
| Speed/power endurance | 0 | 11 | 0 | 21 (62% sleep restriction) | |
| HIIE | 0 | 11 | 2 | 14 (57% sleep restriction) | |
| Strength | 1 | 15 | 1 | 49 (59% sleep restriction) | |
| Endurance | 3 | 8 | 2 | 9 (0% sleep restriction) | |
| Strength-endurance | 1 | 0 | 4 | 4 (0% sleep restriction) | |
| Skill | 0 | 5 | 0 | 6 (67% sleep restriction) | |

**Abbreviations**: HIIE: high-intensity interval exercise; Int: Intervention (sleep loss protocol – either deprivation or restriction); Con: control (normal sleep). Lab for both Int and Con: sleep was monitored in the lab for both conditions; Lab, only Int, Con - home: only the intervention (sleep loss) condition was monitored in the lab and control (normal sleep) were permitted to sleep at home; Lab, only Int, Con not described: intervention condition were monitored in the lab and control was not described; Home unsupervised: both conditions slept at home unsupervised; Not described: no description provided. Sleep restriction: total sleep time ≤6 h in any 24 h period – this category is a combination of early-restriction, late-restriction, fragmented sleep and sleep restriction protocols not specified.

**Supplementary Table S5:** Mode of exercise and time of day tasks were undertaken

| **Exercise category** | **Mode of exercise task performed** | | | | | | | |
| --- | --- | --- | --- | --- | --- | --- | --- | --- |
|  | **Cycling (n)** | **Running (n)** | **Resistance (n)** | **Body-weight (n)** | **Walking (n)** | **Step (n)** | **Karate (n)** | **Skill (n)** |
| All categories | 92 | 37 | 69 | 13 | 1 | 1 | 1 | 13 |
| Anaerobic power | 35 | 11 | 0 | 12 | 0 | 0 | 0 | 0 |
| Speed/power endurance | 28 | 3 | 0 | 1 | 0 | 0 | 0 | 0 |
| HIIE | 8 | 18 | 0 | 0 | 0 | 0 | 1 | 0 |
| Strength | 6 | 0 | 60 | 0 | 0 | 0 | 0 | 0 |
| Endurance | 15 | 5 | 0 | 0 | 1 | 1 | 0 | 0 |
| Strength-endurance | 0 | 0 | 9 | 0 | 0 | 0 | 0 | 0 |
| Skill | 0 | 0 | 0 | 0 | 0 | 0 | 0 | 13 |
| **Exercise category** | **Time of day exercise task was performed** | | | | |  |  |  |
|  | **0001-0600 (n)** | **0601-1200 (n)** | **1201-1800 (n)** | **1801-2400 (n)** | **Not reported (n)** |  |  |  |
| All categories | 10 | 107 | 96 | 8 | 6 |  |  |  |
| Anaerobic power | 2 | 25 | 29 | 1 | 1 |  |  |  |
| Speed/power endurance | 1 | 13 | 15 | 0 | 3 |  |  |  |
| HIIE | 0 | 11 | 16 | 0 | 0 |  |  |  |
| Strength | 7 | 32 | 25 | 1 | 1 |  |  |  |
| Endurance | 0 | 12 | 5 | 3 | 1 |  |  |  |
| Strength-endurance | 0 | 8 | 0 | 1 | 0 |  |  |  |
| Skill | 0 | 5 | 6 | 2 | 0 |  |  |  |

**Abbreviations/definitions:** HIIE: high-intensity interval exercise; n: number of outcome measures; time of exercise task was performed: tasks were sub-categorised into 6 h blocks based on what time of the day the task was undertaken; Not reported: no time of day specified when task was performed.

| **Exercise category** | **# Participants** | **# male** | **# female** | **Age (y)** | **Height (cm)** | **BM (kg)** | $\dot{\boldsymbol{V}}$**O_2peak_** |
| --- | --- | --- | --- | --- | --- | --- | --- |
| All categories | 959 | 855 | 104 | 22.6 ± 3.47 | 176 ± 5.10 | 73.1 ± 9.26 | 53.4 ± 9.29 |
| Anaerobic power | 368 | 339 | 29 | 21.6 ± 2.14 | 177 ± 4.37 | 73.4 ± 6.86 | 52.1 ± 12.4 |
| Speed/power endurance | 261 | 254 | 7 | 21.4 ± 1.66 | 177 ± 3.45 | 73.7 ± 5.94 | 69.3^*^ |
| HIIE | 207 | 184 | 23 | 20.8 ± 2.69 | 175 ± 5.55 | 69.1 ± 7.31 | 54.9 ± 2.76 |
| Endurance | 237 | 216 | 21 | 25.1 ± 3.89 | 176 ± 5.90 | 733 ± 11.0 | 52.2 ± 9.55 |
| Strength | 289 | 253 | 36 | 23.3 ± 3.93 | 173 ± 7.14 | 73.7 ± 9.68 | 54.3 ± 7.11 |
| Strength-endurance | 62 | 62 | 0 | 22.4 ± 3.58 | 181 ± 3.96 | 91.1 ± 12.2 | 44.8^#^ |
| Skill | 146 | 116 | 30 | 22.4 ± 3.76 | 180 ± 4.58 | 82.0 ± 8.00 | NS |

**Supplementary Table S5 continued**: Participant characteristics (mean ± SD)

**Abbreviations:** HIIE: high-intensity interval exercise; BM: body mass; kg: kilogram; y: year; cm: centimetre; $\dot{V}$O_2peak_: peak oxygen consumption; #: number; NS: Not specified. ^*^value from one study (Mougin et al. 1996) [93]; ^#^ value from one study (Rodrigues al. 2021 [101] )

**Supplementary Table S6:** Study quality assessment

| **Citation** | A clear description of the inclusion and exclusion criteria was provided | The trials were randomized | Treatment order was counterbalanced | The method used to generate the random allocation sequence was described | Sample size was justified | Attempts were made to control and/or monitor pre-trial conditions | Design incorporated measures of important baseline variables | Subjects were blinded | Investigators were blinded | Methods and successfulness of blinding were described | Details were provided regarding the inability of a subject to complete study requirements | Statistical methods described | Primary outcome measurement and variability reported | Results of statistical comparisons reported | Methods used to assess adverse effects described | Reproducibility of the primary outcome measure(s) was reported | A familiarization of the performance test was conducted | **Total Score (%)** |
| --- | --- | --- | --- | --- | --- | --- | --- | --- | --- | --- | --- | --- | --- | --- | --- | --- | --- | --- |
| **Studies investigating the influence of acute sleep loss on physical performance** | | | | | | | | | | | | | | | | | | |
| Abedelmalek et al. 2014 [116] | 1 | 1 | 0 | 0 | 0 | 1 | 1 | NA | NA | NA | NA | 1 | 1 | 1 | NA | 0 | 0 | 58 |
| Abedelmalek et al. 2013 [62] | 1 | 1 | 0 | 0 | 0 | 1 | 1 | NA | NA | NA | NA | 1 | 1 | 1 | NA | 0 | 0 | 58 |
| Ajjimaporn et al. 2020 [56] | 1 | 1 | 0 | 1 | 1 | 1 | 1 | NA | NA | NA | NA | 1 | 1 | 1 | NA | 0 | 0 | 75 |
| Arazi et al. 2019 [85] | 1 | 0 | 0 | 0 | 0 | 1 | 1 | NA | NA | NA | NA | 1 | 1 | 1 | NA | 0 | 0 | 50 |
| Arnal et al. 2016 [86] | 1 | 1 | 1 | 0 | 0 | 1 | 1 | NA | NA | NA | NA | 1 | 1 | 1 | NA | 0 | 1 | 75 |
| Azboy et al. 2009 [120] | 1 | 0 | 0 | 0 | 0 | 1 | 1 | NA | NA | NA | NA | 1 | 1 | 1 | NA | 0 | 1 | 58 |
| Baati et al. 2020 [87] | 1 | 1 | 0 | 0 | 0 | 0 | 1 | NA | NA | NA | NA | 1 | 1 | 1 | NA | 0 | 1 | 58 |
| Baati et al. 2015 [88] | 1 | 1 | 0 | 0 | 0 | 0 | 1 | NA | NA | NA | NA | 1 | 1 | 1 | NA | 0 | 1 | 58 |
| Bambaeichi et al. 2005 [53] | 1 | 1 | 1 | 0 | 0 | 1 | 1 | NA | NA | NA | NA | 1 | 1 | 1 | NA | 0 | 1 | 75 |
| Blumert et al. 2007 [75] | 1 | 1 | 1 | 0 | 0 | 1 | 1 | NA | NA | NA | NA | 1 | 1 | 1 | NA | 1 | 1 | 83 |
| Brodan and Kuhn 1967 [130] | 1 | 0 | 0 | NA | 0 | 1 | 1 | NA | NA | NA | NA | 1 | 1 | 1 | NA | 0 | 1 | 64 |
| Bulbulian et al. 1996 [83] | 1 | 0 | 0 | NA | 0 | 0 | 1 | NA | NA | NA | NA | 1 | 1 | 1 | NA | 0 | 0 | 45 |
| Chase et al. 2017 [41] | 1 | 1 | 1 | 0 | 0 | 1 | 1 | NA | NA | NA | 1 | 1 | 1 | 1 | NA | 1 | 1 | 85 |
| Chen 1991 [126] | 1 | 1 | 0 | 1 | 0 | 1 | 1 | NA | NA | NA | NA | 1 | 1 | 1 | NA | **0** | 1 | 75 |
| Cook et al. 2012 [84] | 1 | 1 | 1 | 0 | 0 | 1 | 1 | NA | NA | NA | NA | 1 | 1 | 1 | NA | 0 | 1 | 75 |
| Cook et al. 2011 [63] | 1 | 1 | 0 | 0 | 0 | 1 | 1 | NA | NA | NA | NA | 1 | 1 | 1 | NA | 1 | 1 | 75 |
| Cullen et al. 2019 [57] | 1 | 1 | 1 | 0 | 0 | 1 | 1 | NA | NA | NA | NA | 1 | 1 | **1** | NA | 0 | 1 | 75 |
| Daaloul et al. 2019 [58] | 1 | 1 | 0 | 0 | 0 | 1 | 1 | NA | NA | NA | NA | 1 | 1 | 1 | NA | 0 | 1 | 67 |
| Dáttilo et al. 2020 [127] | 1 | 1 | 0 | 0 | 0 | 1 | 1 | NA | NA | NA | NA | 1 | 1 | 1 | NA | 0 | 1 | 67 |
| Daviaux et al. 2014 [100] | 1 | 1 | NA | 0 | 0 | 0 | 1 | NA | NA | NA | NA | 1 | 1 | 1 | NA | 0 | 0 | 55 |
| Edwards and Waterhouse 2009 [45] | 0 | 1 | 0 | 0 | 0 | 1 | 1 | NA | NA | NA | NA | 1 | 1 | 1 | NA | 1 | 1 | 67 |
| Filipas et al.2021 [109] | 1 | 1 | 1 | 1 | 0 | 1 | 1 | NA | NA | NA | NA | 1 | 1 | 1 | NA | 1 | 1 | 92 |
| Froberg et al. 1975 [121] | 1 | 0 | 0 | NA | 0 | 1 | 1 | NA | NA | NA | 1 | 1 | 1 | 1 | NA | 0 | 1 | 67 |
| Goh et al. 2001 [55] | 1 | 1 | NA | 0 | 0 | 1 | 1 | NA | NA | NA | NA | 1 | 1 | 1 | NA | 0 | 0 | 64 |
| Goncalves et al. 2020 [76] | 1 | 1 | 1 | 0 | 1 | 1 | 1 | NA | NA | NA | NA | 1 | 1 | 1 | NA | 0 | 1 | 83 |

**Supplementary Table S6:** continued

| **Citation** | A clear description of the inclusion and exclusion criteria was provided | The trials were randomized | Treatment order was counterbalanced | The method used to generate the random allocation sequence was described | Sample size was justified | Attempts were made to control and/or monitor pre-trial conditions | Design incorporated measures of important baseline variables | Subjects were blinded | Investigators were blinded | Methods and successfulness of blinding were described | Details were provided regarding the inability of a subject to complete study requirements | Statistical methods described | Primary outcome measurement and variability reported | Results of statistical comparisons reported | Methods used to assess adverse effects described | Reproducibility of the primary outcome measure(s) was reported | A familiarization of the performance test was conducted | **Total Score (%)** |
| --- | --- | --- | --- | --- | --- | --- | --- | --- | --- | --- | --- | --- | --- | --- | --- | --- | --- | --- |
| **Studies investigating the influence of acute sleep loss on physical performance** | | | | | | | | | | | | | | | | | | |
| Goto et al. 2020 [54] | 1 | 1 | 0 | 0 | 0 | 1 | 1 | NA | NA | NA | NA | 1 | 1 | 1 | NA | 0 | 1 | 67 |
| HajSalem et al. 2013 [89] | 1 | 1 | 0 | 0 | 0 | 0 | 1 | NA | NA | NA | NA | 1 | 1 | 1 | NA | 0 | 1 | 58 |
| Hill et al. 1994 [122] | 0 | 0 | 0 | NA | 0 | 1 | 1 | NA | NA | NA | NA | 1 | 1 | 1 | NA | 1 | 1 | 64 |
| Holland 1968 [111] | 1 | 0 | 0 | NA | 0 | 1 | 1 | NA | NA | NA | NA | 0 | 1 | 1 | NA | 0 | 1 | 55 |
| Khcharem et al. 2020 [131] | 1 | 1 | 0 | 0 | 0 | 1 | 1 | NA | NA | NA | NA | 1 | 1 | 1 | NA | 0 | 1 | 67 |
| Khemila et al. 2021 [43] | 1 | 1 | 0 | 0 | 1 | 1 | 1 | NA | NA | NA | NA | 1 | 1 | 1 | NA | 0 | 1 | 75 |
| Kujawa et al. 2020 [82] | 1 | 1 | NA | 0 | 0 | 1 | 1 | NA | NA | NA | NA | 1 | 1 | 1 | NA | 0 | 1 | 73 |
| Martin et al. 1981 [132] | 0 | 0 | 1 | 0 | 0 | 1 | 1 | NA | NA | NA | NA | 1 | 1 | 1 | NA | 0 | 1 | 58 |
| Mejri et al. 2014 [139] | 1 | 1 | 1 | 0 | 0 | 1 | 1 | NA | NA | NA | NA | 1 | **1** | 0 | NA | 0 | 1 | 67 |
| Mejri et al. 2016 [124] | 1 | 1 | 1 | 0 | 0 | 1 | 1 | NA | NA | NA | NA | 1 | 1 | 1 | NA | 0 | 1 | 75 |
| Moore et al. 2018 [59] | 1 | 0 | 0 | NA | 0 | 1 | 1 | NA | NA | NA | 1 | 1 | 1 | 1 | NA | 0 | 1 | 67 |
| Mougin 1991 [49] | 1 | 0 | 0 | NA | 0 | 1 | 1 | NA | NA | NA | NA | 1 | 1 | 1 | NA | 0 | 0 | 55 |
| Mougin et al. 2001 [67] | 1 | 0 | 0 | NA | 0 | 0 | 1 | NA | NA | NA | NA | 1 | 1 | 1 | NA | 0 | 1 | 55 |
| Mougin et al. 1996 [93] | 1 | 1 | 0 | 0 | 0 | 1 | 1 | NA | NA | NA | NA | 1 | 1 | 1 | NA | 0 | 1 | 67 |
| Oliver et al. 2009 [110] | 0 | 1 | 0 | 0 | 0 | 1 | 1 | NA | NA | NA | NA | 1 | 1 | 1 | NA | 1 | 1 | 67 |
| Omiya et al. 2009 [128] | 1 | 0 | 0 | NA | 0 | 1 | 1 | NA | NA | NA | NA | 1 | 1 | 1 | NA | 0 | 0 | 55 |
| Ozturk et al. 2007 [94] | 1 | 0 | 0 | NA | 0 | 1 | 1 | NA | NA | NA | NA | 1 | 1 | 1 | NA | 0 | 1 | 64 |
| Paryab et al. 2021 [125] | 1 | 1 | 0 | 1 | 0 | 1 | 1 | 1 | 1 | 0 | NA | 1 | 1 | 1 | NA | 0 | 0 | 67 |
| Racinais et al. 2004 [129] | **1** | 0 | 0 | NA | 0 | 1 | 1 | NA | NA | NA | NA | 1 | 1 | 1 | NA | 0 | 0 | 55 |
| Rae et al. 2017 [50] | 1 | 1 | 0 | 0 | 1 | 1 | 1 | NA | NA | NA | NA | 1 | 1 | 1 | NA | 0 | 1 | 75 |
| Reilly and Piercy 1994 [40] | 1 | 0 | 1 | NA | 0 | 1 | 1 | NA | NA | NA | NA | 1 | 1 | 1 | NA | 0 | 1 | 73 |
| Reyner and Horne 2013 [123] | 1 | 0 | 1 | NA | 0 | 1 | 1 | NA | NA | NA | NA | 1 | 1 | 1 | NA | 0 | 0 | 64 |
| Roberts et al. 2019 [51] | 1 | 0 | 1 | NA | 0 | 1 | 1 | NA | NA | NA | NA | 1 | 1 | 1 | NA | 0 | 1 | 73 |
| Roberts et al. 2019 [52] | 1 | 1 | 1 | 0 | 0 | 1 | 1 | NA | NA | NA | NA | 1 | 1 | 1 | NA | 0 | 1 | 75 |
| Rodrigues et al. 2021 [101] | 1 | 1 | 0 | 0 | 0 | 1 | 1 | 0 | 1 | 0 | NA | 1 | 1 | 1 | NA | 0 | 1 | 60å |
| Romdhani et al. 2020 [95] | 1 | 1 | 1 | 0 | 0 | 1 | 1 | NA | NA | NA | 1 | 1 | 1 | 1 | NA | 0 | 1 | 77 |
| Romdhani et al. 2019 [44] | 1 | 1 | 1 | 0 | 0 | 1 | 1 | NA | NA | NA | NA | 1 | 1 | 1 | NA | 0 | 1 | 75 |

**Supplementary Table S6:** continued

| **Citation** | A clear description of the inclusion and exclusion criteria was provided | The trials were randomized | Treatment order was counterbalanced | The method used to generate the random allocation sequence was described | Sample size was justified | Attempts were made to control and/or monitor pre-trial conditions | Design incorporated measures of important baseline variables | Subjects were blinded | Investigators were blinded | Methods and successfulness of blinding were described | Details were provided regarding the inability of a subject to complete study requirements | Statistical methods described | Primary outcome measurement and variability reported | Results of statistical comparisons reported | Methods used to assess adverse effects described | Reproducibility of the primary outcome measure(s) was reported | A familiarization of the performance test was conducted | **Total Score (%)** |
| --- | --- | --- | --- | --- | --- | --- | --- | --- | --- | --- | --- | --- | --- | --- | --- | --- | --- | --- |
| **Studies investigating the influence of acute sleep loss on physical performance** | | | | | | | | | | | | | | | | | | |
| Romdhani et al. 2021 [90] | 1 | 1 | 0 | 0 | 0 | 1 | 1 | NA | NA | NA | NA | 1 | 1 | 1 | NA | 0 | 1 | 67 |
| Romdhani et al. 2021 [102] | 1 | 1 | 1 | 0 | 0 | 1 | 1 | NA | NA | NA | 0 | 1 | 1 | 1 | NA | 0 | 1 | 69 |
| Skein et al. 2011 [91] | 1 | 0 | 1 | NA | 0 | 1 | 1 | NA | NA | NA | NA | 1 | 1 | 1 | NA | 1 | 1 | 82 |
| Skein et al. 2013 [96] | 1 | 1 | 0 | 0 | 0 | 1 | 1 | NA | NA | NA | NA | 1 | 1 | 1 | NA | 0 | 0 | 58 |
| Skurvydas et al. 2020 [60] | 1 | 1 | 0 | 1 | 0 | 1 | 1 | NA | NA | NA | NA | 1 | 1 | 1 | NA | 0 | 1 | 75 |
| Souissi et al. 2014 [78] | 1 | 1 | 0 | 0 | 0 | 1 | 1 | NA | NA | NA | NA | 1 | 1 | 1 | NA | 0 | 0 | 58 |
| Souissi et al. 2019 [79] | 1 | 1 | 0 | 0 | 0 | 1 | 1 | NA | NA | NA | NA | 1 | 1 | 1 | NA | 0 | 1 | 67 |
| Souissi et al. 2003 [80] | 1 | 1 | 0 | 0 | 0 | 1 | 1 | NA | NA | NA | NA | 1 | 1 | 1 | NA | 0 | 0 | 58 |
| Souissi et al. 2008 [81] | 1 | 1 | 0 | 0 | 0 | 1 | 1 | NA | NA | NA | NA | 1 | 1 | 1 | NA | 0 | 1 | 67 |
| Souissi et al. 2015 [77] | 1 | 0 | 0 | NA | 0 | 1 | 1 | NA | NA | NA | NA | 1 | 1 | 1 | NA | 0 | 0 | 55 |
| Souissi et al. 2018 [97] | 1 | 0 | 0 | NA | 0 | 1 | 1 | NA | NA | NA | NA | 1 | 1 | 1 | NA | 1 | 0 | 64 |
| Souissi et al. 2020 [98] | 1 | 0 | 1 | NA | 0 | 1 | 1 | NA | NA | NA | 1 | 1 | 1 | 1 | NA | 0 | 1 | 75 |
| Souissi et al. 2013 [64] | 1 | 1 | 0 | 0 | 0 | 1 | 1 | NA | NA | NA | NA | 1 | 1 | 1 | NA | 1 | 1 | 75 |
| Sweeney et al. 2020 [61] | 1 | 1 | 0 | 0 | 1 | 1 | 1 | NA | NA | NA | NA | 1 | 1 | 1 | NA | 0 | 1 | 75 |
| Taheri and Arabameri 2012 [99] | 1 | 0 | 0 | NA | 0 | 1 | 1 | NA | NA | NA | NA | 1 | 1 | 1 | NA | 0 | 0 | 55 |
| Temesi et al. 2013 [92] | 1 | 1 | 1 | 0 | 0 | 1 | 1 | NA | NA | NA | NA | 1 | 1 | 1 | NA | 0 | 1 | 75 |
| Vardar et al. 2007 [65] | 1 | 0 | 0 | NA | 1 | 1 | 1 | NA | NA | NA | NA | 1 | 1 | 1 | NA | 0 | 1 | 73 |

**
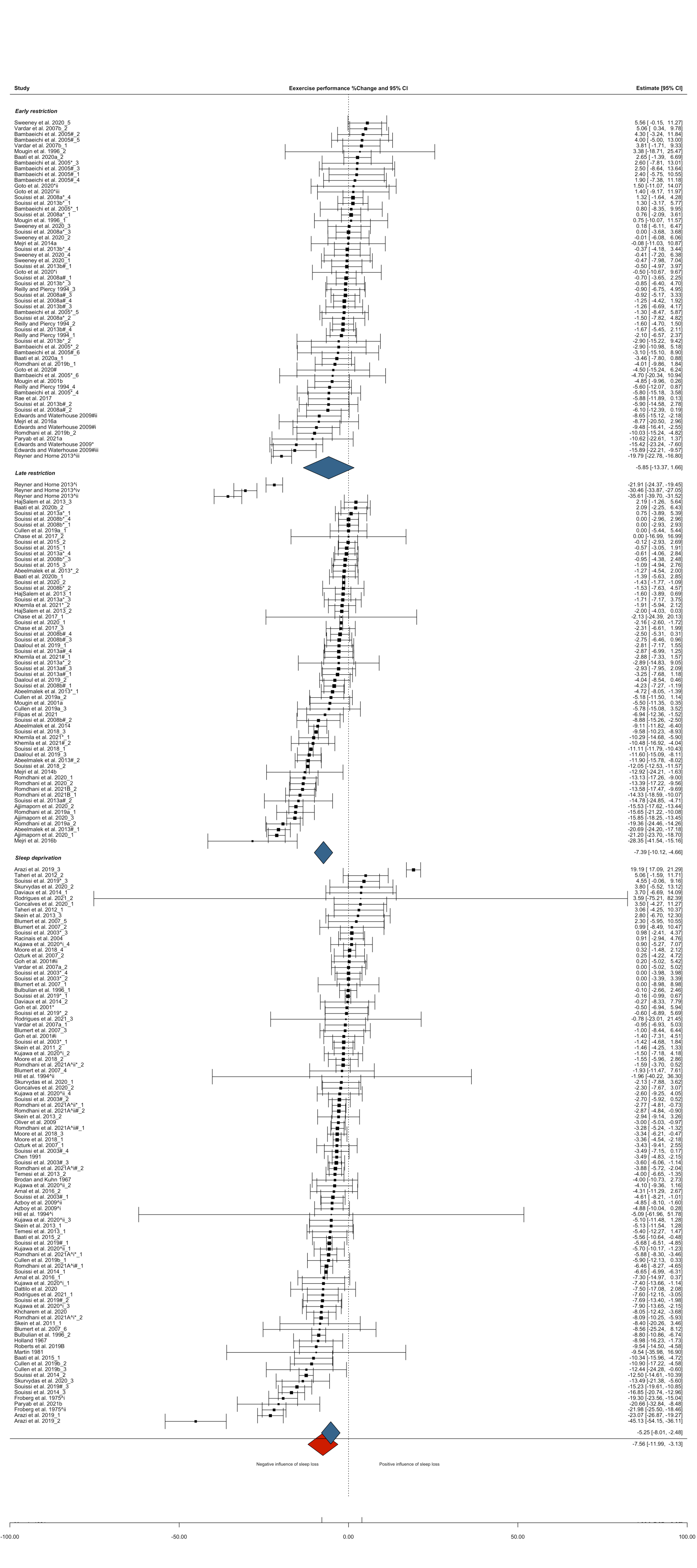
**

**Supplementary Figure S1:** Forest plot displaying the effect of sleep loss vs. normal sleep on the percentage change in exercise performance. The size of the squares are proportional to the weight of the study. A negative effect estimate indicates a decrease in exercise performance under the intervention condition (‘sleep loss’). Note, trials from Mougin 1991 [49] (fragmented sleep), Omiya et al. 2009 [128], Cook et al. 2011 [63], Cook et al. 2012 [84] and Roberts et al. 2019 [51] (type of sleep restriction protocol not stated) are included in overall effect estimate. Individual participant pools from a single study are denoted by the addition of a caret followed by a roman numeral (i.e., ^i, ^ii, ^iii etc.) to the citation; different sleep loss protocols (i.e., deprivation and late-restriction or early-restriction) from a single research study are denoted by the addition of a lower-case letter (i.e., a, b) to the citation; * or # indicates task performed in the AM or PM, respectively. * or # followed by a roman numeral value (i.e., *i, *ii, *iii) indicates that the same task was performed at multiple times during AM or PM; underscore numerical value (i.e., _1, _2, _3 etc.) identifies the different tasks performed at a given time; studies conducted by the same lead author in the same year are denoted by the addition of a capital ‘a or ‘b’ (i.e., A, B) to the citation. Deprivation: participants did not sleep for an extended period of time (i.e., whole night); restriction: total sleep time ≤6 h in any 24 h period – this category is a combination of early-restriction, late-restriction, fragmented sleep and sleep restriction protocols not specified; Early restriction: participants delayed sleep (i.e., went to sleep at a later time); Late restriction; participants awakened earlier than normal.

**Supplementary Table S7**: Sensitivity analysis of alternative levels of correlation coefficient (R) on overall exercise performance meta-analysis results

|  |  |  | **Exercise performance % change** |  | **Heterogeneity** |  |  |  |  | **Sensitivity analysis – one out** | |
| --- | --- | --- | --- | --- | --- | --- | --- | --- | --- | --- | --- |
|  | **Outcomes, n** | **R** | **Mean (95% CI)** | ***p*** | **I^2^-value** | ***p*** | **σ^2^ effect** | **σ^2^mode** | **σ^2^study** | **Range (EP%_Δ_)** | **CI crossed (y or n)** |
| **All tasks** |  |  |  |  |  |  |  |  |  |  |  |
| Overall | 227 | 0.71 | -7.56 (-11.9 to -3.13) | 0.001 | 98.1 | <0.001 | 21.2 | 32.1 | 14.5 | -7.91 to -7.28 | n |
|  |  | 0.80 | -7.56 (-11.9 to -3.18) | 0.001 |  |  |  |  |  |  |  |
|  |  | 0.30 | -7.69 (-12.3 to -3.07) | 0.001 |  |  |  |  |  |  |  |
| **Sleep condition** |  |  |  |  |  |  |  |  |  |  |  |
| Deprivation | 97 | 0.71 | -5.25 (-8.01 to -2.48) | <0.001 | 96.5 | <0.001 | 37.5 | 8.53 | <0.001 | -5.64 to -4.34 | n |
|  |  | 0.80 | -5.19 (-7.77 to -2.61) | 0.001 |  |  |  |  |  |  |  |
|  |  | 0.30 | -5.52 (-8.84 to -2.20) | 0.001 |  |  |  |  |  |  |  |
| Restriction | 130 | 0.71 | -8.59 (-13.6 to -3.61) | 0.001 | 98.3 | <0.001 | 7.43 | 35.1 | 34.0 | -8.96 to -8.12 | n |
|  |  | 0.80 | -8.57 (-13.5 to -3.63) | 0.001 |  |  |  |  |  |  |  |
|  |  | 0.30 | -8.68 (-13.8 to -3.55) | 0.001 |  |  |  |  |  |  |  |
| *Early restriction* | 62 | 0.71 | -5.85 (-13.4 to 1.66) | 0.125 | 93.5 | <0.001 | <0.001 | 73.5 | 28.0 | -6.12 to -5.20 | y |
|  |  | 0.80 | -5.90 (-13.4 to 1.57) | 0.119 |  |  |  |  |  |  |  |
|  |  | 0.30 | -5.63 (-13.3 to 2.06) | 0.148 |  |  |  |  |  |  |  |
| *Late restriction* | 60 | 0.71 | -7.39 (-10.1 to -4.66) | <0.001 | 98.3 | <0.001 | 9.65 | <0.001 | 30.8 | -7.57 to -6.91 | n |
|  |  | 0.80 | -7.47 (-10.2 to -4.71) | <0.001 |  |  |  |  |  |  |  |
|  |  | 0.30 | -7.15 (-9.84 to -4.46) | <0.001 |  |  |  |  |  |  |  |
| **AM vs. PM** |  |  |  |  |  |  |  |  |  |  |  |
| Overall AM | 115 | 0.71 | -5.42 (-9.66 to -1.17) | 0.013 | 93.5 | <0.001 | 27.2 | 28.1 | 2.18 | -5.77 to -5.04 | n |
|  |  | 0.80 | -5.40 (-9.53 to -1.27) | 0.011 |  |  |  |  |  |  |  |
|  |  | 0.30 | -5.58 (-10.3 to -0.82) | 0.022 |  |  |  |  |  |  |  |
| Overall PM | 106 | 0.71 | -8.31 (-13..2 to -3.37) | 0.001 | 98.9 | <0.001 | 7.06 | 35.8 | 26.2 | -8.72 to -7.98 | n |
|  |  | 0.80 | -8.33 (-13.2 to -3.44) | 0.001 |  |  |  |  |  |  |  |
|  |  | 0.30 | -8.31 (-13.4 to -3.22) | 0.002 |  |  |  |  |  |  |  |

**Supplementary Table S7**: continued

|  |  |  | **Exercise performance % change** |  | **Heterogeneity** |  |  |  |  | **Sensitivity analysis – one out** | |
| --- | --- | --- | --- | --- | --- | --- | --- | --- | --- | --- | --- |
|  | **Outcomes, n** | **R** | **Mean (95% CI)** | ***p*** | **I^2^-value** | ***p*** | **σ^2^ effect** | **σ^2^mode** | **σ^2^study** | **Range (EP%_Δ_)** | **CI crossed (y or n)** |
| **Sleep condition** |  |  |  |  |  |  |  |  |  |  |  |
| Deprivation AM | 59 | 0.71 | -3.48 (-5.89 to -1.08) | 0.005 | 94.2 | <0.001 | 50.6 | 1.77 | <0.001 | -4.91 to -2.83 | n |
|  |  | 0.80 | -3.54 (-5.91 to -1.17) | 0.004 |  |  |  |  |  |  |  |
|  |  | 0.30 | -3.31 (-5.83 to -0.79) | 0.011 |  |  |  |  |  |  |  |
| Deprivation PM | 35 | 0.71 | -6.85 (-11.3 to -2.39) | 0.004 | 97.5 | <0.001 | 8.34 | 26.5 | 6.20 | -71.9 to -5.33 | n |
|  |  | 0.80 | -6.89 (-11.3 to -2.44) | 0.003 |  |  |  |  |  |  |  |
|  |  | 0.30 | -6.96 (-11.4 to -2.49) | 0.003 |  |  |  |  |  |  |  |
| Restriction AM | 56 | 0.71 | -5.96 (-11.5 to -0.43) | 0.035 | 90.7 | <0.001 | <0.001 | 48.2 | 7.66 | -6.45 to -5.67 | n |
|  |  | 0.80 | -5.96 (-11.4 to -0.51) | 0.033 |  |  |  |  |  |  |  |
|  |  | 0.30 | -6.02 (-11.9 to 0.11) | 0.046 |  |  |  |  |  |  |  |
| Restriction PM | 71 | 0.71 | -9.50 (-14.9 to -4.12) | 0.001 | 99.0 | <0.001 | 5.53 | 32.1 | 45.4 | -9.69 to -8.83 | n |
|  |  | 0.80 | -9.50 (-14.9 to -4.15) | 0.001 |  |  |  |  |  |  |  |
|  |  | 0.30 | -9.51 (-14.9 to -4.04) | 0.001 |  |  |  |  |  |  |  |
| *Early restriction AM* | 27 | 0.71 | -1.55 (-4.66 to 1.56) | 0.315 | 55.1 | 0.437 | <0.001 | <0.001 | 12.0 | -1.79 to -0.26 | y |
|  |  | 0.80 | -2.24 (-6.17 to 1.69) | 0.252 |  |  |  |  |  |  |  |
|  |  | 0.30 | -0.23 (-2.08 to 1.62) | 0.800 |  |  |  |  |  |  |  |
| *Early restriction PM* | 35 | 0.71 | -6.23 (-13.9 to 1.44) | 0.108 | 94.7 | <0.001 | <0.001 | 67.7 | 36.8 | -6.93 to -5.57 | y |
|  |  | 0.80 | -6.25 (-13.9 to 1.35) | 0.104 |  |  |  |  |  |  |  |
|  |  | 0.30 | -6.12 (-14.0 to 1.73) | 0.122 |  |  |  |  |  |  |  |
| *Late restriction AM* | 23 | 0.71 | -2.48 (-4.36 to -0.60) | 0.012 | 46.1 | 0.048 | <0.001 | 0.51 | 3.42 | -2.97 to -1.71 | n |
|  |  | 0.80 | -2.57 (-4.46 to -0.68) | 0.010 |  |  |  |  |  |  |  |
|  |  | 0.30 | -2.18 (-3.92 to -0.44) | 0.016 |  |  |  |  |  |  |  |
| *Late restriction PM* | 34 | 0.71 | -9.67 (-13.1 to -6.24) | <0.001 | 99.1 | <0.001 | 5.43 | <0.001 | 36.5 | -9.97 to -9.04 | n |
|  |  | 0.80 | -9.77 (-13.3 to -6.28) | <0.001 |  |  |  |  |  |  |  |
|  |  | 0.30 | -9.39 (-12.7 to -6.06) | <0.001 |  |  |  |  |  |  |  |

**Abbreviations/definitions**: AM: ante meridiem; PM: post meridiem; Deprivation: participants did not sleep for an extended period of time (i.e., whole night); restriction: total sleep time ≤6 h in any 24 h period – this category is a combination of early-restriction, late-restriction, fragmented sleep and sleep restriction protocols not specified; Early restriction: participants delayed sleep (i.e., went to sleep at a later time); Late restriction; participants awakened earlier than normal.

**
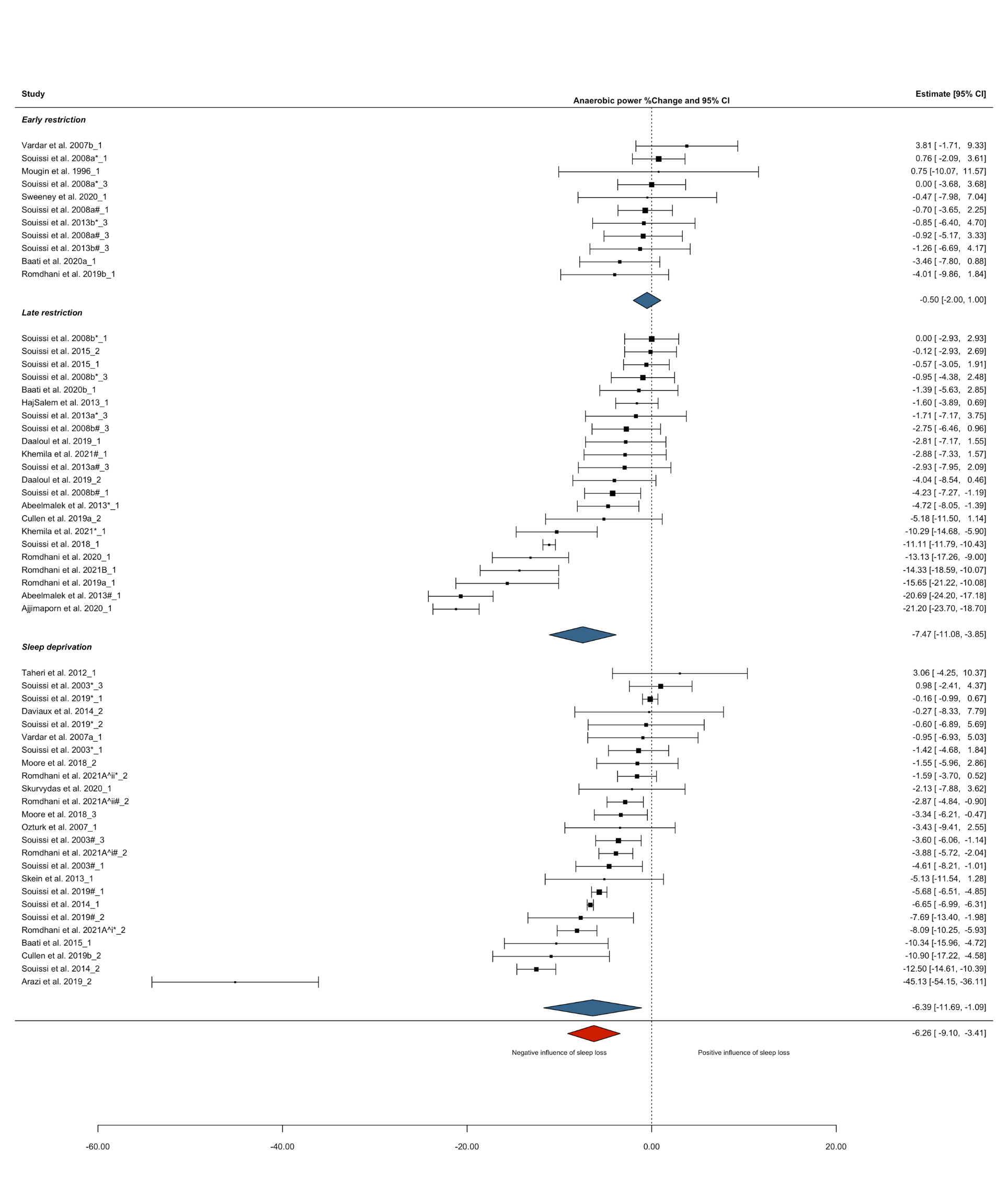
Supplementary Figure S2:** Forest plot displaying the effect of sleep loss vs. normal sleep on the percentage change in anaerobic power performance. The size of the squares are proportional to the weight of the study. A negative effect estimate indicates a decrease in anaerobic power performance under the intervention condition (‘sleep loss’). Individual participant pools from a single study are denoted by the addition of a caret followed by a roman numeral (i.e., ^i, ^ii, ^iii etc.) to the citation; different sleep loss protocols (i.e., deprivation and late-restriction or early-restriction) from a single research study are denoted by the addition of a lower-case letter (i.e., a, b) to the citation; * or # indicates task performed in the AM or PM, respectively. * or # followed by a roman numeral value (i.e., *i, *ii, *iii) indicates that the same task was performed at multiple times during AM or PM; underscore numerical value (i.e., _1, _2, _3 etc.) identifies the different tasks performed at a given time. Deprivation: participants did not sleep for an extended period of time (i.e., whole night); restriction: total sleep time ≤6 h in any 24 h period – this category is a combination of early-restriction, late-restriction, fragmented sleep and sleep restriction protocols not specified; Early restriction: participants delayed sleep (i.e., went to sleep at a later time); Late restriction; participants awakened earlier than normal.

**Supplementary Table S8**: Sensitivity analysis of alternative levels of correlation coefficient (R) on anaerobic power performance meta-analysis results

|  |  |  | **Exercise performance** |  | **Heterogeneity** |  |  |  | **Sensitivity analysis – one out** | |
| --- | --- | --- | --- | --- | --- | --- | --- | --- | --- | --- |
|  |  |  | **% change** |  |  |  |  |  |  |  |
| **Exercise category** | **Outcomes, n** | **R** | **Mean (95% CI)** | ***p*** | **I^2^-value** | ***p*** | **σ^2^effect** | **σ^2^study** | **Range (EP%_Δ_)** | **CI crossed (y or n)** |
| **Anaerobic power** |  |  |  |  |  |  |  |  |  |  |
| Overall | 58 | 0.71 | -6.26 (-9.10 to -3.41) | <0.001 | 98.1 | <0.001 | 9.47 | 49.2 | -6.59 to -5.24 | n |
|  |  | 0.80 | -6.27 (-9.15 to -3.38) | <0.001 |  |  |  |  |  |  |
|  |  | 0.30 | -6.18 (-8.72 to -3.64) | <0.001 |  |  |  |  |  |  |
| **Sleep condition** |  |  |  |  |  |  |  |  |  |  |
| Deprivation | 25 | 0.71 | -6.39 (-11.7 to -1.09) | 0.020 | 99.2 | <0.001 | 7.10 | 86.7 | -7.01 to -4.16 | n |
|  |  | 0.80 | -6.43 (-11.8 to -1.06) | 0.021 |  |  |  |  |  |  |
|  |  | 0.30 | -6.07 (-10.5 to -1.61) | 0.009 |  |  |  |  |  |  |
| Restriction | 33 | 0.71 | -5.99 (-9.22 to -2.77) | 0.001 | 94.1 | <0.001 | 11.7 | 30.2 | -6.61 to -5.05 | n |
|  |  | 0.80 | -5.95 (-9.17 to -2.74) | 0.001 |  |  |  |  |  |  |
|  |  | 0.30 | -6.18 (-9.46 to -2.89) | 0.001 |  |  |  |  |  |  |
| *Early restriction* | 11 | 0.71 | -0.50 (-2.00 to 1.00) | 0.477 | 0.04 | 0.770 | 0.001 | 0.001 | -0.92 to -0.20 | y |
|  |  | 0.80 | -0.62 (-2.29 to 1.05) | 0.428 |  |  |  |  |  |  |
|  |  | 0.30 | -0.52 (-2.80 to 1.76) | 0.620 |  |  |  |  |  |  |
| *Late restriction* | 22 | 0.71 | -7.47 (-11.1 to -3.85) | <0.001 | 95.4 | <0.001 | 18.9 | 25.6 | -8.42 to -6.36 | n |
|  |  | 0.80 | -7.49 (-11.1 to -3.88) | <0.001 |  |  |  |  |  |  |
|  |  | 0.30 | -7.40 (-11.1 to -3.75) | <0.001 |  |  |  |  |  |  |
|  |  |  |  |  |  |  |  |  |  |  |
| **Am vs. PM** |  |  |  |  |  |  |  |  |  |  |
| Overall AM | 27 | 0.71 | -4.58 (-9.14 to -0.24) | 0.049 | 97.0 | <0.001 | <0.001 | 81.3 | -5.00 to -2.65 | y |
|  |  | 0.80 | -4.61 (-9.24 to 0.03) | 0.052 |  |  |  |  |  |  |
|  |  | 0.30 | -4.28 (-8.09 to -0.47) | 0.029 |  |  |  |  |  |  |
| Overall PM | 30 | 0.71 | -7.37 (-10.3 to -4.40) | <0.001 | 97.8 | <0.001 | 5.13 | 33.3 | -7.64 to -6.56 | n |
|  |  | 0.80 | -7.36 (-10.3 to -4.42) | <0.001 |  |  |  |  |  |  |
|  |  | 0.30 | -7.42 (-10.4 to -4.44) | <0.001 |  |  |  |  |  |  |

**Supplementary Table S8**: continued

|  |  |  | **Exercise performance** |  | **Heterogeneity** |  |  |  | **Sensitivity analysis – one out** | |
| --- | --- | --- | --- | --- | --- | --- | --- | --- | --- | --- |
|  |  |  | **% change** |  |  |  |  |  |  |  |
| **Exercise category** | **Outcomes, n** | **R** | **Mean (95% CI)** | ***p*** | **I^2^-value** | ***p*** | **σ^2^effect** | **σ^2^study** | **Range (EP%_Δ_)** | **CI crossed (y or n)** |
| **Sleep condition** |  |  |  |  |  |  |  |  |  |  |
| Deprivation AM | 14 | 0.71 | -6.38 (-14.5 to 1.73) | 0.113 | 98.5 | <0.001 | <0.001 | 147 | -7.30 to -2.83 | y |
|  |  | 0.80 | -6.41 (-14.6 to 1.77) | 0.114 |  |  |  |  |  |  |
|  |  | 0.30 | -6.07 (-13.6 to 1.44) | 0.105 |  |  |  |  |  |  |
| Deprivation PM | 11 | 0.71 | -5.49 (-7.93 to -3.04) | <0.001 | 93.5 | <0.001 | 5.37 | 3.14 | -5.89 to -4.86 | n |
|  |  | 0.80 | -5.57 (-8.04 to -3.10) | <0.001 |  |  |  |  |  |  |
|  |  | 0.30 | -5.80 (-8.39 to -3.21) | <0.001 |  |  |  |  |  |  |
| Restriction AM | 13 | 0.71 | -2.77 (-5.77 to 0.23) | 0.067 | 72.3 | 0.010 | <0.001 | 9.68 | -2.99 to -1.34 | y |
|  |  | 0.80 | -2.75 (-5.69 to 0.20) | 0.065 |  |  |  |  |  |  |
|  |  | 0.30 | -2.66 (-5.79 to 0.47) | 0.089 |  |  |  |  |  |  |
| Restriction PM | 19 | 0.71 | -8.35 (-13.1 to -3.56) | 0.002 | 96.0 | <0.001 | 2.13 | 57.5 | -9.36 to -7.16 | n |
|  |  | 0.80 | -8.28 (-13.1 to -3.51) | 0.002 |  |  |  |  |  |  |
|  |  | 0.30 | -8.52 (-13.3 to -3.71) | 0.002 |  |  |  |  |  |  |
| *Early restriction AM* | 5 | 0.71 | 0.25 (-2.55 to 3.05) | 0.817 | <0.001 | 0.988 | <0.001 | 0.001 | -0.22 to 0.41 | y |
|  |  | 0.80 | 0.24 (-2.23 to 2.71) | 0.804 |  |  |  |  |  |  |
|  |  | 0.30 | 0.26 (-4.01 to 4.54) | 0.872 |  |  |  |  |  |  |
| *Early restriction PM* | 6 | 0.71 | -1.10 (-3.42 to 1.23) | 0.280 | 0.05 | 0.391 | <0.001 | 0.003 | -1.66 to -0.62 | y |
|  |  | 0.80 | -1.13 (-3.97 to 1.72) | 0.356 |  |  |  |  |  |  |
|  |  | 0.30 | -1.15 (-4.67 to 2.38) | 0.441 |  |  |  |  |  |  |
| *Late restriction AM* | 8 | 0.71 | -3.46 (-7.22 to 0.31) | 0.067 | 78.1 | 0.002 | <0.001 | 11.2 | -3.80 to -1.79 | y |
|  |  | 0.80 | -3.47 (-7.19 to 0.25) | 0.063 |  |  |  |  |  |  |
|  |  | 0.30 | -3.26 (-7.11 to 0.60) | 0.086 |  |  |  |  |  |  |
| *Late restriction PM* | 13 | 0.71 | -10.1 (-14.9 to -5.13) | 0.001 | 96.3 | <0.001 | <0.001 | 52.6 | -10.9 to -8.89 | n |
|  |  | 0.80 | -10.1 (-14.9 to -5.13) | 0.001 |  |  |  |  |  |  |
|  |  | 0.30 | -10.1 (-15.1 to -5.13) | 0.001 |  |  |  |  |  |  |

**Abbreviations/definitions**: AM: ante meridiem; PM: post meridiem; Deprivation: participants did not sleep for an extended period of time (i.e., whole night); restriction: total sleep time ≤6 h in any 24 h period – this category is a combination of early-restriction, late-restriction, fragmented sleep and sleep restriction protocols not specified; Early restriction: participants delayed sleep (i.e., went to sleep at a later time); Late restriction; participants awakened earlier than normal.

**
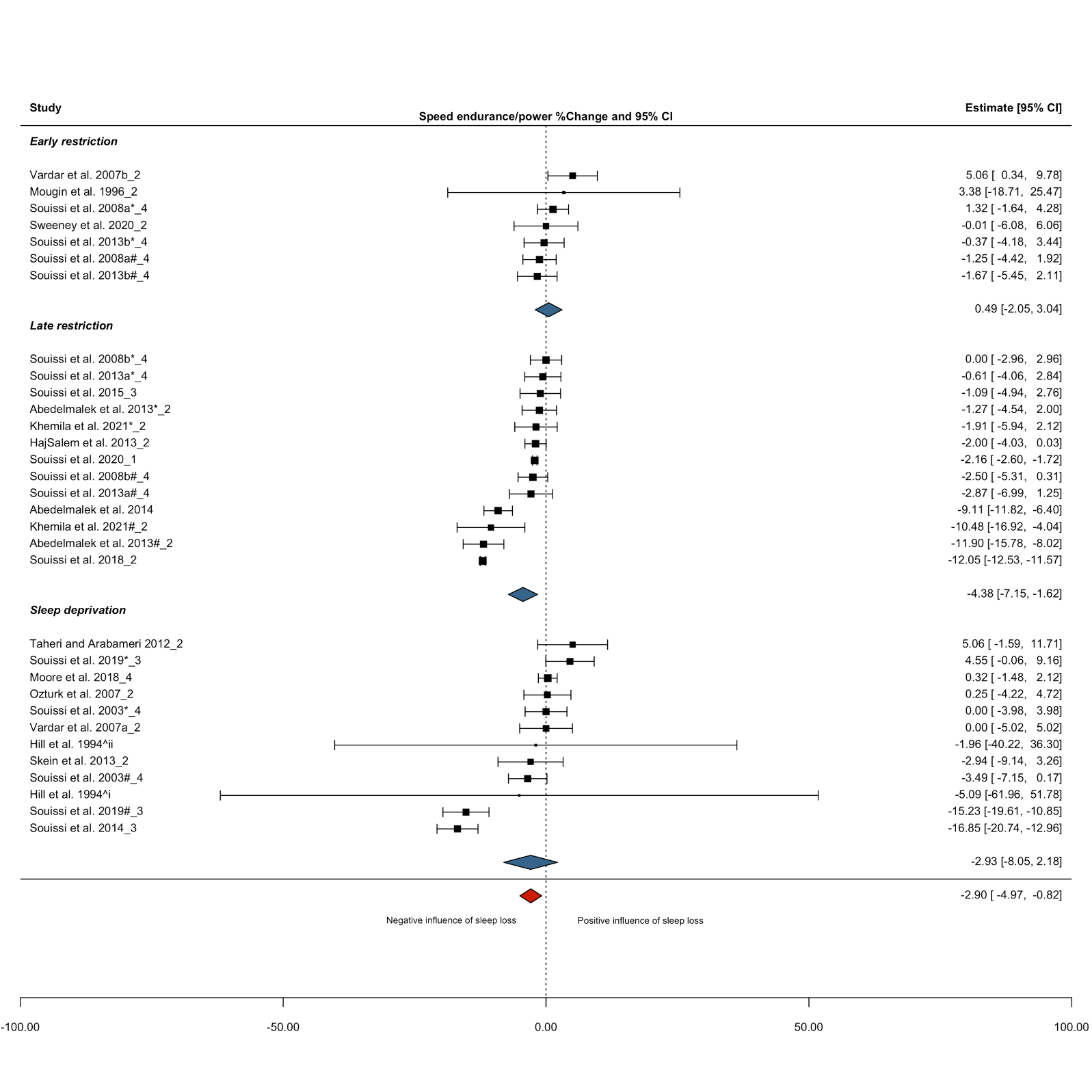
**

**Supplementary Figure S3:** Forest plot displaying the effect of sleep loss vs. normal sleep on the percentage change in speed/power endurance performance. The size of the squares are proportional to the weight of the study. A negative effect estimate indicates a decrease in speed/power endurance performance under the intervention condition (‘sleep loss’). Individual participant pools from a single study are denoted by the addition of a caret followed by a roman numeral (i.e., ^i, ^ii, ^iii etc.) to the citation; different sleep loss protocols (i.e., deprivation and late-restriction or early-restriction) from a single research study are denoted by the addition of a lower-case letter (i.e., a, b) to the citation; * or # indicates task performed in the AM or PM, respectively. * or # followed by a roman numeral value (i.e., *i, *ii, *iii) indicates that the same task was performed at multiple times during AM or PM; underscore numerical value (i.e., _1, _2, _3 etc.) identifies the different tasks performed at a given time. Deprivation: participants did not sleep for an extended period of time (i.e., whole night); restriction: total sleep time ≤6 h in any 24 h period – this category is a combination of early-restriction, late-restriction, fragmented sleep and sleep restriction protocols not specified; Early restriction: participants delayed sleep (i.e., went to sleep at a later time); Late restriction; participants awakened earlier than normal.

**Supplementary Table S9**: Sensitivity analysis of alternative levels of correlation coefficient (R) on speed/power endurance performance meta-analysis results

|  |  |  | **Exercise performance % change** |  | **Heterogeneity** |  |  |  | **Sensitivity analysis – one out** | |
| --- | --- | --- | --- | --- | --- | --- | --- | --- | --- | --- |
| **Exercise category** | **Outcomes, n** | **R** | **Mean (95% CI)** | ***p*** | **I^2^-value** | ***p*** | **σ^2^effect** | **σ^2^study** | **Range (EP%_Δ_)** | **CI crossed (y or n)** |
| **Speed/power endurance** |  |  |  |  |  |  |  |  |  |  |
| Overall | 32 | 0.71 | -2.90 (-4.97 to -0.82) | 0.008 | 96.3 | <0.001 | 26.4 | <0.001 | -3.72 to -2.43 | n |
|  |  | 0.80 | -2.86 (-4.94 to -0.78) | 0.009 |  |  |  |  |  |  |
|  |  | 0.30 | -3.07 (-5.17 to -0.98) | 0.005 |  |  |  |  |  |  |
| **Sleep condition** |  |  |  |  |  |  |  |  |  |  |
| Deprivation | 12 | 0.71 | -2.93 (-8.05 to 2.18) | 0.233 | 91.1 | <0.001 | 49.6 | 0.012 | -3.98 to -1.39 | y |
|  |  | 0.80 | -2.91 (-8.02 to 2.19) | 0.235 |  |  |  |  |  |  |
|  |  | 0.30 | -3.03 (-8.20 to 2.13) | 0.224 |  |  |  |  |  |  |
| Restriction | 20 | 0.71 | -3.23 (-5.94 to -0.53) | 0.022 | 96.4 | <0.001 | 9.33 | 9.86 | -3.82 to -2.41 | y |
|  |  | 0.80 | -3.16 (-5.85 to -0.47) | 0.024 |  |  |  |  |  |  |
|  |  | 0.30 | -3.57 (-6.41 to -0.73) | 0.017 |  |  |  |  |  |  |
| *Early restriction* | 7 | 0.71 | 0.49 (-2.05 to 3.04) | 0.652 | 24.4 | 0.366 | 0.115 | 1.35 | -0.29 to 1.05 | y |
|  |  | 0.80 | 0.67 (-2.18 to 3.52) | 0.584 |  |  |  |  |  |  |
|  |  | 0.30 | 0.27 (-2.68 to 3.21) | 0.833 |  |  |  |  |  |  |
| *Late restriction* | 13 | 0.71 | -4.38 (-7.15 to -1.62) | 0.005 | 97.3 | <0.001 | 18.2 | <0.001 | -5.00 to -3.58 | n |
|  |  | 0.80 | -4.34 (-7.09 to -1.60) | 0.005 |  |  |  |  |  |  |
|  |  | 0.30 | -4.54 (-7.44 to -1.64) | 0.005 |  |  |  |  |  |  |
| **Am vs. PM** |  |  |  |  |  |  |  |  |  |  |
| Overall AM | 14 | 0.71 | 0.11 (-0.94 to 1.16) | 0.823 | <0.001 | 0.721 | <0.001 | <0.001 | -0.09 to 0.24 | y |
|  |  | 0.80 | 0.12 (-0.80 to 1.03) | 0.788 |  |  |  |  |  |  |
|  |  | 0.30 | 0.10 (-1.52 to 1.72) | 0.894 |  |  |  |  |  |  |
| Overall PM | 15 | 0.71 | -6.78 (-10.8 to -2.80) | 0.003 | 98.5 | <0.001 | <0.001 | 38.2 | -7.39 to -5.87 | n |
|  |  | 0.80 | -6.79 (-10.8 to -2.79) | 0.003 |  |  |  |  |  |  |
|  |  | 0.30 | -6.75 (-10.7 to -2.83) | 0.002 |  |  |  |  |  |  |
| **Sleep condition** |  |  |  |  |  |  |  |  |  |  |
| Deprivation AM | 5^a^ | 0.71 | 0.90 (-0.89 to 2.69) | 0.323 | 14.0 | 0.203 |  |  | 0.33 to 1.64 | y |
|  |  | 0.80 | 1.19 (-1.01 to 3.38) | 0.290 |  |  |  |  |  |  |
|  |  | 0.30 | 0.72 (-1.53 to 2.97) | 0.530 |  |  |  |  |  |  |
| Deprivation PM | 5^a^ | 0.71 | -7.11 (-14.4 to 0.20) | 0.057 | 93.9 | <0.001 |  |  | -8.94 to -4.65 | y |
|  |  | 0.80 | -7.11 (-14.4 to 0.20) | 0.057 |  |  |  |  |  |  |
|  |  | 0.30 | -7.17 (-14.5 to 0.14) | 0.055 |  |  |  |  |  |  |

**Supplementary Table S9**: continued

|  |  |  | **Exercise performance % change** |  | **Heterogeneity** |  |  |  | **Sensitivity analysis – one out** | |
| --- | --- | --- | --- | --- | --- | --- | --- | --- | --- | --- |
|  | **Outcomes, n** | **R** | **Mean (95% CI)** | ***p*** | **I^2^-value** | ***p*** | **σ^2^effect** | **σ^2^study** | **Range (EP%_Δ_)** | **CI crossed (y or n)** |
| Restriction AM | 9 | 0.71 | -0.36 (-1.84 to 1.12) | 0.588 | <0.001 | 0.964 | <0.001 | <0.001 | -0.73 to -0.20 | y |
|  |  | 0.80 | -0.37 (-1.65 to 0.92) | 0.530 |  |  |  |  |  |  |
|  |  | 0.30 | -0.36 (-2.65 to 1.93) | 0.726 |  |  |  |  |  |  |
| Restriction PM | 10 | 0.71 | -5.58 (-10.4 to -0.76) | 0.028 | 98.8 | <0.001 | <0.001 | 33.4 | -6.91 to -4.55 | y |
|  |  | 0.80 | -5.54 (-10.4 to -0.68) | 0.030 |  |  |  |  |  |  |
|  |  | 0.30 | -5.74 (-10.4 to -1.07) | 0.021 |  |  |  |  |  |  |
| *Early restriction AM* | 4^a^ | 0.71 | 0.62 (-1.55 to 2.79) | 0.575 | 0.00 | 0.902 |  |  | -0.19 to 1.10 | y |
|  |  | 0.80 | 0.61 (-1.28 to 2.50) | 0.525 |  |  |  |  |  |  |
|  |  | 0.30 | 0.63 (-2.73 to 3.99) | 0.713 |  |  |  |  |  |  |
| *Early restriction PM* | 3^a^ | 0.71 | 0.43 (-3.51 to 4.37) | 0.832 | 68.3 | 0.056 |  |  | -1.42 to 1.65 | y |
|  |  | 0.80 | 0.51 (-3.53 to 4.56) | 0.804 |  |  |  |  |  |  |
|  |  | 0.30 | -0.09 (-3.41 to 3.23) | 0.957 |  |  |  |  |  |  |
| *Late restriction AM* | 5^a^ | 0.71 | -0.86 (-2.40 to 0.68) | 0.275 | 0.00 | 0.954 |  |  | -1.18 to -0.68 | y |
|  |  | 0.80 | -0.86 (-2.20 to 0.48) | 0.209 |  |  |  |  |  |  |
|  |  | 0.30 | -0.86 (-4.23 to 2.52) | 0.519 |  |  |  |  |  |  |
| *Late restriction PM* | 7^a^ | 0.71 | -7.17 (-10.7 to -3.66) | <0.001 | 98.5 | <0.001 |  |  | -8.15 to -6.17 | n |
|  |  | 0.80 | -7.14 (-10.6 to -3.65) | <0.001 |  |  |  |  |  |  |
|  |  | 0.30 | -7.23 (-10.9 to -3.62) | <0.001 |  |  |  |  |  |  |

**Abbreviations/definitions**: AM: ante meridiem; PM: post meridiem; Deprivation: participants did not sleep for an extended period of time (i.e., whole night); restriction: total sleep time ≤6 h in any 24 h period – this category is a combination of early-restriction, late-restriction, fragmented sleep and sleep restriction protocols not specified; Early restriction: participants delayed sleep (i.e., went to sleep at a later time); Late restriction; participants awakened earlier than normal. ^a^ All outcomes were from independent studies and meta-analysis was run without dependency levels (i.e., simple meta-analysis).

**
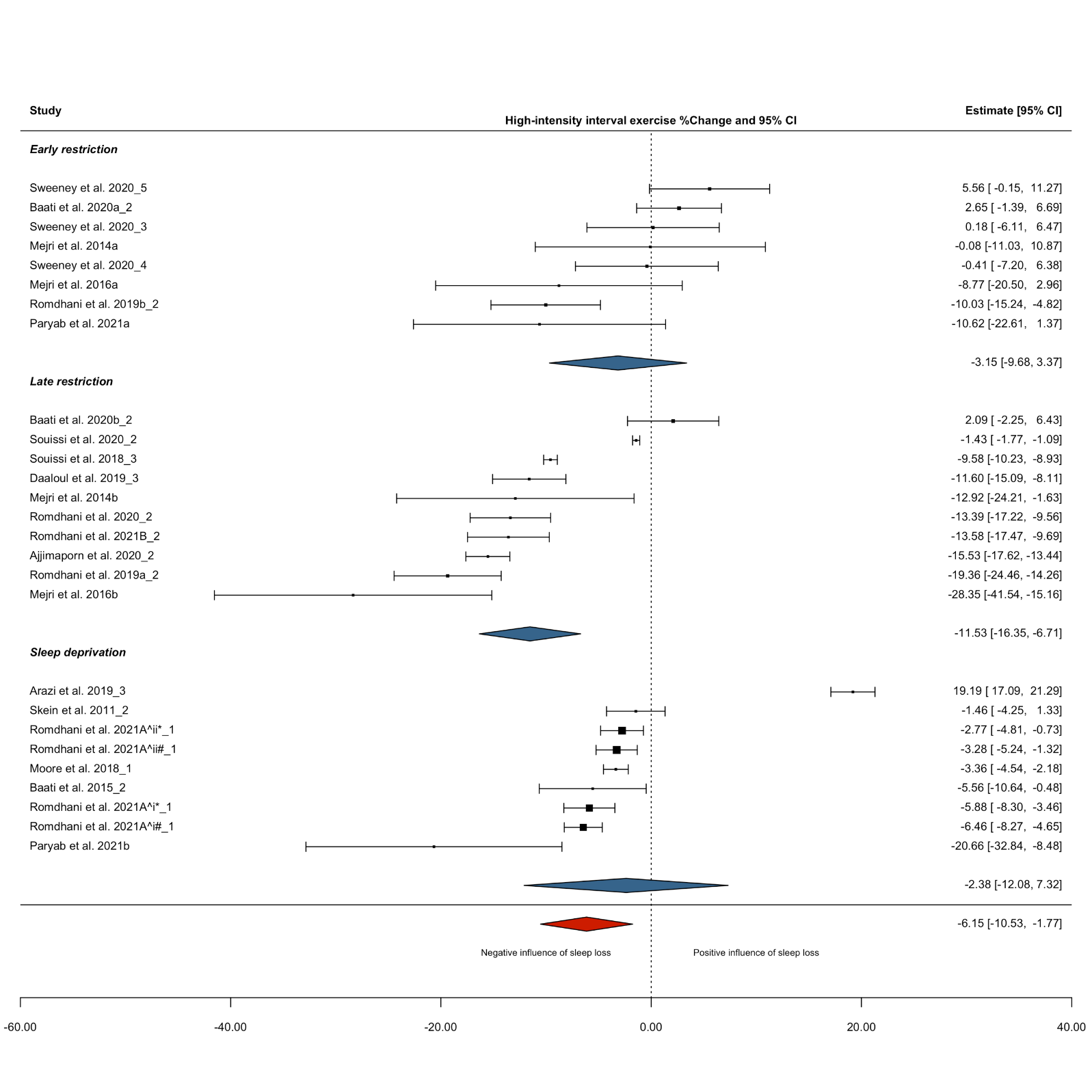
Supplementary Figure S4:** Forest plot displaying the effect of sleep loss vs. normal sleep on the percentage change in high-intensity interval exercise (HIIE) performance. The size of the squares are proportional to the weight of the study. A negative effect estimate indicates a decrease in HIIE performance under the intervention condition (‘sleep loss’). Individual participant pools from a single study are denoted by the addition of a caret followed by a roman numeral (i.e., ^i, ^ii, ^iii etc.) to the citation; different sleep loss protocols (i.e., deprivation and late-restriction or early-restriction) from a single research study are denoted by the addition of a lower-case letter (i.e., a, b) to the citation; * or # indicates task performed in the AM or PM, respectively. * or # followed by a roman numeral value (i.e., *i, *ii, *iii) indicates that the same task was performed at multiple times during AM or PM; underscore numerical value (i.e., _1, _2, _3 etc.) identifies the different tasks performed at a given time. Deprivation: participants did not sleep for an extended period of time (i.e., whole night); restriction: total sleep time ≤6 h in any 24 h period – this category is a combination of early-restriction, late-restriction, fragmented sleep and sleep restriction protocols not specified; Early restriction: participants delayed sleep (i.e., went to sleep at a later time); Late restriction; participants awakened earlier than normal.

**Supplementary Table S10**: Sensitivity analysis of alternative levels of correlation coefficient (R) on high-intensity interval exercise performance meta-analysis results

|  |  |  | **Exercise performance % change** |  | **Heterogeneity** |  |  |  | **Sensitivity analysis – one out** | |
| --- | --- | --- | --- | --- | --- | --- | --- | --- | --- | --- |
| **Exercise category** | **Outcomes, n** | **R** | **Mean (95% CI)** | ***p*** | **I^2^-value** | ***p*** | **σ^2^ effect** | **σ^2^study** | **Range (EP%_Δ_)** | **CI crossed (y or n)** |
| **HIIE** |  |  |  |  |  |  |  |  |  |  |
| Overall | 27 | 0.71 | -6.15 (-10.5 to -1.77) | 0.008 | 98.9 | <0.001 | <0.001 | 77.4 | -7.54 to -5.57 | n |
|  |  | 0.80 | -6.22 (-10.6 to -1.88) | 0.007 |  |  |  |  |  |  |
|  |  | 0.30 | -5.89 (-10.3 to -1.48) | 0.011 |  |  |  |  |  |  |
| **Sleep condition** |  |  |  |  |  |  |  |  |  |  |
| Deprivation | 9 | 0.71 | -2.38 (-12.1 to 7.32) | 0.587 | 99.0 | <0.001 | <0.001 | 118 | -4.21 to -0.04 | y |
|  |  | 0.80 | -2.52 (-12.3 to 7.25) | 0.568 |  |  |  |  |  |  |
|  |  | 0.30 | -1.76 (-11.0 to 7.46) | 0.671 |  |  |  |  |  |  |
| Restriction | 18 | 0.71 | -8.77 (-13.3 to -4.27) | 0.001 | 98.3 | <0.001 | 12.1 | 38.7 | -9.50 to -8.11 | n |
|  |  | 0.80 | -8.77 (-13.3 to -4.23) | 0.001 |  |  |  |  |  |  |
|  |  | 0.30 | -8.71 (-13.2 to -4.23) | 0.001 |  |  |  |  |  |  |
| *Early restriction* | 8 | 0.71 | -3.15 (-9.68 to 3.37) | 0.291 | 73.2 | 0.001 | 2.52 | 28.4 | -4.78 to -0.24 | y |
|  |  | 0.80 | -3.28 (-9.71 to 3.14) | 0.266 |  |  |  |  |  |  |
|  |  | 0.30 | -2.42 (-9.34 to 4.50) | 0.436 |  |  |  |  |  |  |
| *Late restriction* | 10^a^ | 0.71 | -11.5 (-16.3 to -6.71) | <0.001 | 99.2 | <0.001 |  |  | -12.9 to -10.4 | n |
|  |  | 0.80 | -11.7 (-16.6 to -6.79) | <0.001 |  |  |  |  |  |  |
|  |  | 0.30 | -10.9 (-15.5 to -6.27) | <0.001 |  |  |  |  |  |  |
| **AM vs. PM** |  |  |  |  |  |  |  |  |  |  |
| Overall AM | 11 | 0.71 | -1.51 (-10.4 to 7.42) | 0.714 | 97.7 | <0.001 | 6.85 | 101 | -4.32 to -0.60 | y |
|  |  | 0.80 | 1.65 (-10.5 to 7.25) | 0.689 |  |  |  |  |  |  |
|  |  | 0.30 | -1.02 (-9.99 to 7.96) | 0.806 |  |  |  |  |  |  |
| Overall PM | 16 | 0.71 | -8.34 (-12.2 to -4.47) | 0.001 | 98.5 | <0.001 | 14.1 | 27.1 | -8.94 to -7.72 | n |
|  |  | 0.80 | -8.41 (-12.4 to -4.47) | 0.001 |  |  |  |  |  |  |
|  |  | 0.30 | -8.02 (-11.7 to -4.38) | 0.001 |  |  |  |  |  |  |
| **Sleep condition** |  |  |  |  |  |  |  |  |  |  |
| Deprivation AM | 5^a^ | 0.71 | -2.06 (-14.1 to 10.0) | 0.737 | 99.3 | <0.001 |  |  | -4.34 to 1.79 | y |
|  |  | 0.80 | 2.20 (-14.4 to 9.95) | 0.722 |  |  |  |  |  |  |
|  |  | 0.30 | 1.32 (-12.9 to 10.3) | 0.824 |  |  |  |  |  |  |

**Supplementary Table S10**: continued

|  |  |  | **Exercise performance % change** |  | **Heterogeneity** | |  |  | **Sensitivity analysis – one out** | |
| --- | --- | --- | --- | --- | --- | --- | --- | --- | --- | --- |
|  | **Outcomes, n** | **R** | **Mean (95% CI)** | ***p*** | **I^2^-value** | ***p*** | **σ^2^ effect** | **σ^2^study** | **Range (EP%_Δ_)** | **CI crossed (y or n)** |
| Deprivation PM | 4^a^ | 0.71 | -4.13 (-6.50 to -1.76) | <0.001 | 81.2 | 0.013 |  |  | -5.01 to -2.94 | y |
|  |  | 0.80 | -4.20 (-6.46 to -1.94) | <0.001 |  |  |  |  |  |  |
|  |  | 0.30 | -4.33 (-6.62 to -2.04) | <0.001 |  |  |  |  |  |  |
| Restriction AM | 6 | 0.71 | -3.39 (-13.3 to 6.55) | 0.421 | 66.5 | 0.074 | 5.94 | 26.9 | -5.78 to -1.10 | y |
|  |  | 0.80 | -3.60 (-13.4 to 6.23) | 0.390 |  |  |  |  |  |  |
|  |  | 0.30 | -1.99 (-12.1 to 8.12) | 0.634 |  |  |  |  |  |  |
| Restriction PM | 12 | 0.71 | -10.2 (-15.4 to -4.97) | 0.001 | 99.0 | <0.001 | 16.8 | 33.3 | -11.3 to -9.44 | n |
|  |  | 0.80 | -10.3 (-15.6 to -4.93) | 0.001 |  |  |  |  |  |  |
|  |  | 0.30 | -9.95 (-14.9 to -4.94) | 0.001 |  |  |  |  |  |  |
| *Early restriction AM* | 5 | 0.71 | -1.10 (-10.7 to 8.53) | 0.767 | 57.2 | 0.176 | 2.54 | 17.4 | -2.87 to 1.92 | y |
|  |  | 0.80 | -1.47 (-11.3 to 8.40) | 0.700 |  |  |  |  |  |  |
|  |  | 0.30 | 0.97 (-6.23 to 8.18) | 0.762 |  |  |  |  |  |  |
| *Early restriction PM* | 3^a^ | 0.71 | -4.79 (-13.6 to 3.98) | 0.284 | 83.6 | <0.001 |  |  | -9.82 to -1.68 | y |
|  |  | 0.80 | -4.89 (-13.5 to 3.73) | 0.266 |  |  |  |  |  |  |
|  |  | 0.30 | -4.32 (-13.7 to 5.09) | 0.368 |  |  |  |  |  |  |
| *Late restriction AM* | 1 |  |  |  |  |  |  |  |  |  |
| *Late restriction PM* | 9^a^ | 0.71 | -11.5 (-16.7 to -6.24) | <0.001 | 99.4 | <0.001 |  |  | -12.9 to -10.2 | n |
|  |  | 0.80 | -11.6 (-16.9 to -6.28) | <0.001 |  |  |  |  |  |  |
|  |  | 0.30 | -10.8 (-15.7 to -5.97) | <0.001 |  |  |  |  |  |  |

**Abbreviations/definitions**: HIIE: high-intensity interval exercise; AM: ante meridiem; PM: post meridiem; Deprivation: participants did not sleep for an extended period of time (i.e., whole night); restriction: total sleep time ≤6 h in any 24 h period – this category is a combination of early-restriction, late-restriction, fragmented sleep and sleep restriction protocols not specified; Early restriction: participants delayed sleep (i.e., went to sleep at a later time); Late restriction; participants awakened earlier than normal. ^a^ All outcomes were from independent studies and meta-analysis was run without dependency levels (i.e., simple meta-analysis).

**
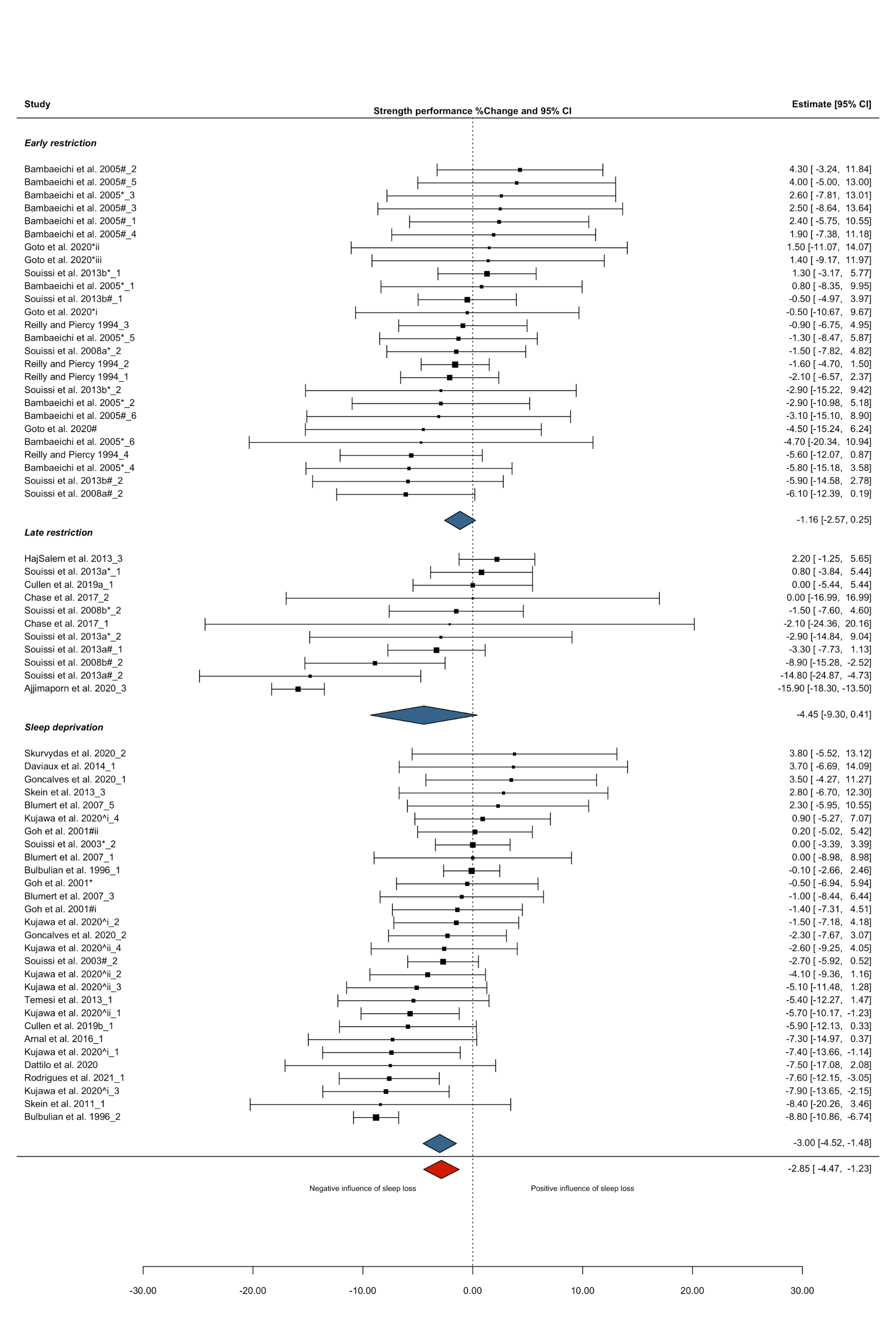
**

**Supplementary Figure S5:** Forest plot displaying the effect of sleep loss vs. normal sleep on the percentage change in strength performance. The size of the squares are proportional to the weight of the study. A negative effect estimate indicates a decrease in strength performance under the intervention condition (‘sleep loss’). Individual participant pools from a single study are denoted by the addition of a caret followed by a roman numeral (i.e., ^i, ^ii, ^iii etc.) to the citation; different sleep loss protocols (i.e., deprivation and late-restriction or early-restriction) from a single research study are denoted by the addition of a lower-case letter (i.e., a, b) to the citation; * or # indicates task performed in the AM or PM, respectively. * or # followed by a roman numeral value (i.e., *i, *ii, *iii) indicates that the same task was performed at multiple times during AM or PM; underscore numerical value (i.e., _1, _2, _3 etc.) identifies the different tasks performed at a given time. Deprivation: participants did not sleep for an extended period of time (i.e., whole night); restriction: total sleep time ≤6 h in any 24 h period – this category is a combination of early-restriction, late-restriction, fragmented sleep and sleep restriction protocols not specified; Early restriction: participants delayed sleep (i.e., went to sleep at a later time); Late restriction; participants awakened earlier than normal

**Supplementary Table S11**: Sensitivity analysis of alternative levels of correlation coefficient (R) on strength performance meta-analysis results

|  |  |  | **Exercise performance% change** |  | **Heterogeneity** |  |  |  | **Sensitivity analysis – one out** | |
| --- | --- | --- | --- | --- | --- | --- | --- | --- | --- | --- |
| **Exercise category** | **Outcomes, n** | **R** | **Mean (95% CI)** | ***p*** | **I^2^-value** | ***p*** | **σ^2^effect** | **σ^2^study** | **Range (EP%_Δ_)** | **CI crossed (y or n)** |
| **Strength** |  |  |  |  |  |  |  |  |  |  |
| Overall | 66 | 0.71 | -2.85 (-4.47 to -1.23) | <0.001 | 62.2 | <0.001 | 6.45 | 6.83 | -3.20 to -2.27 | n |
|  |  | 0.80 | -2.84 (-4.41 to -1.27) | <0.001 |  |  |  |  |  |  |
|  |  | 0.30 | -3.10 (-4.95 to -1.25) | 0.001 |  |  |  |  |  |  |
| **Sleep condition** |  |  |  |  |  |  |  |  |  |  |
| Deprivation | 29 | 0.71 | -3.00 (-4.52 to -1.48) | <0.001 | 49.2 | <0.001 | 6.58 | 0.003 | -3.53 to -2.53 | n |
|  |  | 0.80 | -3.05 (-4.55 to -1.56) | <0.001 |  |  |  |  |  |  |
|  |  | 0.30 | -3.29 (-5.15 to -1.44) | 0.001 |  |  |  |  |  |  |
| Restriction | 37 | 0.71 | -2.77 (-6.75 to 1.21) | 0.167 | 74.9 | <0.001 | <0.001 | 29.3 | -3.44 to -1.08 | y |
|  |  | 0.80 | -2.74 (-6.62 to 1.14) | 0.161 |  |  |  |  |  |  |
|  |  | 0.30 | -2.96 (-7.17 to 1.25) | 0.163 |  |  |  |  |  |  |
| *Early restriction* | 26 | 0.71 | -1.16 (-2.57 to 0.25) | 0.102 | 0.02 | 0.952 | 0.001 | 0.001 | -1.41 to -0.93 | y |
|  |  | 0.80 | -1.14 (-2.59 to 0.31) | 0.119 |  |  |  |  |  |  |
|  |  | 0.30 | -1.20 (-3.36 to 0.96) | 0.264 |  |  |  |  |  |  |
| *Late restriction* | 11 | 0.71 | -4.45 (-9.30 to 0.41) | 0.068 | 83.7 | <0.001 | 36.2 | <0.001 | -5.37 to -2.36 | y |
|  |  | 0.80 | -4.45 (-9.20 to 0.30) | 0.064 |  |  |  |  |  |  |
|  |  | 0.30 | -4.38 (-11.2 to 2.46) | 0.184 |  |  |  |  |  |  |
| **AM vs. PM** |  |  |  |  |  |  |  |  |  |  |
| Overall AM | 39 | 0.71 | -1.78 (-3.23 to -0.33) | 0.017 | 17.6 | 0.570 | <0.001 | 2.53 | -1.96 to -1.45 | n |
|  |  | 0.80 | -1.74 (-3.22 to -0.27) | 0.022 |  |  |  |  |  |  |
|  |  | 0.30 | -2.01 (-3.73 to -0.30) | 0.022 |  |  |  |  |  |  |
| Overall PM | 26 | 0.71 | -4.58 (-7.59 to -1.58) | 0.004 | 79.5 | <0.001 | 7.90 | 14.6 | -5.20 to -3.15 | n |
|  |  | 0.80 | -4.60 (-7.54 to -1.66) | 0.004 |  |  |  |  |  |  |
|  |  | 0.30 | -4.55 (-7.79 to -1.31) | 0.008 |  |  |  |  |  |  |
| **Sleep condition** |  |  |  |  |  |  |  |  |  |  |
| Deprivation AM | 21 | 0.71 | -2.43 (-4.47 to -0.38) | 0.022 | 31.8 | 0.115 | 0.87 | 3.80 | -2.81 to -1.94 | y |
|  |  | 0.80 | -2.42 (-4.51 to -0.34) | 0.025 |  |  |  |  |  |  |
|  |  | 0.30 | -2.84 (-5.02 to -0.65) | 0.014 |  |  |  |  |  |  |
| Deprivation PM | 8 | 0.71 | -3.79 (-7.27 to -0.32) | 0.037 | 71.5 | <0.001 | 10.3 | <0.001 | -5.06 to -2.01 | y |
|  |  | 0.80 | -3.83 (-7.20 to -0.45) | 0.032 |  |  |  |  |  |  |
|  |  | 0.30 | -3.82 (-7.81 to 0.18) | 0.058 |  |  |  |  |  |  |

**Supplementary Table S11**: continued

|  |  |  | **Exercise performance% change** |  | **Heterogeneity** |  |  |  | **Sensitivity analysis – one out** | |
| --- | --- | --- | --- | --- | --- | --- | --- | --- | --- | --- |
|  | **Outcomes, n** | **R** | **Mean (95% CI)** | ***p*** | **I^2^-value** | ***p*** | **σ^2^effect** | **σ^2^study** | **Range (EP%_Δ_)** | **CI crossed (y or n)** |
| Restriction AM | 18 | 0.71 | -0.43 (-2.41 to 1.54) | 0.650 | <0.001 | 0.999 | <0.001 | <0.001 | -0.78 to -0.22 | y |
|  |  | 0.80 | -0.40 (-2.14 to 1.34) | 0.636 |  |  |  |  |  |  |
|  |  | 0.30 | -0.47 (-3.50 to 2.56) | 0.747 |  |  |  |  |  |  |
| Restriction PM | 18 | 0.71 | -5.20 (-11.0 to 0.59) | 0.075 | 82.3 | <0.001 | <0.001 | 39.5 | -5.54 to -2.61 | y |
|  |  | 0.80 | -5.18 (-10.9 to 0.53) | 0.073 |  |  |  |  |  |  |
|  |  | 0.30 | -5.29 (-11.4 to 0.82) | 0.085 |  |  |  |  |  |  |
| *Early restriction AM* | 12 | 0.71 | -0.55 (-3.21 to 2.11) | 0.659 | <0.001 | 0.985 | <0.001 | <0.001 | -1.26 to -0.19 | y |
|  |  | 0.80 | -0.49 (-2.84 to 1.86) | 0.656 |  |  |  |  |  |  |
|  |  | 0.30 | -0.61 (-4.69 to 3.47) | 0.747 |  |  |  |  |  |  |
| *Early restriction PM* | 14 | 0.71 | -1.51 (-4.41 to 1.39) | 0.281 | 27.1 | 0.631 | <0.001 | 3.86 | -2.13 to -0.86 | y |
|  |  | 0.80 | -1.60 (-4.60 to 1.39) | 0.268 |  |  |  |  |  |  |
|  |  | 0.30 | -1.48 (-4.23 to 1.28) | 0.267 |  |  |  |  |  |  |
| *Late restriction AM* | 6 | 0.71 | -0.26 (-4.06 to 3.54) | 0.867 | <0.001 | 0.989 | <0.001 | <0.001 | -0.90 to 0.11 | y |
|  |  | 0.80 | -0.26 (-3.60 to 3.08) | 0.848 |  |  |  |  |  |  |
|  |  | 0.30 | -0.26 (-6.08 to 5.57) | 0.915 |  |  |  |  |  |  |
| *Late restriction PM* | 4 | 0.71 | -10.5 (-20.6 to -0.39) | 0.046 | 84.0 | <0.001 | 31.3 | <0.001 | -13.6 to -7.77 | y |
|  |  | 0.80 | -10.5 (-20.4 to -0.59) | 0.043 |  |  |  |  |  |  |
|  |  | 0.30 | -10.6 (-21.5 to 0.41) | 0.055 |  |  |  |  |  |  |
| **Upper- vs. lower body strength** |  |  |  |  |  |  |  |  |  |  |
| Overall upper-body | 18 | 0.71 | -1.63 (-3.30 to 0.04) | 0.056 | 32.7 | 0.069 | 3.42 | <0.001 | -1.99 to -0.96 | y |
|  |  | 0.80 | -1.72 (-3.45 to -0.00) | 0.049 |  |  |  |  |  |  |
|  |  | 0.30 | -1.44 (-3.42 to 0.55) | 0.145 |  |  |  |  |  |  |
| Overall lower-body | 46 | 0.71 | -3.42 (-5.54 to -1.31) | 0.002 | 65.6 | <0.001 | 7.68 | 8.44 | -3.96 to -2.64 | n |
|  |  | 0.80 | -3.41 (-5.48 to -1.34) | 0.002 |  |  |  |  |  |  |
|  |  | 0.30 | -3.82 (-6.22 to -1.42) | 0.003 |  |  |  |  |  |  |
| **Sleep condition** |  |  |  |  |  |  |  |  |  |  |
| Upper-body Deprivation | 6 | 0.71 | -3.18 (-9.13 to 2.77) | 0.228 | 58.9 | 0.104 | <0.001 | 13.2 | -4.37 to -1.41 | y |
|  |  | 0.80 | -3.06 (-9.16 to 3.03) | 0.253 |  |  |  |  |  |  |
|  |  | 0.30 | -2.91 (-7.58 to 1.76) | 0.170 |  |  |  |  |  |  |

**Supplementary File S11**: continued

|  |  |  | **Exercise performance% change** |  | **Heterogeneity** |  |  |  | **Sensitivity analysis – one out** | |
| --- | --- | --- | --- | --- | --- | --- | --- | --- | --- | --- |
|  | **Outcomes, n** | **R** | **Mean (95% CI)** | ***p*** | **I^2^-value** | ***p*** | **σ^2^effect** | **σ^2^study** | **Range (EP%_Δ_)** | **CI crossed (y or n)** |
| Upper-body Restriction | 12 | 0.71 | -0.73 (-2.67 to 1.22) | 0.428 | 11.5 | 0.186 | <0.001 | 0.84 | -1.49 to -0.54 | y |
|  |  | 0.80 | -1.11 (-2.91 to 0.68) | 0.199 |  |  |  |  |  |  |
|  |  | 0.30 | -0.90 (-3.32 to 1.52) | 0.432 |  |  |  |  |  |  |
| *Early restriction* | 6 | 0.71 | -1.21 (-3.71 to 1.29) | 0.268 | <0.001 | 0.752 | <0.001 | <0.001 | -1.76 to -0.98 | y |
|  |  | 0.80 | -1.17 (-3.38 to 1.04) | 0.232 |  |  |  |  |  |  |
|  |  | 0.30 | -1.25 (-5.08 to 2.58) | 0.440 |  |  |  |  |  |  |
| *Late restriction* | 6 | 0.71 | -1.13 (-6.07 to 3.81) | 0.583 | 61.6 | 0.035 | 7.75 | 2.31 | -2.72 to 0.12 | y |
|  |  | 0.80 | -1.76 (-7.18 to 3.66) | 0.442 |  |  |  |  |  |  |
|  |  | 0.30 | -0.42 (-4.98 to 4.13) | 0.824 |  |  |  |  |  |  |
| Lower-body Deprivation | 21 | 0.71 | -3.25 (-5.09 to -1.41) | 0.002 | 54.7 | <0.001 | 7.34 | <0.001 | -3.65 to -2.43 | n |
|  |  | 0.80 | -3.36(-5.16 to -1.56) | 0.001 |  |  |  |  |  |  |
|  |  | 0.30 | -3.53 (-5.77 to -1.30) | 0.004 |  |  |  |  |  |  |
| Lower-body Restriction | 25 | 0.71 | -4.50 (-10.2 to 1.17) | 0.114 | 72.8 | <0.001 | <0.001 | 37.8 | -4.94 to -1.86 | y |
|  |  | 0.80 | -4.42 (-10.0 to 1.16) | 0.115 |  |  |  |  |  |  |
|  |  | 0.30 | -4.81 (-10.9 to 1.27) | 0.116 |  |  |  |  |  |  |
| *Early restriction* | 20 | 0.71 | -1.36 (-3.81 to 1.09) | 0.259 | 6.97 | 0.892 | <0.001 | 1.41 | -1.48 to -0.62 | y |
|  |  | 0.80 | -1.44 (-3.85 to 0.97) | 0.226 |  |  |  |  |  |  |
|  |  | 0.30 | -1.15 (-4.25 to 1.95) | 0.446 |  |  |  |  |  |  |
| *Late restriction* | 5 | 0.71 | -8.26 (-20.4 to 3.90) | 0.132 | 81.7 | <0.001 | 15.1 | 34.2 | -10.5 to -4.45 | y |
|  |  | 0.80 | -8.03 (-20.0 to 3.98) | 0.137 |  |  |  |  |  |  |
|  |  | 0.30 | -9.43 (-22.4 to 3.51) | 0.113 |  |  |  |  |  |  |

**Abbreviations/definitions**: AM: ante meridiem; PM: post meridiem; Deprivation: participants did not sleep for an extended period of time (i.e., whole night); restriction: total sleep time ≤6 h in any 24 h period – this category is a combination of early-restriction, late-restriction, fragmented sleep and sleep restriction protocols not specified; Early restriction: participants delayed sleep (i.e., went to sleep at a later time); Late restriction; participants awakened earlier than normal.

**Supplementary Table S12:** Effect of time of day following sleep loss on body-limb strength

|  |  | **Exercise performance** |  |
| --- | --- | --- | --- |
|  |  | **% change** |  |
| **Exercise category** | Outcomes, n | Mean (95% CI) | *p* |
| **Upper-body Am vs. PM** |  |  |  |
| Overall upper-body (exercise AM) | 9 | -1.85 (-5.97 to 2.26) | 0.329 |
| Overall upper-body (exercise PM) | 8 | -2.10 (-4.16 to -0.05) | 0.046 |
| **Sleep condition** |  |  |  |
| Deprivation (exercise AM) | 4^a^ | -3.54 (-8.22 to 1.16) | 0.139 |
| Deprivation (exercise PM) | 2 | -0.54 (-26.0 to 24.9) | 0.831 |
| Restriction (exercise AM) | 5 | 0.41 (3.33 to 4.14) | 0.777 |
| Restriction (exercise PM) | 6 | -2.47 (-4.94 to 0.01) | 0.051 |
| *Early restriction (exercise AM)* | 2 | 0.78 (-26.4 to 28.0) | 0.778 |
| *Early restriction (exercise PM)* | 4 | -1.73 (-5.20 to 1.74) | 0.211 |
| *Late restriction (exercise AM)* | 3 | 0.17 (-7.28 to 7.61) | 0.933 |
| *Late restriction (exercise PM)* | 2 | -8.09 (-80.4 to 64.2) | 0.390 |
| **Lower-body Am vs. Pm** |  |  |  |
| Overall (exercise AM) | 28 | -2.16 (-3.53 to -0.79) | 0.003 |
| Overall (exercise PM) | 18 | -5.27 (-9.04 to 1.49) | 0.009 |
| **Sleep condition** |  |  |  |
| Deprivation (exercise AM) | 15 | -2.01 (-4.41 to 0.39) | 0.094 |
| Deprivation (exercise PM) | 6 | -4.81 (-9.33 to -0.28) | 0.041 |
| Restriction (exercise AM) | 13 | -1.22 (-4.06 to 1.62) | 0.368 |
| Restriction (exercise PM) | 12 | -5.84 (-13.2 to 1.49) | 0.107 |
| *Early restriction (exercise AM)* | 10 | -1.17 (-4.48 to 2.14) | 0.445 |
| *Early restriction (exercise PM)* | 10 | -1.07 (-3.97 to 1.83) | 0.424 |
| *Late restriction (exercise AM)* | 3 | -1.40 (-13.6 to 10.8) | 0.670 |
| *Late restriction (exercise PM)* | 2 | -13.0 (-19.7 to -6.31) | <0.001 |

**Abbreviations/definitions:** AM: ante meridiem; PM: post meridiem; CI: confidence interval; Deprivation: participants did not sleep for an extended period of time (i.e., whole night); restriction: total sleep time ≤6 h in any 24 h period – this category is a combination of early-restriction, late-restriction, fragmented sleep and sleep restriction protocols not specified; Early restriction: participants delayed sleep (i.e., went to sleep at a later time); Late restriction; participants awakened earlier than normal. ^a^ Outcome was from independent study and meta-analysis was run without dependency levels (i.e., simple meta-analysis).

**
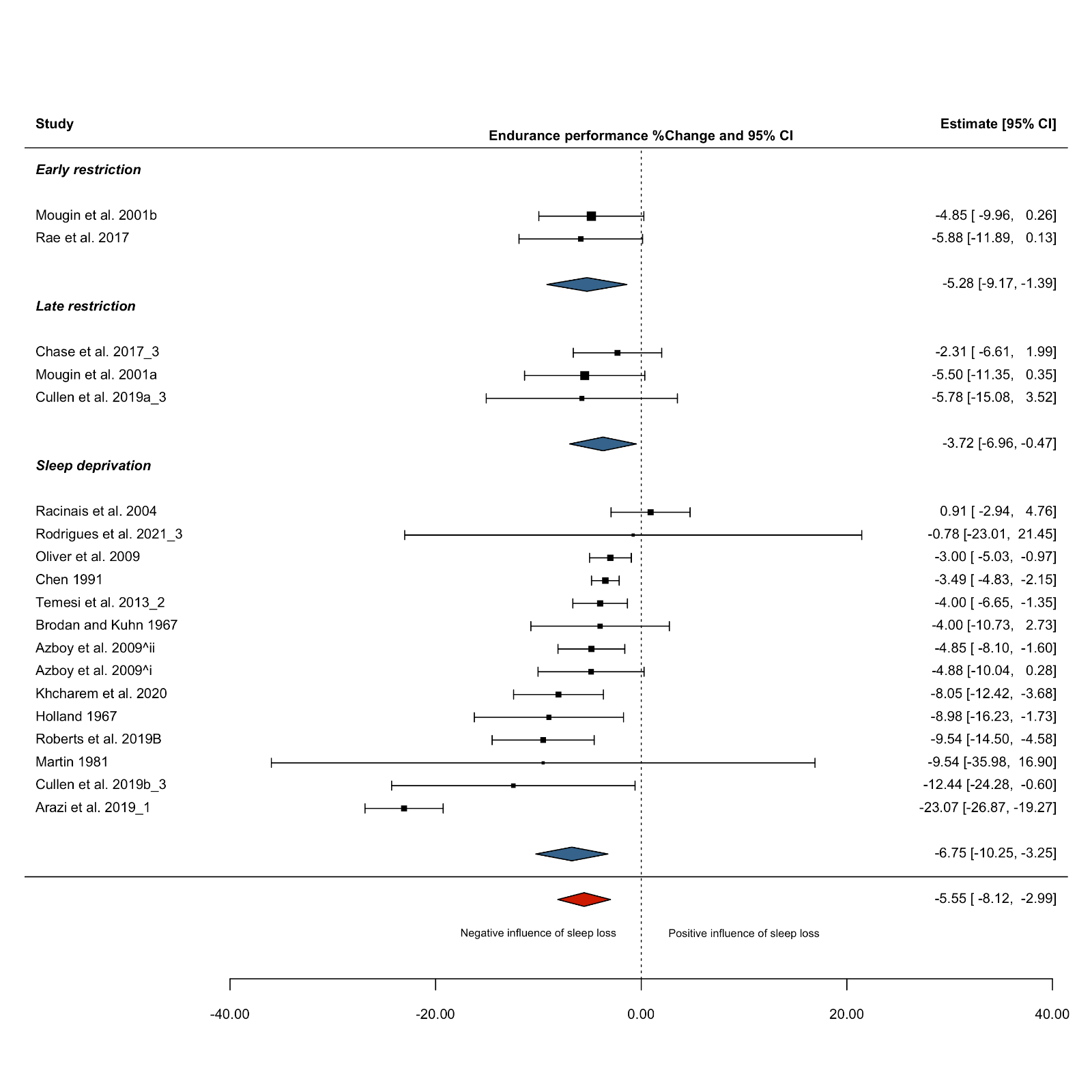
**

**Supplementary Figure S6:** Forest plot displaying the effect of sleep loss vs. normal sleep on the percentage change in endurance performance. The size of the squares are proportional to the weight of the study. A negative effect estimate indicates a decrease in endurance performance under the intervention condition (‘sleep loss’). Individual participant pools from a single study are denoted by the addition of a caret followed by a roman numeral (i.e., ^i, ^ii, ^iii etc.) to the citation; different sleep loss protocols (i.e., deprivation and late-restriction or early-restriction) from a single research study are denoted by the addition of a lower-case letter (i.e., a, b) to the citation; * or # indicates task performed in AM or PM, respectively. * or # followed by roman numeral value (i.e., *i, *ii, *iii) indicates that the same task was performed at multiple times during AM or PM; underscore numerical value (i.e., _1, _2, _3 etc.) identifies the different tasks performed at a given time; studies conducted by the same author in the same year are denoted by the addition of a capital ‘a or ‘b’ (i.e., A, B) to the citation. Deprivation: participants did not sleep for an extended period of time (i.e., whole night); restriction: total sleep time ≤6 h in any 24 h period – this category is a combination of early-restriction, late-restriction, fragmented sleep and sleep restriction protocols not specified; Early restriction: participants delayed sleep (i.e., went to sleep at a later time); Late restriction; participants awakened earlier than normal. Note, trials from Mougin 1991 [49] (fragmented sleep), Omiya et al. 2009 [128] (type of sleep restriction not stated), and Roberts et al. 2019 [51] (type of sleep restriction not stated) are included in overall effect estimate.

**Supplementary Table S13**: Sensitivity analysis of alternative levels of correlation coefficient (R) on endurance performance meta-analysis results

|  |  |  | **Exercise performance % change** |  | **Heterogeneity** |  |  |  | **Sensitivity analysis – one out** | |
| --- | --- | --- | --- | --- | --- | --- | --- | --- | --- | --- |
| **Exercise category** | **Outcomes, n** | **R** | **Mean (95% CI)** | ***p*** | **I^2^-value** | ***p*** | **σ^2^effect** | **σ^2^study** | **Range (EP%_Δ_)** | **CI crossed**  **(y or n)** |
| **Endurance** |  |  |  |  |  |  |  |  |  |  |
| Overall | 22 | 0.71 | -5.55 (-8.12 to -2.99) | <0.001 | 86.5 | <0.001 | <0.001 | 22.6 | -5.94 to -3.72 | n |
|  |  | 0.80 | -5.64 (-8.26 to -3.01) | <0.001 |  |  |  |  |  |  |
|  |  | 0.30 | -5.63 (-8.43 to -2.83) | <0.001 |  |  |  |  |  |  |
| **Sleep condition** |  |  |  |  |  |  |  |  |  |  |
| Deprivation | 14^a^ | 0.71 | -6.75 (-10.3 to -3.25) | <0.001 | 91.2 | <0.001 |  |  | -7.46 to -4.53 | n |
|  |  | 0.80 | -6.89 (-10.5 to -3.32) | <0.001 |  |  |  |  |  |  |
|  |  | 0.30 | -6.93 (-10.8 to -3.05) | <0.001 |  |  |  |  |  |  |
| Restriction | 8 | 0.71 | -3.27 (-5.06 to -1.47) | 0.004 | <0.001 | 0.914 | <0.001 | <0.001 | -3.80 to -3.09 | n |
|  |  | 0.80 | -3.32 (-4.96 to -1.68) | 0.002 |  |  |  |  |  |  |
|  |  | 0.30 | -3.21 (-5.85 to -0.56) | 0.024 |  |  |  |  |  |  |
| *Early restriction* | 2^a^ | 0.71 | -5.28 (-9.17 to -1.39) | 0.008 | 0.00 | 0.798 |  |  | -5.88 to -4.85 | y |
|  |  | 0.80 | -5.30 (-8.71 to -1.90) | 0.002 |  |  |  |  |  |  |
|  |  | 0.30 | -5.28 (-11.3 to 0.74) | 0.086 |  |  |  |  |  |  |
| *Late restriction* | 3^a^ | 0.71 | -3.72 (-6.96 to -0.47) | 0.025 | <0.001 | 0.620 |  |  | -5.58 to -2.92 | y |
|  |  | 0.80 | -3.77 (-6.75 to -0.80) | 0.013 |  |  |  |  |  |  |
|  |  | 0.30 | -3.68 (-8.53 to 1.17) | 0.137 |  |  |  |  |  |  |
| **AM vs. PM** |  |  |  |  |  |  |  |  |  |  |
| Overall AM | 12 | 0.71 | -6.50 (-11.1 to -1.86) | 0.010 | 88.6 | <0.001 | <0.001 | 39.9 | -7.35 to -4.41 | n |
|  |  | 0.80 | -6.48 (-11.1 to -1.88) | 0.010 |  |  |  |  |  |  |
|  |  | 0.30 | -6.56 (-11.4 to -1.74) | 0.012 |  |  |  |  |  |  |
| Overall PM | 9 | 0.71 | -3.56 (-4.67 to -2.45) | <0.001 | <0.001 | 0.976 | <0.001 | <0.001 | -3.71 to -3.49 | n |
|  |  | 0.80 | -3.63 (-4.79 to -2.48) | <0.001 |  |  |  |  |  |  |
|  |  | 0.30 | -3.64 (-5.71 to -1.58) | 0.004 |  |  |  |  |  |  |
| **Sleep condition** |  |  |  |  |  |  |  |  |  |  |
| Deprivation AM | 9^a^ | 0.71 | -7.83 (-12.9 to -2.72) | 0.003 | 88.5 | <0.001 |  |  | -9.17 to -5.27 | n |
|  |  | 0.80 | -7.79 (-12.8 to -2.73) | 0.003 |  |  |  |  |  |  |
|  |  | 0.30 | -7.93 (-13.3 to -2.54) | 0.004 |  |  |  |  |  |  |
| Deprivation PM | 4^a^ | 0.71 | -3.45 (-4.48 to -2.42) | <0.001 | 0.00 | 0.905 |  |  | -3.61 to -3.35 | n |
|  |  | 0.80 | -3.49 (-4.61 to -2.37) | <0.001 |  |  |  |  |  |  |
|  |  | 0.30 | -3.49 (-5.50 to -1.48) | <0.001 |  |  |  |  |  |  |

**Supplementary Table S13:** continued

|  |  |  | **Exercise performance % change** |  | **Heterogeneity** |  |  |  | **Sensitivity analysis – one out** | |
| --- | --- | --- | --- | --- | --- | --- | --- | --- | --- | --- |
| **Exercise category** | **Outcomes, n** | **R** | **Mean (95% CI)** | ***p*** | **I^2^-value** | ***p*** | **σ^2^effect** | **σ^2^study** | **Range (EP%_Δ_)** | **CI crossed**  **(y or n)** |
| Restriction AM | 3^a^ | 0.71 | -2.67 (-4.62 to -0.73) | 0.007 | 0.00 | 0.794 |  |  | -2.92 to -2.53 | y |
|  |  | 0.80 | -2.69 (-4.51 to -0.87) | 0.004 |  |  |  |  |  |  |
|  |  | 0.30 | -2.66 (-5.43 to 0.12) | 0.061 |  |  |  |  |  |  |
| Restriction PM | 5 | 0.71 | -4.11 (-7.40 to -0.82) | 0.026 | <0.001 | 0.855 | <0.001 | <0.001 | -4.57 to -3.80 | y |
|  |  | 0.80 | -4.11 (-7.00 to -1.22) | 0.017 |  |  |  |  |  |  |
|  |  | 0.30 | -4.12 (-9.18 to 0.94) | 0.087 |  |  |  |  |  |  |
| *Early restriction AM* | 0 |  |  |  |  |  |  |  |  |  |
| *Early restriction PM* | 2^a^ | 0.71 | -5.28 (-9.17 to -1.39) | 0.008 | 0.00 | 0.798 |  |  | -5.88 to -4.85 | y |
|  |  | 0.80 | -5.30 (-8.71 to -1.90) | 0.002 |  |  |  |  |  |  |
|  |  | 0.30 | -5.28 (-11.3 to 0.74) | 0.086 |  |  |  |  |  |  |
| *Late restriction AM* | 2^a^ | 0.71 | -2.92 (-6.83 to 0.98) | 0.143 | 0.00 | 0.507 |  |  | -5.78 to -2.31 | y |
|  |  | 0.80 | -2.98 (-6.57 to -0.61) | 0.103 |  |  |  |  |  |  |
|  |  | 0.30 | -2.88 (-8.71 to 2.94) | 0.333 |  |  |  |  |  |  |
| *Late restriction PM* | 0 |  |  |  |  |  |  |  |  |  |

**Abbreviations/definitions**: AM: ante meridiem; PM: post meridiem; Deprivation: participants did not sleep for an extended period of time (i.e., whole night); restriction: total sleep time ≤6 h in any 24 h period – this category is a combination of early-restriction, late-restriction, fragmented sleep and sleep restriction protocols not specified; Early restriction: participants delayed sleep (i.e., went to sleep at a later time); Late restriction; participants awakened earlier than normal. ^a^ All outcomes were from independent studies and meta-analysis was run without dependency levels (i.e., simple meta-analysis).

**
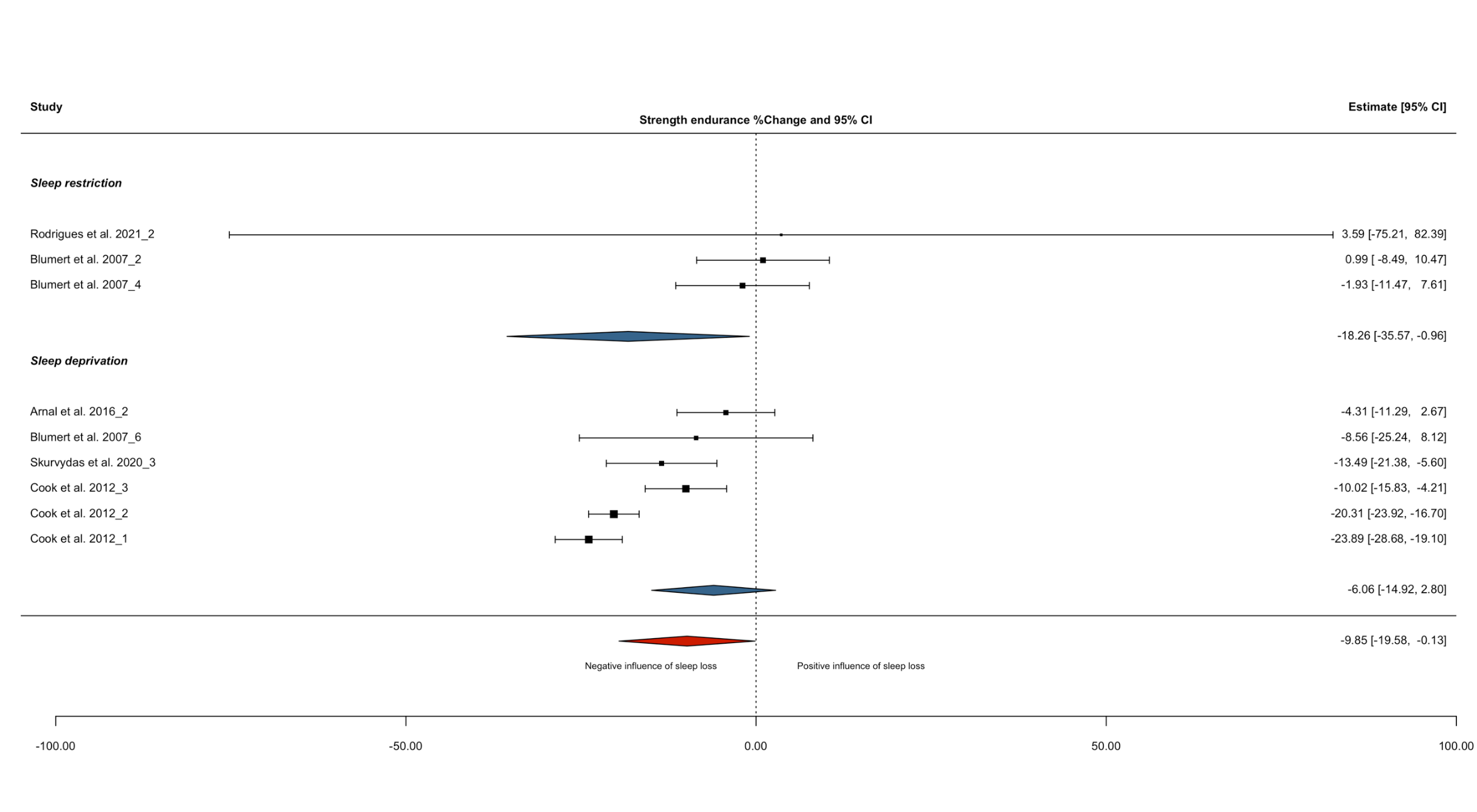
**

**Supplementary Figure S7:** Forest plot displaying the effect of sleep loss vs. normal sleep on the percentage change in strength-endurance performance. The size of the squares are proportional to the weight of the study. A negative effect estimate indicates a decrease in strength-endurance performance under the intervention condition (‘sleep loss’). Underscore numerical value (i.e., _1, _2, _3 etc.) identifies the different tasks performed at a given time; studies conducted by the same author in the same year are denoted by the addition of a capital ‘a or ‘b’ (i.e., A, B) to the citation. Deprivation: participants did not sleep for an extended period of time (i.e., whole night); sleep restriction: total sleep time ≤6 h in any 24 h period – this category is sleep restriction protocols not specified.

**Supplementary Table S14**: Sensitivity analysis of alternative levels of correlation coefficient (R) on strength-endurance performance meta-analysis results

|  |  |  | **Exercise performance % change** |  | **Heterogeneity** | |  |  | **Sensitivity analysis – one out** | |
| --- | --- | --- | --- | --- | --- | --- | --- | --- | --- | --- |
| **Exercise category** | **Outcomes, n** | **R** | **Mean (95% CI)** | ***p*** | **I^2^-value** | ***p*** | **σ^2^effect** | **σ^2^study** | **Range (EP%_Δ_)** | **CI crossed**  **(y or n)** |
| **Strength-endurance** |  |  |  |  |  |  |  |  |  |  |
| Overall | 9 | 0.71 | -9.85 (-19.6 to -0.13) | 0.048 | 85.4 | <0.001 | 26.7 | 46.1 | -11.2 to -8.71 | y |
|  |  | 0.80 | -9.81 (-19.4 to -0.19) | 0.047 |  |  |  |  |  |  |
|  |  | 0.30 | -10.3 (-20.5 to -0.06) | 0.049 |  |  |  |  |  |  |
| Deprivation | 6 | 0.71 | -6.06 (-14.9 to 2.80) | 0.139 | 45.6 | 0.255 | <0.001 | 23.0 | -6.92 to -2.78 | y |
|  |  | 0.80 | -6.11 (-15.0 to 2.78) | 0.138 |  |  |  |  |  |  |
|  |  | 0.30 | -5.74 (-13.7 to 2.23) | 0.123 |  |  |  |  |  |  |
| Restriction | 3 | 0.71 | -18.3 (-35.6 to -0.96) | 0.045 | 88.3 | 0.001 | 42.6 | <0.001 | -21.7 to -15.4 | y |
|  |  | 0.80 | -18.2 (-35.7 to -0.77) | 0.046 |  |  |  |  |  |  |
|  |  | 0.30 | -18.5 (-34.9 to -2.22) | 0.039 |  |  |  |  |  |  |
| **AM vs. PM** |  |  |  |  |  |  |  |  |  |  |
| Overall AM | 8 | 0.71 | -11.2 (-23.3 to 0.85) | 0.064 | 87.0 | <0.001 | 27.7 | 56.6 | -12.8 to -10.1 | y |
|  |  | 0.80 | -11.2 (-23.2 to 0.79) | 0.010 |  |  |  |  |  |  |
|  |  | 0.30 | -11.7 (-24.2 to 0.82) | 0.063 |  |  |  |  |  |  |
| Overall PM | 1 |  |  |  |  |  |  |  |  |  |
|  |  |  |  |  |  |  |  |  |  |  |
|  |  |  |  |  |  |  |  |  |  |  |
| **Sleep condition** |  |  |  |  |  |  |  |  |  |  |
| Deprivation AM | 5 | 0.71 | -7.06 (-22.9 to 8.82) | 0.285 | 60.5 | 0.172 | <0.001 | 53.7 | -8.44 to -1.56 | y |
|  |  | 0.80 | -7.11 (-23.1 to -8.83) | 0.283 |  |  |  |  |  |  |
|  |  | 0.30 | -6.90 (-22.8 to 8.98) | 0.294 |  |  |  |  |  |  |
| Deprivation PM | 1 |  |  |  |  |  |  |  |  |  |
|  |  |  |  |  |  |  |  |  |  |  |
|  |  |  |  |  |  |  |  |  |  |  |
| Restriction AM | 3 | 0.71 | -18.3 (-35.6 to -0.96) | 0.045 | 88.3 | 0.001 | 42.6 | <0.001 | -21.7 to -15.4 | y |
|  |  | 0.80 | -18.2 (-35.7 to -0.77) | 0.046 |  |  |  |  |  |  |
|  |  | 0.30 | -18.5 (-34.9 to -2.22) | 0.039 |  |  |  |  |  |  |
| Restriction PM | 0 |  |  |  |  |  |  |  |  |  |

**Definitions**: Deprivation: participants did not sleep for an extended period of time (i.e., whole night); restriction: total sleep time ≤6 h in any 24 h period – this category is sleep restriction protocols not specified.


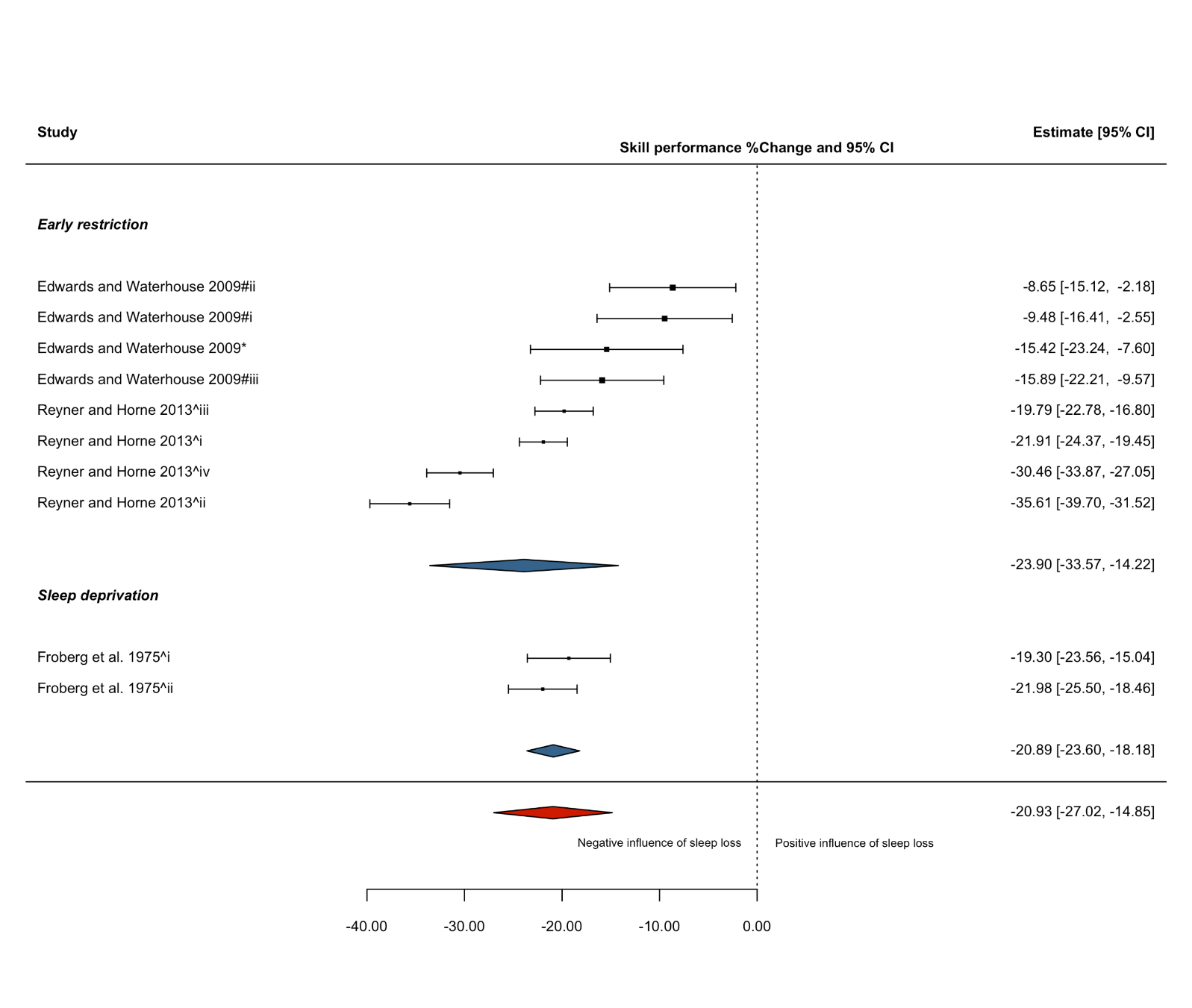


**Supplementary Figure S8:** Forest plot displaying the effect of sleep loss vs. normal sleep on the percentage change in skill performance. The size of the squares are proportional to the weight of the study. A negative effect estimate indicates a decrease in skill performance under the intervention condition (‘sleep loss’). Individual participant pools from a single study are denoted by the addition of a caret followed by a roman numeral (i.e., ^i, ^ii, ^iii etc.) to the citation; different sleep loss protocols (i.e., deprivation and late-restriction or early-restriction) from a single research study are denoted by the addition of a lower-case letter (i.e., a, b) to the citation; * or # indicates task performed in the AM or PM, respectively. * or # followed by a roman numeral value (i.e., *i, *ii, *iii) indicates that same task was performed at multiple times during AM or PM; underscore numerical value (i.e., _1, _2, _3 etc.) identifies the different tasks performed at a given time. Deprivation: participants did not sleep for an extended period of time (i.e., whole night); restriction: total sleep time ≤6 h in any 24 h period – this category is a combination of early-restriction, late-restriction, fragmented sleep and sleep restriction protocols not specified; Early restriction: participants delayed sleep (i.e., went to sleep at a later time). Note, the trials from Filipas et al. 2021 [109] (late restriction) and Cook et al. 2012 [63] (sleep restriction) are included in overall effect estimate.

**Supplementary Table S15:** Sensitivity analysis of alternative levels of correlation coefficient (R) on skill performance meta-analysis results

|  |  |  | **Exercise performance % change** |  | **Heterogeneity** |  |  |  | **Sensitivity analysis – one out** | |
| --- | --- | --- | --- | --- | --- | --- | --- | --- | --- | --- |
| **Exercise category** | **Outcomes, n** | **R** | **Mean (95% CI)** | ***p*** | **I^2^-value** | ***p*** | **σ^2^effect** | **σ^2^study** | **Range (EP%_Δ_)** | **CI crossed (y or n)** |
| **Skill** |  |  |  |  |  |  |  |  |  |  |
| Overall | 13 | 0.71 | -20.9 (-27.0 to -14.9) | <0.001 | 94.1 | <0.001 | <0.001 | 66.7 | -22.6 to -19.2 | n |
|  |  | 0.80 | -20.9 (-27.0 to -14.8) | <0.001 |  |  |  |  |  |  |
|  |  | 0.30 | -21.1 (-27.0 to -15.2) | <0.001 |  |  |  |  |  |  |
| **Sleep condition** |  |  |  |  |  |  |  |  |  |  |
| Deprivation | 2^a^ | 0.71 | -20.9 (-23.6 to -18.2) | <0.001 | 0.00 | 0.342 |  |  | -22.0 to -19.3 | n |
|  |  | 0.80 | -20.9 (-23.5 to -18.3) | <0.001 |  |  |  |  |  |  |
|  |  | 0.30 | -20.9 (-25.1 to -16.7) | <0.001 |  |  |  |  |  |  |
| Restriction | 11 | 0.71 | -21.0 (-29.1 to -12.9) | <0.001 | 95.4 | <0.001 | <0.001 | 90.2 | -23.2 to -18.6 | n |
|  |  | 0.80 | -20.1 (-29.1 to -12.8) | <0.001 |  |  |  |  |  |  |
|  |  | 0.30 | -21.1 (-29.1 to -13.2) | <0.001 |  |  |  |  |  |  |
| *Early restriction* | 8 | 0.71 | -23.9 (-33.6 to -14.2) | <0.001 | 94.8 | <0.001 | 2.84 | 78.5 | -25.0 to -21.0 | n |
|  |  | 0.80 | -23.8 (-33.6 to -14.1) | <0.001 |  |  |  |  |  |  |
|  |  | 0.30 | -24.0 (-33.4 to -14.5) | <0.001 |  |  |  |  |  |  |
| *Late restriction* | 1 |  |  |  |  |  |  |  |  |  |
| **AM vs. PM** |  |  |  |  |  |  |  |  |  |  |
| Overall AM | 4 | 0.71 | -14.2 (-26.7 to -1.68) | 0.037 | 87.0 | <0.001 | <0.001 | 38.6 | -19.2 to -13.5 | y |
|  |  | 0.80 | -14.1 (-26.2 to -2.10) | 0.033 |  |  |  |  |  |  |
|  |  | 0.30 | -14.6 (-27.2 to -2.07) | 0.034 |  |  |  |  |  |  |
| Overall PM | 9 | 0.71 | -22.9 (-29.7 to -16.0) | <0.001 | 93.8 | <0.001 | 3.67 | 55.6 | -23.4 to -20.8 | n |
|  |  | 0.80 | -22.8 (-29.7 to -15.8) | <0.001 |  |  |  |  |  |  |
|  |  | 0.30 | -22.9 (-29.7 to -16.2) | <0.001 |  |  |  |  |  |  |
| **Sleep condition** |  |  |  |  |  |  |  |  |  |  |
| Deprivation AM | 0 |  |  |  |  |  |  |  |  |  |
| Deprivation PM | 2^a^ | 0.71 | -20.9 (-23.6 to -18.2) | <0.001 | 0.00 | 0.342 |  |  | -22.0 to -19.3 | n |
|  |  | 0.80 | -20.9 (-23.5 to -18.3) | <0.001 |  |  |  |  |  |  |
|  |  | 0.30 | -20.9 (-23.5 to -16.7) | <0.001 |  |  |  |  |  |  |

**Supplementary Table S15**: continued

|  |  |  | **Exercise performance % change** |  | **Heterogeneity** | |  |  | **Sensitivity analysis – one out** | |
| --- | --- | --- | --- | --- | --- | --- | --- | --- | --- | --- |
|  | **Outcomes, n** | **R** | **Mean (95% CI)** | ***p*** | **I^2^-value** | ***p*** | **σ^2^effect** | **σ^2^study** | **Range (EP%_Δ_)** | **CI crossed**  **(y or n)** |
| Restriction AM | 4 | 0.71 | -14.2 (-26.7 to -1.67) | 0.037 | 87.0 | <0.001 | <0.001 | 38.6 | -19.1 to -13.5 | y |
|  |  | 0.80 | -14.1 (-26.2 to -2.10) | 0.033 |  |  |  |  |  |  |
|  |  | 0.30 | -14.6 (-27.2 to -2.07) | 0.034 |  |  |  |  |  |  |
| Restriction PM | 7 | 0.71 | -23.7 (-34.0 to -13.4) | 0.001 | 95.5 | <0.001 | 4.40 | 82.0 | -24.7 to -20.8 | n |
|  |  | 0.80 | -23.7 (-34.1 to -13.3) | 0.001 |  |  |  |  |  |  |
|  |  | 0.30 | -23.8 (-33.9 to -13.7) | 0.001 |  |  |  |  |  |  |
| *Early restriction AM* | 1 |  |  |  |  |  |  |  |  |  |
| *Early restriction PM* | 7 | 0.71 | -23.7 (-34.0 to 13.4) | 0.001 | 95.5 | <0.001 | 4.40 | 82.0 | -24.7 to -20.8 | n |
|  |  | 0.80 | -23.7 (-34.1 to -13.3) | 0.001 |  |  |  |  |  |  |
|  |  | 0.30 | -23.8 (-33.9 to -13.7) | 0.001 |  |  |  |  |  |  |
| *Late restriction AM* | 1 |  |  |  |  |  |  |  |  |  |
| *Late restriction PM* | 0 |  |  |  |  |  |  |  |  |  |

**Abbreviations/definitions**: AM: ante meridiem; PM: post meridiem; Deprivation: participants did not sleep for an extended period of time (i.e., whole night); restriction: total sleep time ≤6 h in any 24 h period – this category is a combination of early-restriction, late-restriction, fragmented sleep and sleep restriction protocols not specified; Early restriction: participants delayed sleep (i.e., went to sleep at a later time); Late restriction; participants awakened earlier than normal. ^a^ All outcomes were from independent studies and meta-analysis was run without dependency levels (i.e., simple meta-analysis).

**Title:** Effects of acute sleep loss on physical performance: A systematic and meta-analytical review

**Journal name:** Sports Medicine

**Authors:** Jonathan Craven, Danielle McCartney, Ben Desbrow, Surendran Sabapathy, Phillip Bellinger, Llion Roberts and Christopher Irwin

**Corresponding author:**

Jonathan Craven

School of Health Sciences and Social Work, Griffith University, Queensland, Australia, 4222.

Email: jonathan.craven@griffithuni.edu.au
